# Comparative Genomics of *Wolbachia* and the Bacterial Species Concept

**DOI:** 10.1371/journal.pgen.1003381

**Published:** 2013-04-04

**Authors:** Kirsten Maren Ellegaard, Lisa Klasson, Kristina Näslund, Kostas Bourtzis, Siv G. E. Andersson

**Affiliations:** 1 Department of Molecular Evolution, Cell and Molecular Biology, Science for Life Laboratory, Biomedical Centre, Uppsala University, Uppsala, Sweden; 2 Department of Environmental and Natural Resources Management, University of Western Greece, Agrinio, Greece; 3 Biomedical Sciences Research Center Al. Fleming, Vari, Greece; 4 Insect Pest Control Laboratory, Joint FAO/IAEA Programme of Nuclear Techniques in Food and Agriculture, Vienna, Austria; Université Paris Descartes, INSERM U1001, France

## Abstract

The importance of host-specialization to speciation processes in obligate host-associated bacteria is well known, as is also the ability of recombination to generate cohesion in bacterial populations. However, whether divergent strains of highly recombining intracellular bacteria, such as *Wolbachia*, can maintain their genetic distinctness when infecting the same host is not known. We first developed a protocol for the genome sequencing of uncultivable endosymbionts. Using this method, we have sequenced the complete genomes of the *Wolbachia* strains *w*Ha and *w*No, which occur as natural double infections in *Drosophila simulans* populations on the Seychelles and in New Caledonia. Taxonomically, *w*Ha belong to supergroup A and *w*No to supergroup B. A comparative genomics study including additional strains supported the supergroup classification scheme and revealed 24 and 33 group-specific genes, putatively involved in host-adaptation processes. Recombination frequencies were high for strains of the same supergroup despite different host-preference patterns, leading to genomic cohesion. The inferred recombination fragments for strains of different supergroups were of short sizes, and the genomes of the co-infecting *Wolbachia* strains *w*Ha and *w*No were not more similar to each other and did not share more genes than other A- and B-group strains that infect different hosts. We conclude that *Wolbachia* strains of supergroup A and B represent genetically distinct clades, and that strains of different supergroups can co-exist in the same arthropod host without converging into the same species. This suggests that the supergroups are irreversibly separated and that barriers other than host-specialization are able to maintain distinct clades in recombining endosymbiont populations. Acquiring a good knowledge of the barriers to genetic exchange in *Wolbachia* will advance our understanding of how endosymbiont communities are constructed from vertically and horizontally transmitted genes.

## Author Summary

Speciation in sexual organisms is defined as the inability of two populations to get viable offspring. Speciation in asexual, obligate endosymbionts is thought to be an indirect consequence of host-specialization. An important question is if divergent endosymbionts would start blending if the host barrier isolating them were removed. Here, we have studied *Wolbachia*, an abundant group of bacteria in the insect world. *Wolbachia* is classified into supergroups based on multi-locus sequence typing. We have sequenced the genomes from the *Wolbachia* strains *w*No and *w*Ha. These are particularly interesting since they belong to different supergroups yet co-occur as a double-infection in natural populations of *Drosophila simulans*. A comparative genomics study showed that *w*Ha and *w*No contain no uniquely shared genes. Instead, each strain shares unique gene functions with members of the same supergroup that infect other hosts. This unexpected finding suggests an alternative means of ecological speciation, indicating that speciation is not restricted to host-specialization but rather that related endosymbionts can coexist as separate species in the same host. Our study sheds light on the genomic divergence between different partners inhabiting the intracellular niche of the same host organism.

## Introduction

The increasing availability of genomic data for closely related strains and species enables bacterial population sizes and structures to be explored in far greater detail than was possible until now. A major question is whether asexually reproducing bacterial cells are organized into “clusters” that contain genetic diversity, yet are distinguishable from each other [1]–[4]. Such clusters can arise through geographic isolation or extreme habitat specialization [5]. Whether bacteria that are not separated by any physical or geographic barriers can evolve into distinct groups is less clear, but studies of free-living bacteria such as *Vibrio*, *Synechococcus* and *Bacillus* have suggested that the formation of sequence clusters correlate with ecological specialization [6]–[8]. Likewise, a recent study of thermophilic archaea indicated ongoing speciation and suggested that these species are maintained by ecological differentiation within hot springs [9]. Studying the mechanisms and selective forces that influence the organization of genetic diversity in unicellular organisms is important for our understanding of speciation processes.

In bacteria, recombination between incipient species can potentially be an important factor affecting speciation. In a speciation model whereby populations diverge mainly through neutral processes alone, sequence divergence depends on the ratio of recombination to mutation [10]–[12]. In an ecological model of speciation, adaptive and ecological divergence of incipient species instead depends on the ratio of the selection intensity against recombined, niche-determining genes from the other population to the recombination rate between these populations [13]–[16]. If however, the populations are geographically isolated they may diverge regardless of their potential to recombine. In any of these models, the distinctness of sequences of incipient species can be enhanced by periodic selection, the success of which depends on the rate of recombination within populations. Finally, recombination can be a source of adaptation whereby one species can acquire an adaptive gene from another species.

The rate at which substitutions are introduced into a genome by recombination relative to mutation events (*r/m*) varies by more than two orders of magnitude in bacteria [17]. The highest *r/m* ratios (>50) have been observed for oceanic bacteria of the SAR11 clade [18], which are the most abundant bacteria in the upper surface waters of the oceans and have been shown to lack the mismatch repair system [19]. The lowest *r/m* ratios (<0.5) have been associated with obligate host-associated bacteria, such as *Buchnera aphidicola* [20] and other endosymbionts, that have co-evolved with their hosts for hundreds of millions of years. Such long-term co-evolution serves as a strong physical barrier to gene exchange between bacteria adapted to different hosts. In effect, these highly specialized endosymbiont populations are perhaps best described as distinct taxonomic units, or species.

*Wolbachia* is an obligate intracellular symbiont infecting various species of arthropods and filarial nematodes, where it is maternally inherited through the germ line cells [21]. In arthropods, *Wolbachia* is most known for the ability to manipulate the reproduction of their hosts in various ways, which include induction of parthenogenesis, feminization, male killing, and cytoplasmic incompatibility (CI) [21]. In filarial nematodes *Wolbachia* is mutualistic and necessary for normal development and fertility [22]. In addition to these roles, several studies have emerged in recent years indicating that *Wolbachia* may also have other functions, such as providing ATP for the host [23], improving longevity [23] or fecundity [24], protection against viruses [25], [26] and uptake of iron [27]. Unlike maternally inherited mutualistic endosymbionts that have been co-evolving with their hosts, arthropod *Wolbachia* can be lost and gained from the host population and they show high recombination frequencies [17].

*Wolbachia* is currently defined as a single species, which is further classified into a number of divergent supergroups (A–N). The most well studied supergroups are A and B that infect arthropods and C and D that infect filarial nematodes [28], [29]. The supergroup classification scheme was originally proposed based on single-gene phylogenies [30], and more recently supported by multi-locus sequence typing [31]. Since these analyses suggested that *Wolbachia* supergroups represent genetically distinct clades, it is debated whether some or all of these groups should be designated different species [32]. However, due to high levels of recombination between super-groups in a few marker genes such as the surface protein *wsp* [33] and frequent exchange of phage DNA [34], it is unclear whether the super-group classification scheme is representative of the genomes overall. Moreover, no phenotypic traits have been identified that correlate with the separation of arthropod-infecting strains into different supergroups. On the contrary, strains of different supergroup affiliation may display similar phenotypic traits and host ranges. For example, double infections with super-group A and B strains have been found in many insects, and the induction of cytoplasmic incompatibility is common in both supergroups. The distribution of other phenotypic traits is less well investigated [23]–[27]. In the absence of strong host-specialization patterns, niche partitioning within hosts provides an alternative mechanism of speciation.

To evaluate the extent of recombination and identify the ecological and physiological features that may explain the separation into supergroups, genome data is required. However, the sequencing of obligate endosymbionts such as *Wolbachia* is not trivial, since these bacteria are often present in low abundance in their hosts and cannot be cultivated outside of their hosts. Some protocols specifically designed to extract DNA from *Wolbachia* have been developed in recent years [35], [36] but the preparation of enough DNA for sequencing is still very time-consuming for obligate host-associated bacteria with low infection densities.

Because of these challenges, genomic data is currently only available for a few *Wolbachia* strains. These are the two supergroup A strains, *w*Mel infecting *Drosophila melanogaster* [37] and *w*Ri infecting *Drosophila simulans* [38] and the genomes of one supergroup B strain, *w*Pip, from the mosquito *Culex quinquefasciatus* [39], and one supergroup D strain *w*Bm isolated from the nematode *Brugia malayi* [40]. Early draft genomes have also been presented for two other supergroup B strains, namely *w*AlbB infecting the mosquito *Aedes albopictus* [41] and *w*VitB infecting the parasitic wasp *Nasonia vitripennis* [42]. Genome sizes are small, in the range of 1.5 Mb. Recombination has been shown to be prevalent between strains that belong to supergroup A, suggesting that *Wolbachia* is a highly recombining intracellular community [38]. Genomes in the A and B-supergroups contain between 20 to 60 ankyrin repeat genes. Although it is generally thought that these genes play a key role in host-interaction processes and may be involved in the reproductive phenotypes, it has been difficult to pinpoint the particular functions of these genes.

*Wolbachia* strains *w*Ha and *w*No are especially interesting in the context of this discussion since they share several phenotypic traits, but belong to different supergroups. Importantly, both strains cause CI in their host *Drosophila simulans*, where they occur as natural double infections in populations on the Seychelles and in New Caledonia [43]. Strain *w*Ha has also been found as a single infection on Hawaii and in French Polynesia, but natural populations of *D. simulans* infected only with *w*No are very rare [44]. Several studies support the hypothesis that the double infection originated on the Seychelles and spread east to the Indo-Pacific islands, where after *w*No was lost from some populations [45]–[47]. Notably, a double-infection very similar to the one found for *D. simulans* is also found in the sister species *Drosophila sechellia*, which is endemic to the Seychelles. Furthermore, *D. simulans* and *D. sechellia* have very similar mitochondrial genomes, despite significant divergence in the nuclear genome. It therefore seems likely that the *Wolbachia* double-infection preceded the speciation event between *D. simulans* and *D. sechellia*. A recent study has estimated the time to a common ancestor of the *D. simulans* subcomplex (including *D. simulans, D. sechellia* and *D. mauritiana*) to be ∼242.000 years ago [48], suggesting that the co-infection originated at least a few hundred thousand years ago.

In this study, we present a new method for the preparation of DNA from *Wolbachia*, based on multiple-displacement amplification that enables genome data to be collected for *Wolbachia* strains with low infection densities. We have applied this protocol to the sequencing of the genomes of *Wolbachia* strains *w*Ha and *w*No that are co-infecting *D. simulans*. By comparative analysis of these and previously sequenced *Wolbachia* genomes, we have analyzed whether genomic features such as recombination, genome rearrangements and gene acquisitions could explain the separation of *Wolbachia* strains into distinct supergroups. The findings are discussed in light of the species concept for bacteria.

## Results

### Sequencing the *Wolbachia w*Ha and *w*No genomes

We developed a novel procedure for the isolation and amplification of *Wolbachia* DNA present in low quantity in the insect hosts. In brief, *Wolbachia* cells were purified from embryos of *Drosophila simulans* and multiple-displacement amplification (MDA) was performed directly on the isolated bacterial cells (see Materials and Methods). Single and 3 kb paired-end sequence reads were collected from the amplified DNA using the 454 sequencing technology and assembled *de novo*. The sequence coverage obtained from each data set was very large, and we therefore only used 10% to 30% of the data for assembly with Mira (Table 1). The proportion of single and paired-end reads that assembled was estimated to between 96–97% and 86–88%, respectively (Table S1). The 454 sequence reads in the assembly had mean and median sizes of 300 to 400 bp, whereas the median length of the 454 sequence reads that did not assemble was less than 100 bp (Table S1) and of lower quality (Figure S1).

**Table 1:** Sequencing data and purity.

Strain	Sequencing method	Total number of reads	Number of reads used for assembly	Avg. coverage in assembly	% *Wolbachia* reads*
wHa	454, single-end	764.450	80.497	30x	97.3
wHa	454, 3 kb paired-end	1.129.697 (702.509 pairs)	164.007 (101.384 pairs)	23x	99.4
wHa	Illumina, paired-end	14.297.126	1.462.711	225x	97.9
wNo	454, single-end	793.407	142.884	40x	99.3
wNo	454, 3 kb paired-end	1.141.093 (663.271 pairs)	333.604 (166.199 pairs)	38x	99.5
wNo	Illumina, paired-end	17.726.864	1.772.688	136x	98.3

* As estimated from mapping all reads to the complete *Wolbachia* genomes.

Illumina paired-end reads were mapped onto the assembly to correct for frameshift errors generated by the 454 technology. The overall coverage of the *w*Ha and *w*No genomes in the final assemblies was about 40 to 80-fold for the 454 data and 100 to 200-fold for the Illumina data (Table 1). All gaps were closed by PCR on non-amplified DNA, confirming the reliability of the scaffolds obtained from the sequence data of the amplified DNA. In two positions in the *w*Ha genome, located 20 kb apart and containing a long repeat of 7.5 kb with 5 genes, the PCR reactions failed from one side. However, single reads and read pairs supported the connection between the repeats and the unique sequences flanking each of the two copies.

This is the first demonstration that the MDA method can be applied in order to generate complete genome sequences from a small number of starting cells of uncultivable bacterial endosymbionts. The MDA method is known to produce amplification bias and chimeric reads when applied to single cells, which prevents genome closure. We considered the risk that such artifacts could have influenced the final genome sequence, but found these artifacts to be less dominant when multiple endosymbiont cells were used to start the reaction. Importantly, the entire *Wolbachia* genomes were represented by the sequence data in the final assemblies although coverage was unevenly distributed across the genome (Figure S2). The same coverage pattern was observed irrespectively of the method used for sequencing (Figure S2A–S2C), suggesting that the amplification bias is not random, but probably determined by the primer sets included in the amplification kit.

The fraction of chimeric reads in the single-end 454 sequence library was about 1%, which is considered normal according to the Newbler manual. As expected, the percentage was higher for the 454 paired-end reads, about 13–14% (Figure S3), but part of these chimeric read pairs might have been generated during library preparation rather than during the MDA reaction. Even though some regions have a higher amount of chimeric reads, we do not believe that they have had a significant effect on the assembly, since the coverage of these putative chimeric reads closely follow the coverage distribution of non-chimeric reads and hence regions with high amounts of chimeric reads also have high amounts on non-chimeric reads (Figure S3). We conclude that the overall fraction of chimeric reads was too low to have an effect on the assembly.

In retrospect, we mapped the individual sequence reads back to the *Wolbachia* genome and estimated that more than 97% of all reads represented *Wolbachia* DNA (Table 1). The remaining few percent was mostly derived from mitochondrial DNA from *Drosophila simulans*, with little or no nuclear DNA in the preparation. However, a manual search in the non-assembled sequences produced by the Mira assembly software of the *w*No sample revealed the presence of *w*Ha reads in low quantities (Figure S2D). Since most of the *w*Ha genome was covered but no nuclear DNA was detected, it is unlikely that these reads were derived from bacterial sequences integrated into the host nuclear genome. Rather, we believe that there may have been a slight contamination of *w*Ha during sample preparation and sequencing, or that the double-infected line from which wNo was generated was not completely cured of *w*Ha. No *w*No reads were found in any of the amplified DNA samples for *w*Ha. In conclusion, the large majority of sequence data generated by the MDA method was of good quality, not chimeric and covered the entire genome with little or no contamination of nuclear DNA.

### Genome features

The *w*No and *w*Ha genomes are 1.3 Mb in size and contain circa 1,000 genes, which corresponds to a coding density of about 80% (Table 2). This is comparable to the fraction of coding DNA in the previously sequenced *Wolbachia* genomes with the exception of *w*Mel, in which a larger fraction of short open reading frames were identified as genes, resulting in a higher estimated coding density of 94% (Table 2). As in all previously sequenced genomes of arthropod *Wolbachia*, several phage-derived fragments were identified. The *w*Ha genome contains two such regions, one of which encodes a nearly complete WO-phage. The *w*No genome contains four segments of putative phage origin, of which the two larger fragments together contain all conserved parts of the WO-phage. Pseudogenes were identified in all four phage segments in the *w*No genome, making it unlikely that any of them could individually produce phage particles. We observed a similar number of putatively functional IS elements in the two genomes, 12 in wHa and 14 in wNo (Table S2). Additionally, we identified 58 defective IS elements in *w*Ha, of which 17 were defective IS3 elements. No defective IS3 elements were present in the *w*No genome, which only contained a total of 14 defective IS elements.

**Table 2:** General features of *Wolbachia* genomes.

	*w*Mel	*w*Ri	*w*Ha	*w*No	*w*Pip	*w*Bm
Super-group	A	A	A	B	B	D
Host	*D. melano-gaster*	*D. simulans*	*D. simulans*	*D. simulans*	*C. quinque-fasciatus*	*B.malayi*
Size (bp)	1.267.782	1.445.873	1.295.804	1.301.823	1.482.455	1.080.084
G+C content	35.46	35.4	35.34	34.5	34.6	35.2
Coding sequences	1195	1150	1010	1040	1275	805
Coding density	0.94	0.8	0.78	0.8	0.86	0.745
Average gene size	851	976	1000	1013	951	899
rRNA	1 of each	1 of each	1 of each	1 of each	1 of each	1 of each
tRNA	34	34	34	34	34	34
Pseudogenes	74	114	93	95	110	98
Reference	[37]	[38]	This study	This study	[39]	[40]

### The *Wolbachia* core genome phylogeny

**Figure 1:**
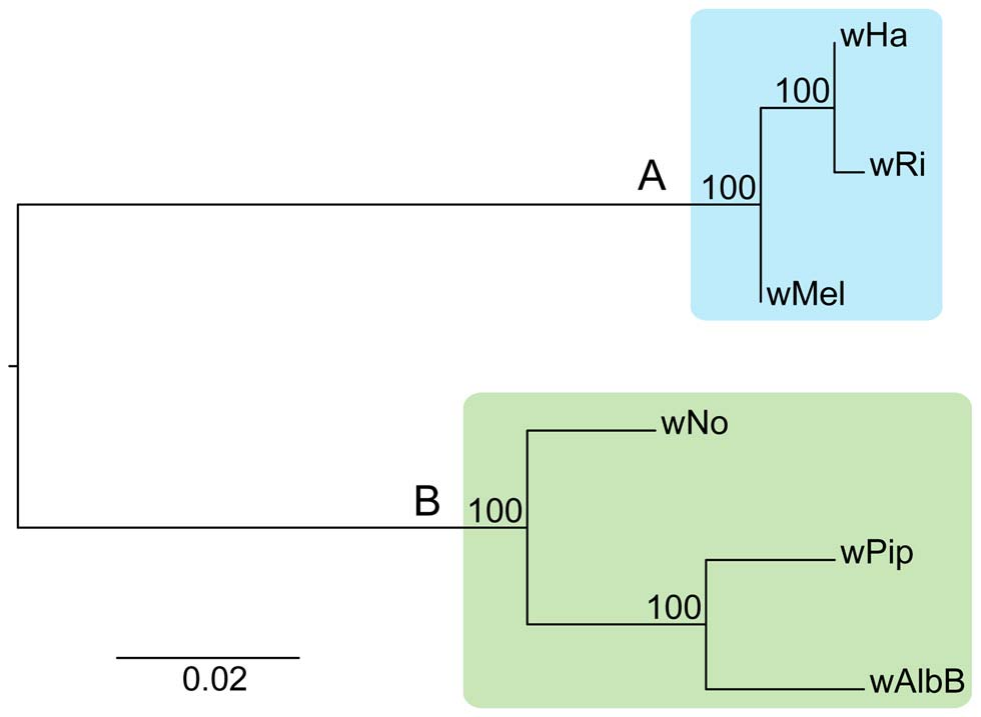
Phylogenetic relationships of six *Wolbachia* strains. The phylogenetic tree was inferred using the maximum likelihood method from a concatenated alignment of 660 single copy orthologous genes. Numbers on the branches represent the support from 1000 bootstrap replicates. The blue box indicates the strains from supergroup A and the green box indicates the strains from supergroup B.

**Figure 2:**
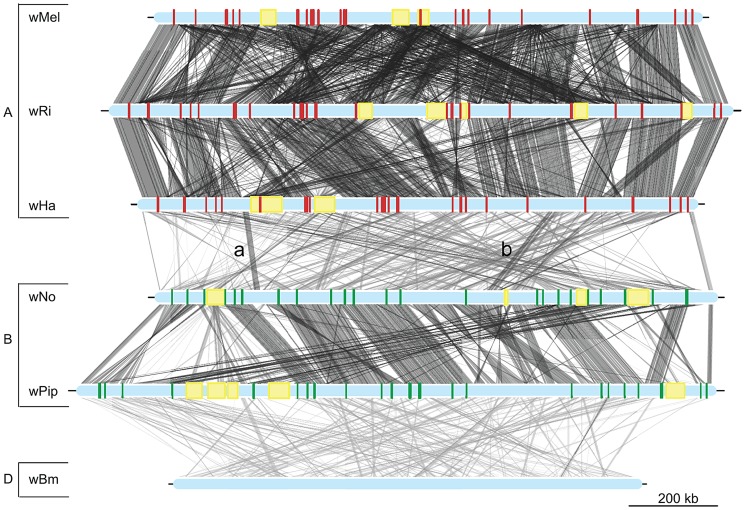
Genomic overview of the similarity between completely sequenced *Wolbachia* strains. Red boxes show the location of supergroup A specific genes (only in the supergroup A strains, *w*Mel, *w*Ri and *w*Ha), whereas green boxes indicate the position of supergroup B specific genes (only in the supergroup B strains *w*No and *w*Pip). The yellow boxes represent the location of prophage elements in supergroup A and B strains. The supergroup D strain *w*Bm is included for comparison. Similarity between sequences is indicated by the intensity of the grey lines, where darker is more similar. The lines between the wNo and wHa genomes with the small letters “a” and “b” indicate two regions with atypical synteny and sequence conservation.

Adding the *w*Ha and *w*No genomes to the previously produced draft and complete *Wolbachia* genome sequences, we tested the robustness of the supergroup classification scheme using three A-group (*w*Ha, *w*Ri, *w*Mel) and three B-group (*w*No, *w*Pip, *w*AlbB) strains. We identified 660 orthologous core genes present in all six genomes. A phylogenetic analysis with the maximum likelihood method based on a concatenated alignment of the core genes supported the separation of the two supergroups with 100% bootstrap support, and further suggested that *w*Ri and *w*Ha are most closely related within the A-group, and that *w*Pip and *w*AlbB are sister taxa within the B-group (Figure 1). Consistently, gene order structures were largely conserved within supergroups, but highly scrambled in all pair-wise comparisons of A- and B-group genomes (Figure 2). Thus, the classification of these strains into two supergroups is strongly supported by both the sequences and the architectures of the *Wolbachia* genomes.

### Recombination within and between *Wolbachia* supergroup A and B

To test the hypothesis that recombination mediates cohesion within supergroups but is reduced between supergroups, we examined the topologies of single gene trees, studied the spread of sequence divergence estimates, and inferred the relative fraction of intragenic recombination events both within and across the supergroup boundaries.

#### Single gene trees

**Figure 3:**
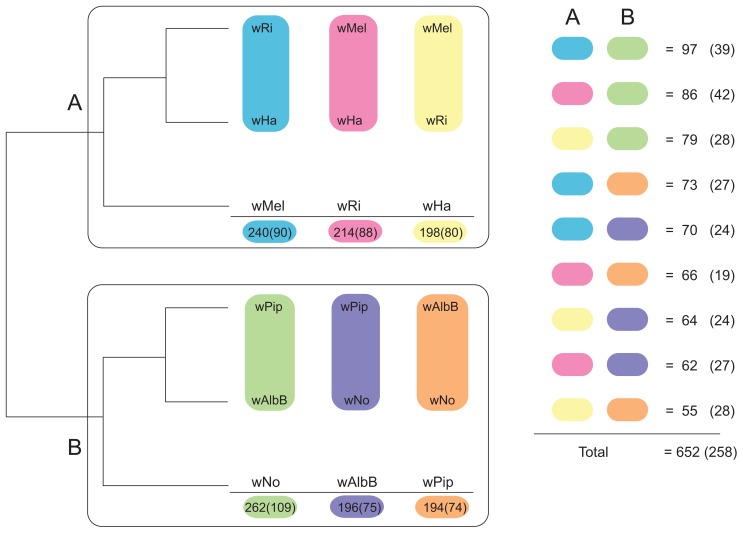
Schematic figure of individual tree topologies inferred from single-copy orthologs. Phylogenetic trees were inferred using the maximum likelihood methods from 660 single-copy gene orthologs, 652 of which supported the division between super-group A and B. The schematic tree summarizes the distribution of the 652 gene trees, where the number of trees supporting a topology within each super-group is shown. The coloured blocks on the right show the number of trees found for each of the 9 possible topologies with separation of the supergroups. The numbers were obtained by clustering individual trees based on weighted Robinson-Fould distances. Numbers in parenthesis represent the trees where all nodes have a bootstrap value higher than 75.

We first constructed single gene trees with the maximum likelihood method and clustered the tree topologies based on pair-wise weighted Robinson-Fould distances. For 652 of the 660 trees, the separation of the A- and B-group strains was consistent, but among these the 9 possible topologies were represented in almost equal abundance (Figure 3). Additionally, each of the three possible topologies within each supergroup accounted for about one third (29% to 40%) of the 660 trees (Figure 3), which is close to 33% as expected if the taxa are randomly clustered within the two supergroups. However, a few more trees indicated a clustering of *w*Ri and *w*Ha to the exclusion of *w*Mel (240 trees, 36%), and of *w*Pip and *w*AlbB to the exclusion of *w*No (262 trees, 40%) (Figure 3). Thus, the clustering of *w*Ri and *w*Ha, and of *w*Pip and *w*AlbB in the concatenated tree (Figure 1) may be due to a higher fraction of genes that support these relationships rather than a higher overall sequence similarity. In a more stringent analysis, we analyzed a subset of 260 trees with bootstrap support values above 75% for all nodes (for a few example trees, see Figure S4). We saw that the percentage of trees representing each topology remained similar to those in the total data set (Figure 3) and thus no particular topology was an artifact of low resolution. Taken together, our results indicate that the supergroup division is highly robust, but that about two thirds of the trees have a topology within supergroups that does not reflect evolution through vertical decent, as expected if the strains belonging to the same supergroup are highly recombinogenic (Figure 3).

In contrast, only sporadic cases of recombination between strains of different supergroups were detected in the analysis. Of the 660 core genes, 8 genes generated a topology that was incongruent with the supergroup classification scheme. Five of these genes (*nuoJKLN* and *ccmE*) code for proteins involved in the respiratory chain complex and are co-located in a long syntenic segment that is highly similar in sequence between the A and B-supergroup strains (marked as b in Figure 2). In *E. coli*, all 14 *nuo* genes are located in the same cluster [49], but in *Wolbachia* they are split up into 7 different clusters: *nuoABC, nuoD, nuoE, nuoF, nuoGH, nuoI* and *nuoJKLN*. The three additional core genes that yielded a topology that conflicted with the supergroups were *coxB*, which code for cytochrome oxidase subunit B in the respiratory chain complex, *mutS* and *ribE*.

**Figure 4:**
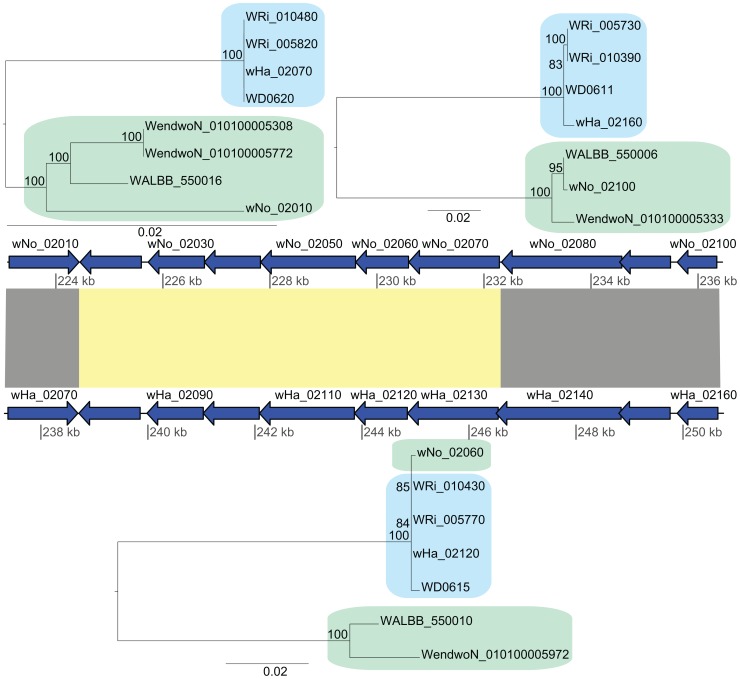
Recombination of chromosomal genes between the supergroup A and B strains. The yellow block indicates the six genes that produce a gene topology indicative of recombination between *w*No and the A-group strains (tree topology shown below the gene order comparison plot). The upstream and downstream flanking genes show the normal separation of the strains into two super-groups (tree topologies shown above the gene order comparison plot).

Additionally, we identified a cluster of genes encompassing 13 kb that showed an atypically high sequence similarity between *w*Ha and *w*No (marked as a in Figure 2). This segment is not present in the *w*Pip genome and was therefore not included in the set of 660 single gene orthologs. A separate phylogenetic analysis revealed near identity for six co-located genes in *w*No and the three supergroup A genomes, which contrasts with the clear separation of the A and B-group strains in trees produced from the flanking genes (Figure 4). The high sequence similarity for these genes provides indirect evidence for a recent transfer of a long DNA segment between supergroups.

**Figure 5:**
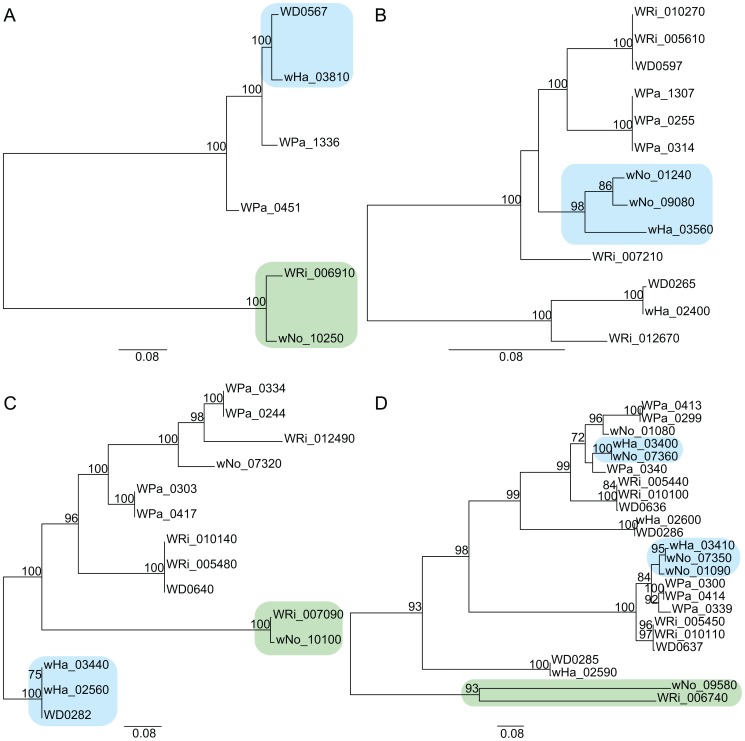
Horizontal transfer of bacteriophage genes between the supergroup A and B strains. Phylogenetic trees were inferred using the maximum likelihood methods from four prophage-associated genes A) “Late control D”, B) “Terminase”, C) “Baseplate W”, D) “ANK.”.

Finally, we inferred several cases of gene transfers of phages between strains of different supergroups. All genomes contain prophages of similar organization (Figure S5) and our single gene phylogenies of phage genes revealed a mosaic pattern, consistent with repeated recombination of phage genes within and across the supergroup boundary. A transfer of the *orf*7 phage gene between *w*Ha and *w*No was previously indicated by PCR analysis [34]. However, none of the *orf*7 sequences in the *w*Ha and *w*No genomes were identical to the PCR-amplified sequences. Instead, we observed near sequence identity of a few other phage genes in the *w*Ha and *w*No genomes. For example, the head genes of *w*Ha-WO2 were most closely related to prophage genes from the *Wolbachia* B-group strains *w*No and *w*Pip (Figure 5C), whereas the baseplate and tail genes were most closely related to the homologous genes in the WO-A and WO-B prophages in the A-group strain *w*Mel (Figure 5A and 5B). We also noted a close relationship of the *w*No-WO4 genes to the *w*Ri-WOC prophage (Figure 5D), consistent with previous analyses of a smaller gene set [38].

#### Large spread of sequence divergence levels

To quantify the heterogeneity in sequence divergences for the individual genes, we determined substitution frequencies at synonymous sites (dS) for all pairwise combinations of the 660 orthologs. For genes that undergo high frequencies of recombination, the dS value does not necessarily reflect the frequency of mutations, but it can still be used as a proxy of sequence divergence levels. According to this analysis, the A-group strains (median dS-values = 0.03) were less divergent than the B-group strains (median dS-values = 0.06–0.08) (Table S3).

**Figure 6:**
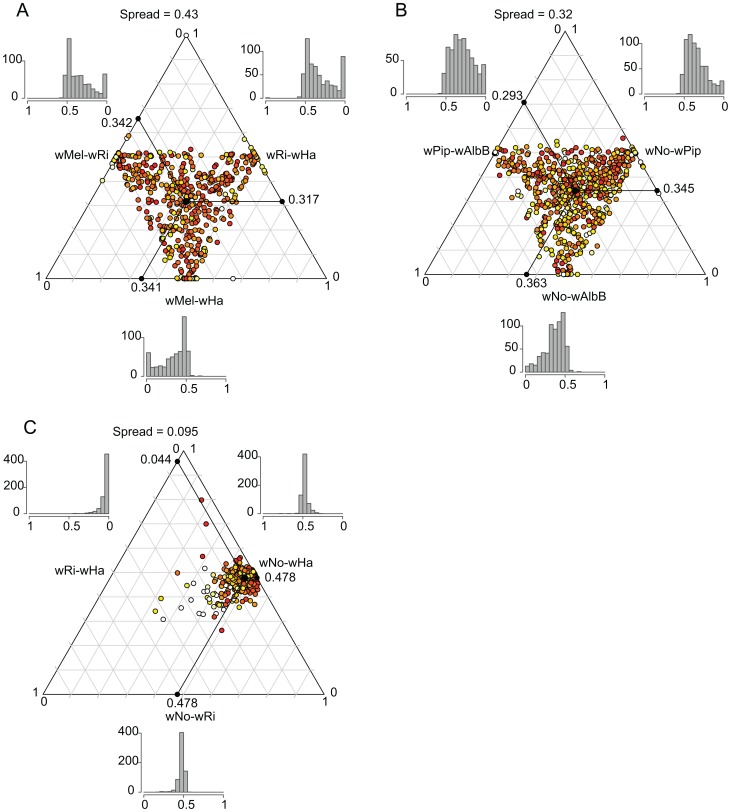
Ternary plot of sequence divergence levels at synonymous sites. The plot shows the variation in synonymous substitution frequencies for the 660 single-copy orthologs in A) supergroup A *Wolbachia* strains, B) supergroup B *Wolbachia* strains and C) *D. simulans* infecting *Wolbachia* strains. Each dot in the plot represents one gene. Absolute dS-values have been transformed to relative values between 0 and 1 and the mean relative dS-value for each pair is shown on each axis. The spread represents the median distance to the mean point. The color of each dot represents the maximum absolute dS-value among the 3 pairs, ranging from light yellow (low values) to red (high values). The histograms show the frequency of relative dS-values within each pair.

We plotted the relative pairwise dS-values for each of the 660 genes in a ternary plot for supergroup A and B separately to illustrate the consistency of the divergence estimates (Figure 6A and 6B). If two out of three strains are consistently more similar to each other than what either is to the third strain, the dots will be concentrated in one spot of the triangle (the average of the relative dS-values), as previously observed for *Rickettsia* (see Fig. 2B in [38]). In contrast, if there is no consistent signal in the data, as observed for the highly recombinogenic species *Neisseria meningitidis*, the dots will be distributed across the triangle (see Fig. 2C in [38]). For *Wolbachia*, the relative dS values were widely distributed across the triangle, with the spread estimated to 0.43 and 0.32 for the A and B-group strains, respectively (calculated as the distance from the average point in the plot as described by [38]) which is similar to a previous estimate of 0.42 for the A-group strains *w*Ri, *w*Mel and *w*Uni [38]. The large spread of the values indicate that the individual dS-values are in many cases far off from the average relative value in both the A and B-group strains, as expected when homologous recombination overrides the vertical inheritance pattern of single nucleotide substitutions.

For comparison, we also included a plot of the dS-values for the 660 genes in the three *Wolbachia* strains infecting *D. simulans* (*w*Ha, *w*Ri and *w*No). The dS values for *w*Ha and *w*Ri was 20-fold lower than the dS value for pairs that include *w*No (dS = 0.57–0.58, Table S3), and the spread of the dS values was estimated to only 0.095 (Figure 6C). This suggests that the two supergroups diverged from each other mainly by the accumulation of nucleotide substitutions, since only very few genes show a pattern of divergence that conflict with the overall divergence pattern between the strains.

#### Relative frequency of recombination to mutation

We used ClonalFrame to estimate the overall ratio (*r/m*) at which recombination and mutation events generate a substitution, for the concatenated alignment of all 660 single copy orthologs present in all six *Wolbachia* strains. Overall, the *r/m* ratio was estimated to 3.57 (95% credibility region 3.43–3.71). We also quantified the *r/m* ratio for each branch in the tree using ClonalFrame, including only positions where the probability of substitution via mutation or recombination was higher or equal to 0.95. The ratios were consistently high, ranging from 2.33 on the branch to *w*Mel to 8.82 on the branch to *w*Ri (Table 3). This suggests that the likelihood that a base difference is due to a recombination event is on the average 2 to 8 times higher than the likelihood for a mutation event to cause a substitution, in any of the strains in our analysis.

**Table 3:** Estimates of recombinations and mutations occurring in each strain.

	Run 1			Run 2		
Strain	r/m	No. Mut	No. Rec	r/m	No. Mut	No. Rec
*w*Ri	8.82	481	4243	8.59	492	4224
*w*Ha	6.46	572	3697	6.35	583	3703
*w*Mel	2.33	562	1311	2.30	573	1319
*w*Pip	6.71	785	5266	6.64	795	5277
*w*AlbB	6.01	889	5346	6.10	882	5344
*w*No	4.40	808	3559	4.24	839	3554

#### Intragenic recombination events

While our analyses of single-gene phylogenies and divergences at synonymous sites indicated very little recombination between supergroup A and B, we considered the possibility that smaller fragments of genes could be exchanged. To estimate the number of genes affected by recombination events within and between supergroups, we used four different methods (NSS, MaxChi, Phi and geneconv) to reduce the risk of identifying false positives. A total of 120 of the 660 single copy orthologs gave significant results with all four methods and an additional 73 genes gave significant results with three of the methods. Thus, about one third of all genes carried a signal of past recombination events according to this analysis. This represents a conservative estimate since events that span across the length of the gene will not be detected by these methods.

In this analysis, geneconv is the only program that identifies which sequences in the alignment took part in the recombination event. We therefore evaluated the 176 genes for which recombination was detected with geneconv and at least two additional methods, in order to determine whether the intragenic recombination events had occurred within or between supergroups. Further, we ran geneconv with different mismatch penalty settings, to optimize for detection of recombination of both highly similar and more divergent pairs of sequences (see materials and methods). While the number of genes in which recombination was detected was relatively similar regardless of the geneconv settings, the sizes of the recombining fragments were more variable (Table S4, Figure S6). Recombining fragments were in the range of 300 to 700 bp for the A-group strains and 200 to 600 bp for the B-group strains. The inferred recombination fragments between supergroups were much shorter, in the range of 70 to 180 bp (Table S4).

Regardless of recombination detection method, short recombination events may go undetected in comparisons of highly similar sequences, such as of strains within supergroups. The number of intragenic recombination events within supergroups is therefore likely to have been underestimated. In contrast, the power to detect recent recombination events is relatively higher between strains of different supergroups due to the higher level of sequence divergence, particularly for long fragments. Therefore, we can confidently conclude that recombination between supergroups does take place, but appears to be restricted to small fragments. In effect, the total number of basepairs affected by recombination events across supergroups is several fold smaller than those affected by recombination events within supergroups. Moreover, since recombination events between supergroups have accumulated during a longer time period, we also conclude that the rate of recombination between supergroups is lower than within.

#### Recombination, sequence divergence, and functional categories

There may be several reasons why the frequencies of recombination were reduced between strains of different supergroups, one of which is that the high levels of divergence between the supergroups could serve as a barrier to recombination and restrict recombination events to a few highly conserved genes or domains. To test this hypothesis, we examined whether the 111 genes affected by recombination events across supergroups (Table S4) were less divergent than genes in which no such events had been detected. To this end, we estimated the pairwise genetic divergence between *w*No and all other strains for genes where recombination was detected between at least one of the other two B-supergroups strains (*w*Pip and *w*AlbB) and at least one of the three supergroup A strains (n = 51).

We found that the divergence between *w*No and the A supergroup strains was not significantly different for these genes compared to the genes (n = 549) not affected by such recombination events (Mann-Whitney test, *w*Mel p = 0.389, *w*Ri p = 0.3622, *w*Ha p = 0.475). However, the pairwise divergences between *w*No and each of the B-supergroup strains were significantly larger for the genes with recombination across the supergroups compared to the rest of the data set (Mann-Whitney test, p<<0.01). Similar results were obtained when any of the other strains were used in the same manner as *w*No (data not shown). These results suggest that recombination across supergroups does not only occur in genes with low divergence between supergroups. However, when such events occur they give rise to higher divergence between strains from the same supergroup and they can lead to an increased overall divergence measure of the genes even though many of the recombination tracts are short in size.

Additionally, we could not detect a bias towards certain functional categories for genes affected by recombination events between supergroups (Figure S7). We conclude that recombination between strains of different supergroups is not restricted to highly conserved genes or targeted to particular gene functions.

### Horizontal gene transfers and functional novelty

Novel gene acquisitions may confer the ability to inhabit new niches. In the case of endosymbionts, the acquisition of a new gene might potentially broaden the host range, but could also lead to ecological specialization within the existing host. For example, the uptake of a novel gene might contribute to the physical separation of strains with and without the new gene, leading to speciation. To investigate this hypothesis, we examined gene content differences between the two supergroups.

In total, we identified 33 and 24 protein clusters that were specific to the A- and B-group genomes, respectively (Tables S5, S6). A comparison of the number of protein clusters solely present in the A- or B-supergroup strains to the number of protein clusters found in any other combination of three strains showed that the supergroup specific protein clusters are largely over-represented (Figure S8).

**Figure 7:**
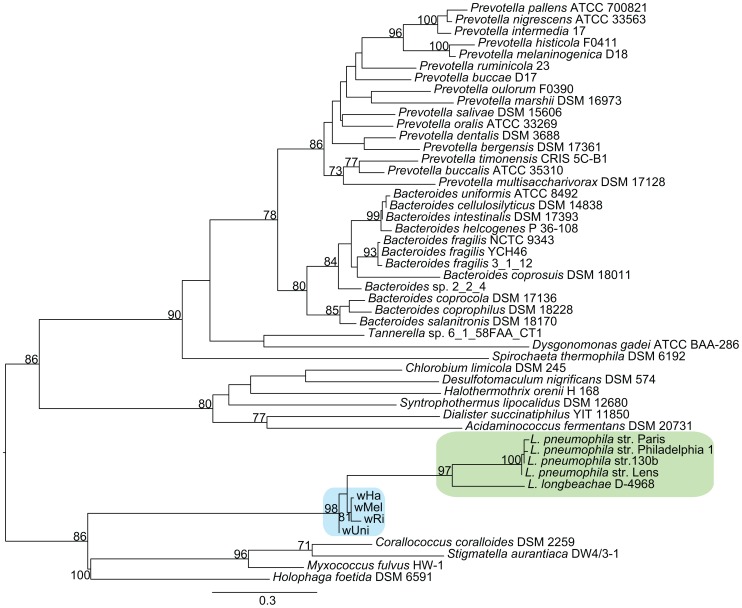
Phylogenetic analysis of the ArgR repressor gene. The phylogenetic tree was inferred using the maximum likelihood method based on a protein alignment of the ArgR repressor from many different bacterial species. Numbers on the branches represent the support from 1000 bootstrap replicates. The blue box indicates the *Wolbachia* strains from the supergroup A strains, and the green box indicates *Legionella* species.

Functional categorization of these clusters identified a few particularly interesting acquisitions in the A-group strains of genes putatively involved in the regulation of arginine transport systems (*argR*), stress response (*cydAB*) and modulation of host cellular functions (*fic*). Phylogenetic analyses revealed sequence similarities to several other intracellular bacteria, such as *Legionella, Rickettsia* and *Chlamydia*, indicating that these genes may serve a role for the intracellular lifestyle (Figure 7, Figure S9).

As in *Legionella pneumophila*, the gene for the arginine repressor ArgR is co-located with three genes for an arginine ABC transporter and phylogenetic reconstruction confirmed the close affiliation between *Wolbachia* and *Legionella* of the entire cluster of four genes (Figure 7, data not shown). Previous studies of other pathogenic bacteria have shown that arginine may be associated with virulence. Additionally, arginine can be converted to nitric oxide by the host as part of the innate immune response. In *Legionella*, the expression of the genes for the arginine repressor and transporter is sensitive to the presence of L-arginine and derepression is observed during intracellular growth [50]. In analogy, we infer that the *Wolbachia* ABC-transporters are expressed when the concentration of arginine is low, stimulating uptake of arginine through the ABC-transporters.

The Fic domain proteins solely present in the A-group strains are particularly interesting since their homologs in other bacteria have been shown to be secreted into the host cell cytoplasm to modify host regulatory GTPases [51], thereby causing the disruption of the host actin cytoskeleton [52], [53] or host cellular rearrangements [54]. The ability to manipulate host GTPases is most likely a general feature of all proteins containing this protein domain since it has also been reported in the distantly related Fic-domain containing human HYPE protein [53].

The comparison of gene contents also indicates possible differences in cell division and lipid II biosynthesis due to gene loss in the B-group strains. For example, the *ftsWIBL* genes, which are involved in these processes in *E. coli* are present in the A-group strains, but absent from the B-group strains and present only as pseudogenes in *w*Bm. Additionally, the *murC* gene, which catalyses the attachment of the first amino acid to the glycan, has been split into two genes located distantly from each other in the genomes of all B-group strains, including *w*VitB. One of the two genes encodes the N-terminal domain and the other encodes the C-terminal domain fused to a recombinase zinc beta ribbon domain (PFAM: PF13408) (Figure S10). Interestingly, experimental evidence has shown that a lipid-II-like molecule is synthesized in the supergroup B *Wolbachia* strain *w*AlbB [55], suggesting that the *murC* gene function is present despite the separation of the sequences encoding the functional domains into two genes.

Uniquely present in the B-group strains is a cluster of genes encoding outer membrane proteins which are found in two to three copies in each of the supergroup B genomes, including *w*VitB. Located at the corresponding genomic position in the A-group strains is a non-coding region of approximately 1 kb, which does not show any significant sequence similarity to genes in the B-group strains (Figure S11A). Eight of the nine proteins in this cluster contain PFAM domains annotated as outer-surface proteins, including the family to which the *Wolbachia* surface protein (*wsp*) belongs (PF01617). A phylogenetic analysis revealed a clustering of genes between the strains, rather than within the genome of one strain, suggesting that gene duplication occurred before divergence of these B-group strains (Figure S11B). Short sequence fragments with significant similarity to these surface proteins were identified in the *w*Bm genome, indicating loss from supergroup A. However, since no homologs outside *Wolbachia* supergroup B could be identified, the origin and function of this outer membrane protein family remain to be determined.

## Discussion

Evolutionary and functional studies of adaptations to the intracellular environment have previously been hampered by technical challenges associated with the inability to cultivate these bacteria outside their host cells, making it difficult to obtain DNA in large quantities and of enough purity for genome sequencing projects. To bridge this gap, we present a protocol with the potential to enable genomic studies of endosymbiotic bacteria without the need for laborious cultivation methods. Our approach relies on the purification of endosymbionts from their hosts, followed by whole genome amplification to increase the quantity of DNA. We have shown that although only small quantities of bacterial cells were used as the starting material, assembly and gap closure was not hindered by the drawbacks normally encountered with whole-genome amplification of single bacterial cells. Using this method, we have sequenced the genomes of two *Wolbachia* strains, *w*Ha and *w*No, that co-infect *D. simulans*. In a comparative genome analysis that included these and other *Wolbachia* genomes, we have corroborated the genetic separation of the *Wolbachia* supergroups A and B by a phylogenetic analysis of 660 concatenated core genes, suggesting that they should indeed be considered different species.

Discussions about the bacterial species concept have largely focused on the criteria used to define the species boundary [56], [57]. The most commonly used species definition is based on 16S rRNA sequence similarity, with the cut-off arbitrarily set to 97%, although 99% identity has also been suggested as a criterion of taxonomic species demarcation [58]. The *Wolbachia* strains investigated here are 97–98% identical in the 16S rRNA gene between supergroups and 99% identical within supergroups, thus representing a border-line case based on the 16S rRNA species definition.

Conceptually, bacterial species should also (*i*) fall into well-supported sequence clusters, (*ii*) evolve under cohesive processes within the species, (*iii*) be ecologically distinct and (*iv*) be irreversibly separated from each other [15], [59]. Our comparative genomics study shows clearly that *Wolbachia* supergroup A and B strains evolve as distinct clusters. Below, we discuss whether any of the other three concepts are also applicable to *Wolbachia*.

### Are *Wolbachia* strains within each supergroup evolving under cohesive processes?

High recombination frequencies were previously estimated for strains belonging to super-group A [38], and confirmed in this study in both supergroup A and B. Single-gene phylogenies showed all possible divergence patterns for strains within each supergroup in nearly equal proportions, and the spread of the relative dS values for individual genes within supergroups was very high (0.3–0.4), which is in the range of the naturally competent and highly recombinogenic bacterial pathogen *Neisseria meningitidis* (Spread = 0.34) [38]. Thus, there is a very strong bias for substitutions caused by recombination within *Wolbachia* supergroups, which suggests that there is very little selection against recombination within *Wolbachia* supergroups, consistent with the species concept.

### Are *Wolbachia* strains from supergroups A and B ecologically distinct?

For endosymbionts, co-evolution with hosts is thought to generate a physical barrier that leads to the evolution of ecologically distinct species. This is exemplified by a strong congruence of *Wolbachia* and host phylogenies for nematode-infecting strains [60], [61]. However, for *Wolbachia* strains infecting insects, host and endosymbiont phylogenies are generally not congruent [30], [31], [62]. Strains of different supergroups can infect the same host species, as exemplified by *w*Ha and *w*No in this study, just as strains of the same supergroup, such as *w*Ha and *w*Mel, can infect different host species. Furthermore, there is no simple association between supergroup affiliation and reproductive disorders, since strains of both supergroups are capable of inducing for example cytoplasmic incompatibility.

Yet, our analysis shows that there are differences in gene content between supergroup A and B, which are likely to influence the interactions with the host and the surrounding environment. Notable among the A-group specific functions are genes for uptake of arginine, tolerance to stress and secretion of proteins involved in the modulation of host cellular functions, whereas the B-group specific gene set included genes for outer surface structures. Thus, our data raise the possibility that the supergroups might have evolved into distinct ecotypes within the same host species, potentially avoiding competition through niche partitioning and thereby achieving a stable co-existence.

Although niche partitioning has not yet been investigated for hosts infected with multiple *Wolbachia* strains, Veneti *et al.* [63] demonstrated that *Wolbachia* strains of different supergroups show distinct localization patterns within the host embryo. The A-group strains (with the exception of *w*Ri) were localized to the posterior part of the embryo, whereas the B-group strains were observed in the anterior part during the syncytial blastoderm stage. However, only a few highly similar strains of each supergroup were included in the analysis, and it remains to be determined whether the observed patterns are characteristic of a broader selection of strains from the two supergroups. Physical separation of endosymbionts within hosts does not necessarily have to be absolute to allow for speciation, since quantitative differences in associations with different habitats might also generate ecologically distinct species [6]. In analogy, differences in abundances and/or compartmentalization within the host could potentially lead to ecologically distinct species. Indeed, distinct localization patterns of endosymbionts within hosts have for example been observed for different genera of whiteflies [64].

While the current overlapping host ranges of supergroup A and B and the occurrence of multiple infections with strains from both groups appears to contradict the possibility of host specialization, several studies have provided some evidence for specialization to hosts and/or habitats [65]–[68]. However, these studies were based small gene datasets, such as the *wsp* gene that code for a hypervariable surface protein and/or core genes used in multi locus sequence typing. If these genes are as recombinogenic in all *Wolbachia* strains as reported here, sequence similarity measures within supergroups will reflect gene recombination histories rather than strain relationships. Correlations between genotypes and host-association patterns within supergroups will thus mostly depend more on the gene sets selected for the analyses.

In conclusion, both experimental evidence and additional genome data is needed in order to evaluate the ecological distinctness of *Wolbachia* strains both within and between supergroups. Now that a supergroup specific gene repertoire has been identified, it should be possible to investigate both the ecological roles of these genes, as well as the strain localization at various stages of host development.

### Are *Wolbachia* strains from super-groups A and B irreversibly separated?

All evidence gathered in this study indicates that strains from different supergroups represent distinct clusters, but are they irreversibly separated or do they still exchange genetic material? Importantly, our analyses have shown that recombination events between supergroup A and B have occurred, but that the fragments are of shorter sizes and have had a much lower impact on the genomes than recombination events within the groups. Recombination events that span over all or most of a gene are very rare since only 8 of the 660 gene trees did not provide support for the supergroup division. Consistently, we only identified a few long recombination tracts between the supergroups. These few transfers of co-located genes might thus exemplify how one organism can acquire another population's adaptation while the integrity of its own niche-defining characteristics is still preserved.

The *w*Ha and *w*No genomes are thought to have co-infected *D. simulans* for at least 200,000 years, which is a relatively short time period compared to at least a few million years since the divergence of the A and B-groups (as inferred from a few % difference in their 16S rRNA genes). Hence, even though we do not find more recombination between *w*Ha and *w*No than between other strains belonging to different supergroups, we cannot exclude the possibility that the exchange of genetic material between them would increase given longer time. Alternatively, there is some form of barrier to genetic exchange between strains of supergroup A and B.

The simplest form of barrier to gene transfer is the presence of incompatible mobile elements. However, we do not think that this is the case in *Wolbachia* since the gene phylogenies indicated transfer of phage genes across the supergroup boundary. Moreover, transfer of a complete bacteriophage genome between strains of different supergroups was recently discovered in *Nasonia vitripennis* [42]. Even so, there is no concrete evidence that these phages regularly transfer genetic material other than their own genomes, and thus there could still be differences in the frequencies at which genetic material is transferred between the two supergroups.

Another form of barrier is that the sequence divergence *per se* limits recombination. The mismatch repair system has been seen to prevent homologous recombination between divergent sequences and loss of the *mutSL* genes for the mismatch repair system is known to cause dramatic increases in both mutation and homologous recombination frequencies. However, even though we found that the *mutS* gene is full-length and probably functional in both the A and B-group *Wolbachia* strains, and that all genomes except the *w*Ri genome have two copies of the *mutL* gene, we found no inverse correlation between sequence divergence levels and recombination frequencies for individual genes. In natural populations, the *mutS* gene recombines and is gained and lost in a cyclic manner in response to environmental changes, leading to altered mutation and recombination rates. The resulting mutator phenotypes are selected during periods of environmental fluctuations and then restored by recombination with a functional copy from another strain [69]. We saw that in *Wolbachia*, one of the *mutL* genes is associated with a prophage element in *w*Pip and *w*Ha and located near to a previously detected insertion in *w*Mel that might stem from a phage, indicative of horizontal gene transfer. Additionally, we detected intra-genic recombination in both the *mutS* and *mutL* genes. Thus, the presence of a seemingly functional mismatch repair system all strains analyzed does not preclude that recombination frequencies could have fluctuated in the past due to gains and losses of these genes.

A recent model suggests that almost identical sequences between the donor and recipient are required at one or both ends of a recombination fragment in order for recombination to occur and that the imported fragments are digested until a good enough match is obtained [70]. Consistent with our data, this model predicts that shorter recombination tracts will be found between more divergent sequences, since more cuts are required in order for the ends to match. Essentially, if true, this implies that when two genomes have diverged enough only short fragments can recombine between them. As a consequence, it is unlikely that recombination events are sufficient to invoke convergence between the supergroups even though they share the same habitat for a long period of time, as is the case with the *Wolbachia* strains *w*Ha and *w*No. Although we did not see a correlation between sequence diversity and intragenic recombination, this model cannot be ruled out since the end points of each recombination fragment were not investigated.

Genome rearrangements present yet another barrier to recombination and is thereby an important factor in speciation processes in eukaryotic organisms, mainly because of suppressed recombination at rearranged sites during meiosis in heterozygous individuals [71]. Although bacteria do not evolve by sexual reproduction, homologous recombination could be suppressed in chromosomal regions that are not co-linear because of rearrangements or insertions of genes in one of the two genomes. Indeed, a recent study showed that recombination frequencies are suppressed close to lineage-specific genes, which might lead to higher divergence levels in their vicinity [72]. Furthermore, long recombination events can only occur if the target genome has a similar gene order. Since a single long recombination event can override several shorter intra-genic recombination fragments, extensive rearrangements could contribute to the separation of the lineages. The genomes of *Wolbachia* strains that belong to the same supergroup show much higher colinearity than strains of different supergroups, potentially contributing to the observed lower frequency of recombination events between the A and B supergroups.

In summary, a number of different explanations could account for the observed reduced level of recombination between supergroup A and B. Although we do not know whether there has been selection against recombination between supergroups or if the reduced levels of recombination was driven by neutral processes alone, our results strongly suggest that the A and B supergroups have now become irreversibly separated.

### What started the speciation process in *Wolbachia*?

The acquisition of advantageous novel genes or mutations is hypothesized to trigger speciation events according to the ecotype model of speciation, which has so far only been evaluated for free-living bacteria [73]. In this context, it is notable that we have identified supergroup-specific genes sets that appear to be the result of horizontal gene transfers. Although it is too early to speculate about the functions of these group-specific genes, it is quite possible that their acquisitions induced significant phenotypic changes. Selective advantages associated with any of these phenotypes could have purged diversity within the groups, thereby contributing to the genetic separation of the two lineages.

Another scenario could be that the loss or gain of genes in one strain of *Wolbachia* resulted in reproductive isolation between infected hosts, for example through CI [74], [75]. However, it is difficult to evaluate the likelihood for such a scenario, since multiple infections and recent horizontal transmission of *Wolbachia* strains between different host-species have blurred the ancestral patterns of infections.

Alternatively, the speciation event may have been triggered or enhanced by extensive rearrangements, due to a burst in the activity of IS-elements. All *Wolbachia* genomes from supergroup A and B sequenced to date contain an unusually high level of IS-elements. For example, 11% of the genome of *Wolbachia* strain *w*Ri was estimated to consist of IS-elements, and 17 of the 35 identified breakpoints between the genomes of *w*Mel and *w*Ri are located at IS-elements [38]. Additionally, many of the IS elements in *Wolbachia* genomes carry mutations that are likely to have rendered these elements non-functional, which is an unusual feature of bacterial IS-elements since they are commonly believed to have a rapid turnover rate within genomes [76]. Making use of the presence of these degraded IS-elements, a recently published simulation study aiming to explain the distribution of IS copies in the modern *Wolbachia* genomes suggested two major periods of intense transpositional activity, a very recent burst and an ancient expansion of the most divergent IS copies [77]. Such an expansion could have induced major changes in gene order structures, leading to suppressed recombination close to the breakpoints. Since two rearranged genomes can never converge to the same gene orders again, an ancestral expansion of IS-elements followed by genome rearrangements could have irreversibly separated the two groups. However, since the age of the ancestral expansion is not known, it is difficult to test this hypothesis. The recent expansion of IS-elements in *Wolbachia* could potentially have lead to similar diversifications in more closely related strains, a hypothesis that could be tested by investigating diverse lineages within the same supergroups.

It is obvious that no single speciation hypothesis will be applicable to all bacteria. Although *Wolbachia* is an obligate intracellular bacterium, it is atypical in that it is a generalist with a high prevalence and a broad host range in a diverse group of insects. The most remarkable aspect of its evolution is the expansion of the host range, which might have occurred independently in both supergroups after their separation. The acquisition of genes to manipulate the host combined with high recombination frequencies to shuffle beneficial alleles among all members in the group could help explain much of this ability. Many questions remain to be solved, such as for example if there is adaptive selection for ecological divergence within supergroups, and if strains from different supergroups inhabit different niches within their broad range of host species. To further investigate speciation processes in *Wolbachia*, we need to study the global distribution patterns and population structures of hosts and endosymbionts. The methods developed in this paper offer the possibility to perform such large-scale, whole-genome surveys of *Wolbachia* and other endosymbionts.

## Methods

### DNA preparation and sequencing

The *w*No-infected fly line was generated by a series of backcrosses on a double-infected fly line collected on Noumea in 1989 [78]. The *w*Ha-infected fly line was collected on Hawaii in 1990, as a natural single-infection [79]. Both *Wolbachia*-infected fly lines have been kept at the laboratory of Prof. Kostas Bourtzis for over fifteen years and have extensively been used in *Wolbachia*-related experimental work.

The purification of *Wolbachia* cells was carried out as in [39], with some modifications. Flies were allowed to oviposit on apple-juice agar for two hours, and 15–30 embryos were collected for the purification. The embryos were dechorionated in bleach, rinsed with water, and homogenized in phosphate-buffered saline (PBS) buffer with a sterile micropestle. The homogenate was centrifuged at 400 *x* g for 5 min to pellet large debris, including host nuclei. The supernatant was centrifuged at 5,400 *x* g for 5 min to pellet *Wolbachia* cells. The pellet was re-suspended in PBS, and another slow centrifugation was carried out (400 *x* g for 5 min) to remove remaining debris. The supernatant was passed first through a 5 µm pore size filter (Millipore, Bedford, MA), and then through a 2.7 µm pore size filter (Whatman, USA). The filtrate was centrifuged at 6,900 *x* g for 15 min to pellet the *Wolbachia* cells. Most of the supernatant was removed, leaving a bacterial pellet in approximately 3–5 µl PBS.

A multiple-displacement amplification (MDA) was carried out directly on the bacterial pellet, using Repli-g midi kit (Qiagen) according to manufacturer's instructions (protocol for Amplification of Genomic DNA from Blood or Cells). The amplified samples were cleaned prior to sequencing with QIAamp DNA mini kit, according to manufacturer's instructions (Qiagen, supplementary protocol for Purification of REPLI-g amplified DNA). Since MDA is known to be extremely sensitive, precautions were taken to avoid contamination during the purification of *Wolbachia* cells, including sterile-filtering of all solutions, and autoclaving/UV-treatment of plastic utensils.

Three independently amplified samples for each *Wolbachia* strain were used for library construction and sequencing, so that each genome was sequenced by ½ plate of single-end and 3 kb paired-end 454 and 1/12 lane paired-end Illumina. 454 sequencing was done at SciLifeLab Stockholm on a 454 Roche FLX machine using Titanium chemistry and standard preparations for single-end and 3 kb paired-end libraries. Illumina sequencing was done on a HiSeq2000 instrument at the Uppsala SNP & SEQ platform, using standard Illumina protocols for preparation of paired-end libraries, generating 2×100 bp sequences from each fragment.

### Genome assembly and annotation

The 454 datasets were assembled *de novo* with both Newbler (454 Life Sciences Corp., Roche, Branford, CT 06405, US) and Mira [80]. Assemblies were compared with Mauve [81] and ACT [82] and the discrepancies between the best assemblies and all sequence gaps were resolved with PCR amplification from total fly DNA extractions (DNeasy Blood and Tissue kit, Qiagen) and subsequent direct sequencing of the PCR products. Since the Newbler assembly proved to be generally more correct it was used as a reference to order the contig sequences from MIRA into scaffolds and close the remaining gaps, resulting in two circular *Wolbachia* genomes. In two positions on the *w*Ha genome PCR-products could not be obtained, but read-pairs that go in and out of the repeat sequence associated with these genome positions support the current arrangement. Gap closure and manual sequence editing of PCR products was done using Consed [83]. Consed was also used to map the Illumina sequences onto the contigs generated using 454 data, in order to correct errors in homopolymer tracts.

To evaluate the purity and quality of the DNA samples used for sequencing, the sequence reads were mapped onto the completed genomes. The Illumina reads were filtered using Trimmomatic [84], and mapped using bwa [85]. The sam-formatted output file from bwa was converted to bam, sorted in coordinates and duplicated reads were marked using Picard tools (http://picard.sourceforge.net). Proper and non-proper read pairs (as set in the sam-file flag by bwa) were extracted with samtools [86]. The single and paired-end 454 reads were mapped separately using the Newbler mapper. For the paired-end 454 reads, true and false pairs (as defined in the output file 454PairStatus.txt by Newbler) were extracted and mapped separately. Coverage was calculated from the bam-files using the depth command in samtools and subsequently plotted using R (R development core team 2011).. The mean quality of assembled and non-assembled 454 reads was plotted with Prinseq [87].

An annotation pipeline was developed using the Diya framework [88]. Prodigal was used for gene prediction [89], GenePrimp for identifying suspicious start/stop codons and pseudogenes [90], and hmmsearch as implemented in pfam_scan.pl was used for domain prediction with the PFAM database [91]. All annotations were manually edited using Artemis [92]. Overview figures of similarity between complete genomes and local genome regions were generated with GenoPlotR [93].

IS-elements were identified based on open-reading frames and a manual search of all repeats. All IS-elements were assigned to an IS family by TBlastX searches against IS-finder [94]. Functional IS-elements were defined as alignments that could be extended to contain the complete annotated IS-element. IS-elements that were truncated compared to their best hit in IS-finder or contained frameshifts were considered non-functional.

### Phylogenomics

Homologous genes between six *Wolbachia* strains (wHa, wNo, wRi, wMel, wPip and wAlbB) were determined using reciprocal protein blast searches between all the protein sequences from the genomes and subsequent clustering with the MCL algorithm [95]. In order for genes to be considered homologous, the shortest protein in a pair needed to be at least 60% of the length of the longer gene and be aligned over at least 80% of its length.

Ortholog clusters containing a single gene from all 6 *Wolbachia* genomes were aligned on the protein level using mafft [96] and backtranslated to nucleotides. The alignments were pruned to remove gap sites present in 50% or more of the aligned sequences.

A strain phylogeny was inferred on a concatenate alignment of the single gene orthologs in RAxML using the GTRGAMMA model and constructing 1 slow best maximum likelihood tree and 1000 rapid bootstrap replicates. Additionally, phylogenetic trees were inferred independently for each ortholog cluster by RAxML [97] using the GTRCAT model, and constructing 1 slow best maximum likelihood tree and 100 rapid bootstrap replicates. Pairwise Robinson-Fould (R-F) distances were calculated using RAxML by inputting a concatenated file with the 660 individual gene trees. The weighted R-F distances were used to cluster the trees with hclust (method complete and height cutoff of 1) in R. Phylogenetic trees of clusters with members of all strains, but containing paralogous copies and located in prophage regions were inferred by the same method as the single gene orthologs. However, since the current assembly of the *w*AlbB genome does not contain complete genes for most of the prophage, this strain was excluded from the analysis.

### Substitution frequencies

The same 660 single-gene ortholog clusters were used to calculate synonymous substitution rates (dS) between all pairs of genes in the alignment using codeml from the PAML package with the codon-based model of substitutions described in [98] and nucleotide distances with RAxML using the GTR model. The pair-wise dS-values obtained were used to quantify the amount of recombination within supergroup A and B by plotting relative dS- values in a ternary plot and calculating the spread of the values from the mean relative dS-values by using R, as described in [38].

### Mutation and recombination

The alignments of the 660 single-gene ortholog clusters were used for recombination detection within genes with PhiPack [99] (which calculates the p-values for three individual methods, Neighbour similarity score (NSS), Maxchi and Phi) and GENECONV [100]. Recombination was inferred for p-values less than 0.01. For counting recombination between vs. within supergroups with geneconv, only global inner fragments with a Bonferroni corrected KA p-value less than 0.05 was used. Additionally, to calculate the r/m parameter, two independent ClonalFrame [101] runs were performed on a concatenated alignment of all the single orthologs as individual blocks using 100.000 iterations, with a burn-in of 50.000 iterations and recording the parameters every 100^th^ iteration. Convergence between the clonal-frame runs was tested using the ClonalFrame graphical user interface. r/m for each node of the tree was calculated from the output file of the two separate ClonalFrame runs. The probability of a substitution generated by mutation was calculated as (1-R)*S and the probability of a substitution being generated by recombination was calculated as R*S, where R is the posterior probability of recombination and S is the posterior probability of substitution. Only positions where the probability of substitution via mutation or recombination was higher or equal to 0.95 were counted. The number of recombination events was calculated by looking at continuous stretches of sites were the posterior probability of recombination was never lower than 0.5 and contained at least one site with a probability of 0.95.

Geneconv was run using three different levels of mismatch penalty, in order to account for differences in divergence between the strains and differences in age of the transferred fragments. The mismatch penalty is inversely proportional to the total number of site differences between two sequences, and directly proportional to the gscale parameter (except when no mismatches are allowed, gscale = 0) according to the formula; mismatch penalty = (number of total polymorphisms in the alignment)* gscale/(number of site differences between each pair of sequences). This means that sequences with a lower number of total differences, will get a higher penalty for a mismatch with the same gscale setting.

### Analyses of supergroup specific genes

MCL clusters that contained genes from only super-group A or B were further analyzed by taking the protein sequences from either *w*Ha (representing the A supergroup) and *w*No (representing the B supergroup) and blasting (tblastn) them against the complete genomes from the other supergroups, including the supergroup D genome of *Wolbachia* wBm. Clusters that did not have a match in any of the other genomes with either an e-vale less than e-5 and 60% of the protein aligned or an e-value less than e-20 and 30% of the protein aligned and identity of minimum 35%, were considered supergroup specific. Additionally, if the matches from tblastn contained stop codon or frame-shifts, the hit was called a pseudogene even if the above criteria were met.

The protein sequences of *fic* domain proteins with known function (FiDo family) as listed in [102] were downloaded from Genbank. Additionally, the protein sequences for the 10 best non-overlapping blastp hits against the nr database when using the three *Wolbachia* fic genes were downloaded. Similarly, for *cydA, cydB, argR* and the arginine ABC transporter genes, the top 50 blastp hits against the nr database were downloaded. For the outer membrane proteins specifically found in the B-supergroup, no additional species were found in the database, but the homologous protein sequences from *w*VitB were included. In all cases, the protein sequences were aligned with mafft and pruned to remove gap sites that were present in 50% or more of the aligned sequences. The phylogenetic trees were inferred with RAxML using the PROTCATWAG model, and constructing 1 slow best maximum likelihood tree and 1000 rapid bootstrap replicates.

### Data deposition

The complete sequences of *Wolbachia w*No and *w*Ha genomes are deposited in Genbank under accession numbers CP003883 and CP003884, respectively.

## Supporting Information

Figure S1 Quality scores for the wHa and wNo genomes. The histograms show the mean phred-based quality score per sequence of the 454 sequencing data, for wHa (A–D) and wNo (E–H). Separate plots were made for paired-end sequences assembling (A, E), and not assembling (B,F), and for single-end reads assembling (C,G) and not assembling (D,H).
(PDF)

Figure S2 Sequencing coverage for the *w*Ha genome. The coverage is shown separately for each data-set when mapped against the complete genome of *w*Ha. Each data-set was generated with separate amplification reactions. A) 454 single-end reads, B) 454 paired-end reads, C) Illumina paired-end reads and D) *w*Ha reads found in the *w*No illumina paired-end sequences. Grey crosses indicate mean read coverage per 100 bp. Blue lines indicate the read coverage smoothed with the Savitzky-Golay algorithm, with variable window sizes.
(PDF)

Figure S3 Coverage of chimeric and non-chimeric sequences reads. The coverage for each data-set of Illumina and 454-paired end, for proper and non-proper pairs in the *w*Ha and *w*No genomes (A, C, E, G) (for definition see materials and methods) and the correlation between the two (B, D, F, H). Proper pairs are plotted in blue and non-proper pairs are plotted in red. A and B) wHa Illumina paired-end reads, C and D) wHa 454 paired-end reads, E and F) wNo Illumina paired-end reads, G and H) wNo 454 paired-end reads.
(PDF)

Figure S4 Example trees from the nine major clusters found for single-gene phylogenies. Four phylogenetic trees from each cluster are shown, where the different clusters are represented by the letters from A to I. Numbers on the nodes show the support from 100 bootstrap replicates.
(PDF)

Figure S5 Overview of prophages from completed *Wolbachia* genomes. Blue arrows indicate annotated genes. Grey lines of different intensity indicate the similarity between sequences.
(PDF)

Figure S6 Histograms of recombining fragments predicted by GENECONV. The plots show the size distribution of recombining fragments, Top row; gscale = 0, Middle row; gscale = 3 and Bottom row; gscale = 1. A, D, G) between pairs of supergroup A and B strains; B, E, H) between pairs of supergroup A strains and C, F, I) between pairs of supergroup B strains.
(PDF)

Figure S7 COG functional categories for genes found to have recombined between super-groups, as compared to non-recombined genes. The figure shows the number of genes in COG catergories, where blue bars represent the total core gene set, and green bars represent genes found to have been recombined between supergroup A and B strains. Non-standard COG categories are X = genes where hits has the designation NO_COG, XX = genes with less than 2 hits against the COG database and XY = genes where the two first hits fall into different COG categories.
(PDF)

Figure S8 Protein clusters found in combinations of three *Wolbachia* strains. The bars show the number of protein cluster found to be shared for each three-strain combination; the A and B indicate the strain combinations of the A-supergroup and B-supergroup, respectively.
(PDF)

Figure S9 Phylogenetic analyses of the *fic*-domain proteins. The tree includes blast-identified homologues of the *Wolbachia fic*-domain protein genes, and members of the “FiDo” family (see methods). Highlighted genes: Green - *Wolbachia fic1*, Blue - *Wolbachia fic2*, Light yellow – *Wolbachia fic3*, Lilac – The HYPE subgroup, including human HYPE gene, Dark yellow – The Fic sub-group, including the *Bartonella* effector proteins, Grey- The Doc sub-group. Numbers on the nodes represent the support from 1000 bootstrap replicates.
(PDF)

Figure S10 Gene map of the *murC* regions in supergroup A and B strains. Blue arrows indicate annotated genes and the black box of the genes in *w*No and *w*Pip mark the position of the recombinase beta zinc ribbon domain. Grey lines of different intensity indicate the similarity between sequences.
(PDF)

Figure S11 Outer membrane proteins specific for the B group strains. A) Gene map of the outer membrane protein region in B groups strains compared to A group strains. Colored arrows indicate the outer membrane proteins in the B-group genomes. Grey lines of different intensity indicate the similarity between sequences. B) Maximum likelihood trees of the B-group specific outer membrane protein. The colored blocks follow the coloring scheme in Figure 9A, showing the grouping of orthologs between genomes. Numbers on the nodes represent the support from 100 bootstrap replicates.
(PDF)

Table S1 Sequencing data and assembly. Number of reads and read length statistics for 454 sequencing data, shown separately for assembling and non-assembling reads (using the Mira assembler).
(DOCX)

Table S2 IS elements. Number of identified putative functional and non-functional IS-elements for each of the genomes *w*Ha and *w*No. Sequences were assigned to IS-families based by TBlastX searches on IS-finder (https://www-is.biotoul.fr). An IS-element was considered to be functional if the query sequence could be aligned to the complete annotated IS-element. IS-elements were considered non-functional if the alignment was partial or contained frame-shifts.
(DOCX)

Table S3 Pairwise non-synonymous (dN) and synonymous (dS) substitution frequencies. The upper right section of the table shows the pairwise dN-values, whereas the bottom left section shows the pairwise dS-values between strains.
(DOCX)

Table S4 Summary of GENECONV results for intragenic recombination events with different gscale settings. GENECONV was tested on all 660 gene single-gene orthologs present in all supergroup A (*w*Ha, *w*Ri, *w*Mel) and B (*w*No, *w*Pip, *w*Alb) with three different gscale settings (see materials and methods). Only gene alignments with support for recombination with at least two additional methods were evaluated.
(DOCX)

Table S5 A-group specific genes. All genes found to be present in all supergroup A strains, while being absent from all supergroup B and D strains are listed with locus tag numbers. For those genes where a pseudogenized homolog could be identified in either supergroup B or D, “pos.” indicates that the homolog is present in a region of synteny between the genomes, whereas “Not pos.” indicates no synteny around the detected homolog. If a homolog could be identified in the *Anaplasmataceae* family, the members containing the gene are noted: Ac, *Anaplasma centrale*, Am, *Anaplasma marginale*, Eca, *Ehrlichia canis*, Ech., *Ehrlichia chaggeensis*. When applicable, the best significant blast hit outside the *Wolbachia* group is indicated.
(DOCX)

Table S6 B-group specific genes. All genes found to be present in all supergroup B strains, while being absent from all supergroup A and D strains are listed with locus tag numbers. For those genes where a pseudogenized homolog could be identified in either supergroup B or D, “pos.” indicates that the homolog is present in a region of synteny between the genomes, whereas “Not pos.” indicates no synteny around the detected homolog. If a homolog could be identified in the *Anaplasmataceae* family, the members containing the gene are noted: Ac, *Anaplasma centrale*, Am, *Anaplasma marginale*, Eca, *Ehrlichia canis*, Ech., *Ehrlichia chaggeensis*. When applicable, the best significant blast hit outside the *Wolbachia* group is indicated. Note that the *murC* gene in supergroup A and D is a complete version.
(DOCX)
