# Supplementary figures and images for: Comparative Genomics of Wolbachia and the Bacterial Species Concept
Source: PLoS Genet. 2013 Apr 4;9(4):e1003381. doi: 10.1371/journal.pgen.1003381 (PMC3616963; doi:10.1371/journal.pgen.1003381)

A

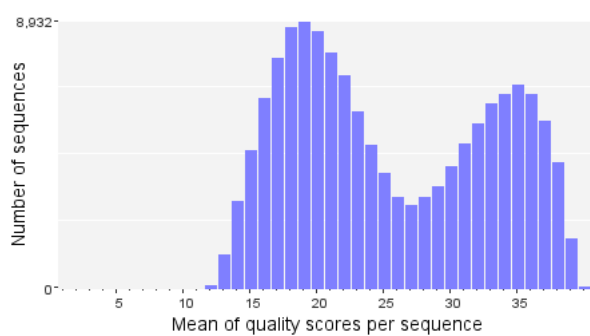

B

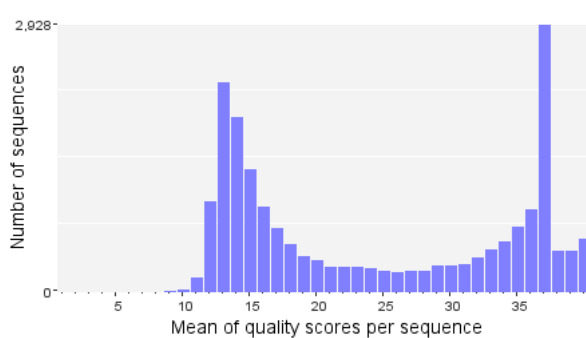

C

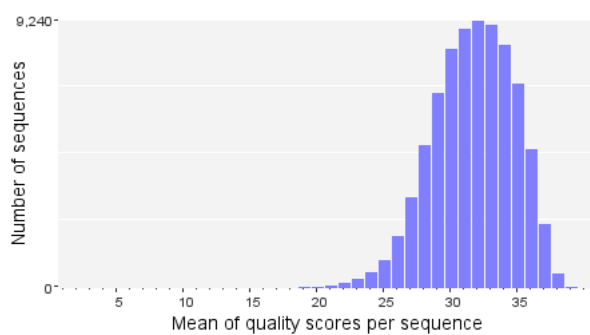

D

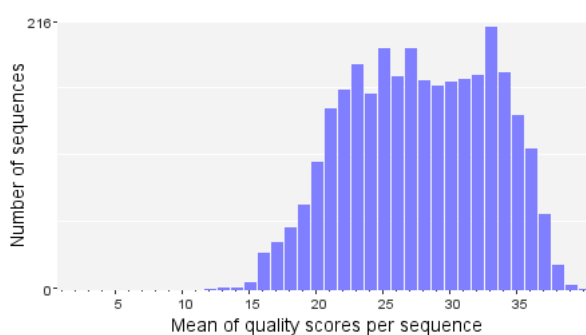

E

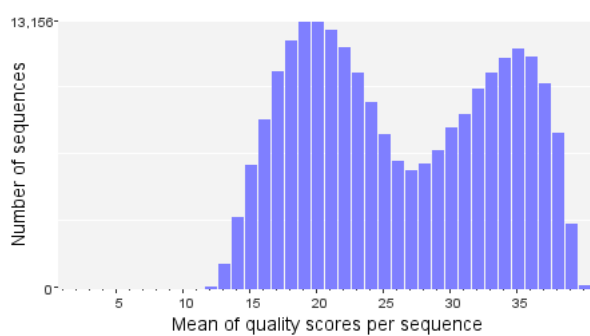

F

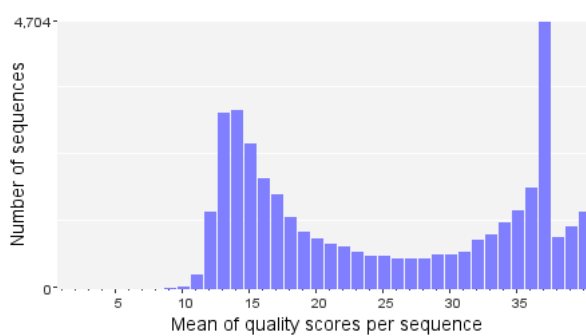

G

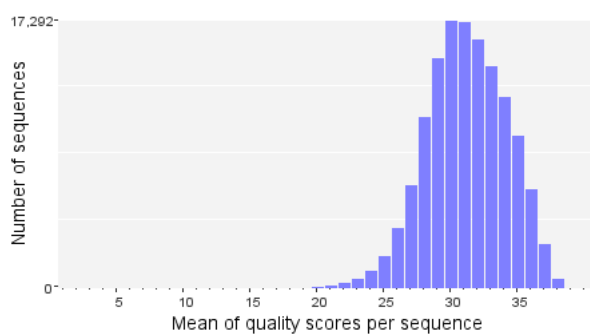

H

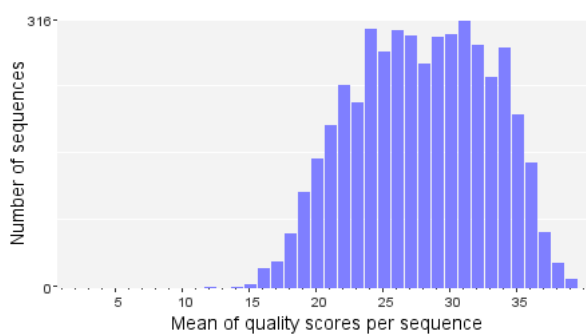

Supplement: Figure S1 — Quality scores for the wHa and wNo genomes. The histograms show the mean phred-based quality score per sequence of the 454 sequencing data, for wHa (A–D) and wNo (E–H). Separate plots were made for paired-end sequences assembling (A, E), and not assembling (B,F), and for single-end reads assembling (C,G) and not assembling (D,H). (PDF) [file pgen.1003381.s001.pdf]

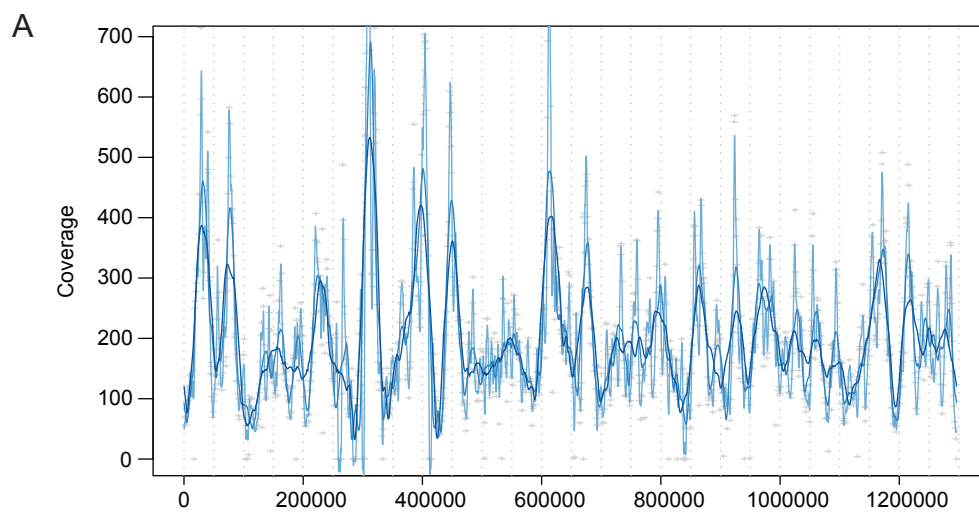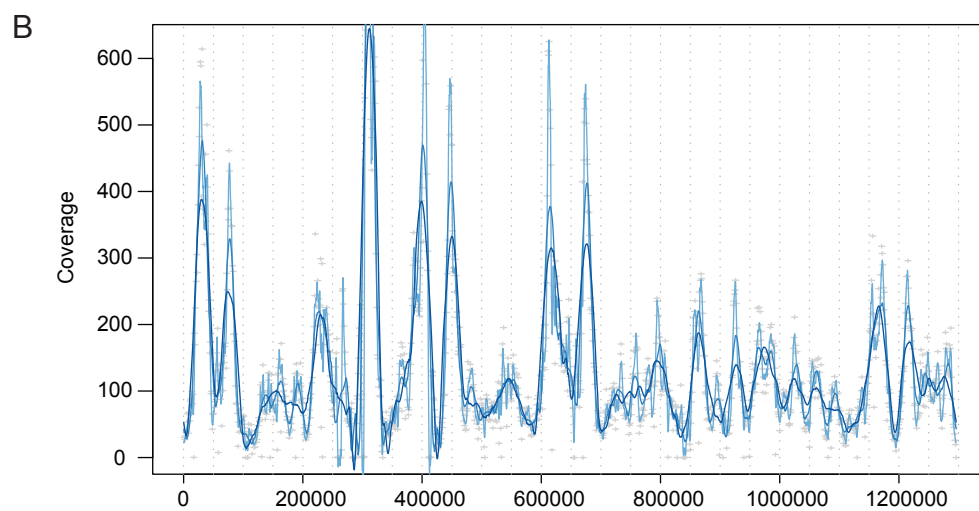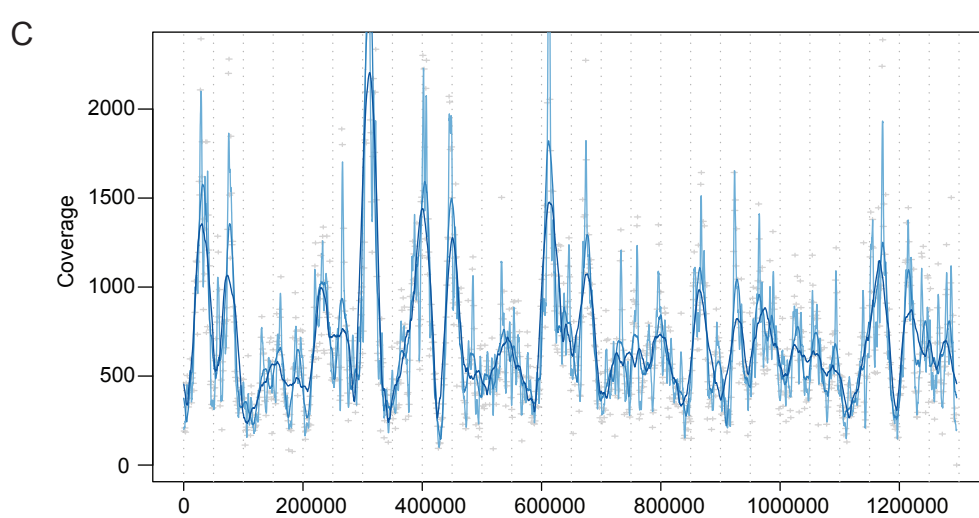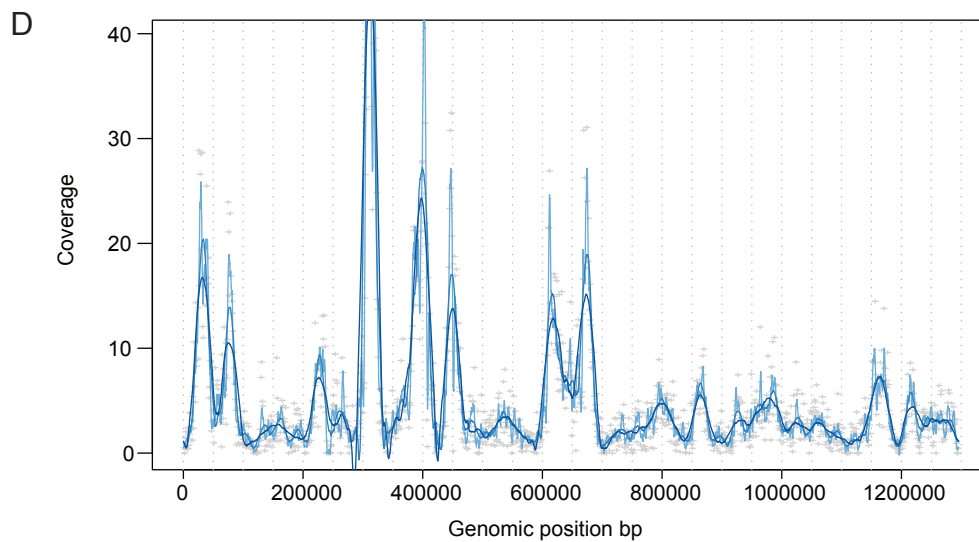

Supplement: Figure S2 — Sequencing coverage for the wHa genome. The coverage is shown separately for each data-set when mapped against the complete genome of wHa. Each data-set was generated with separate amplification reactions. A) 454 single-end reads, B) 454 paired-end reads, C) Illumina paired-end reads and D) wHa reads found in the wNo illumina paired-end sequences. Grey crosses indicate mean read coverage per 100 bp. Blue lines indicate the read coverage smoothed with the Savitzky-Golay algorithm, with variable window sizes. (PDF) [file pgen.1003381.s002.pdf]

A

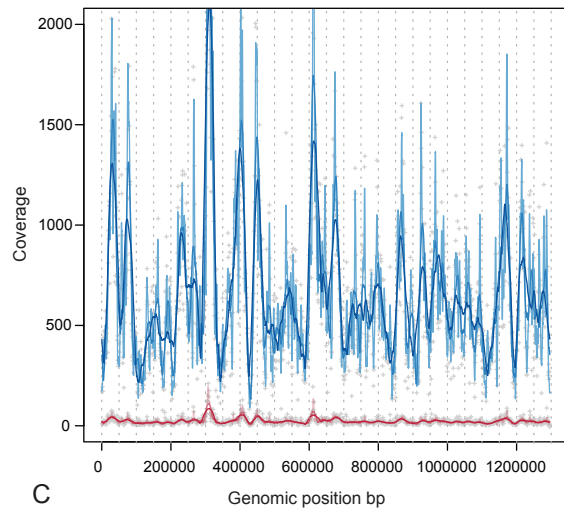

B

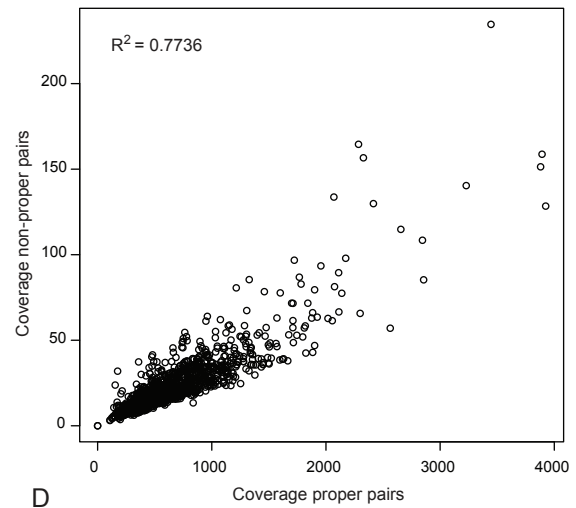

C

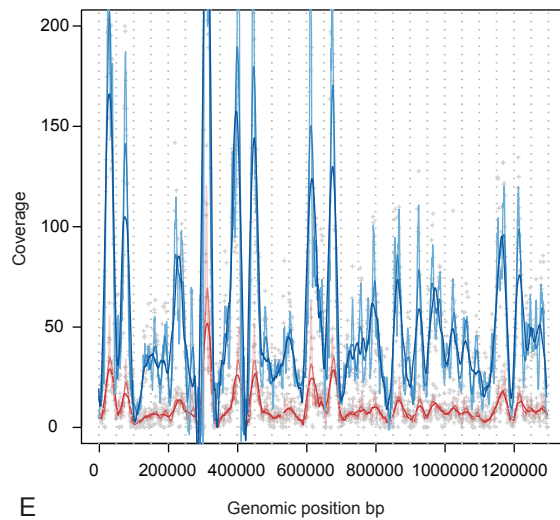

D

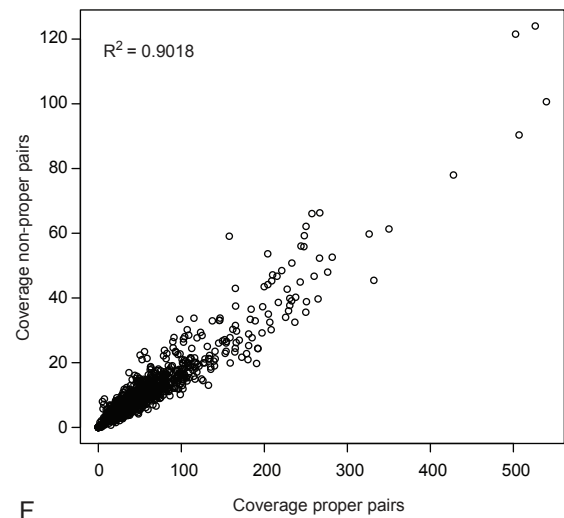

E

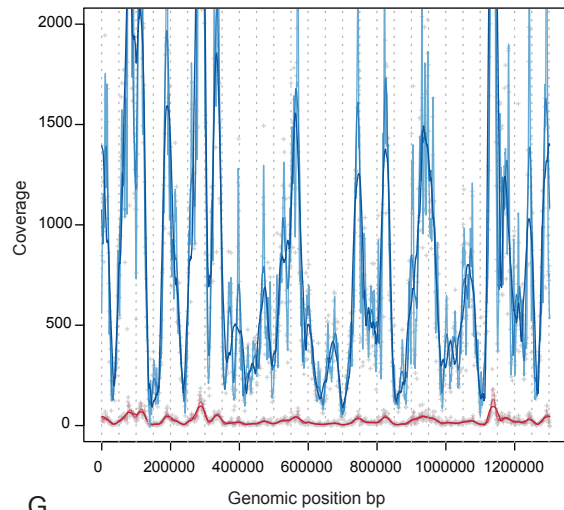

F

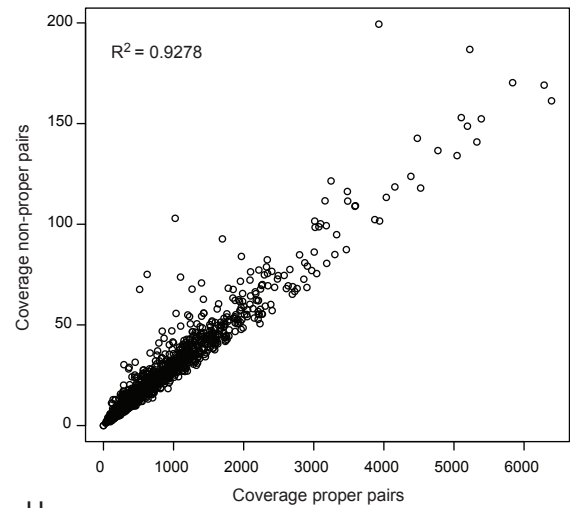

G

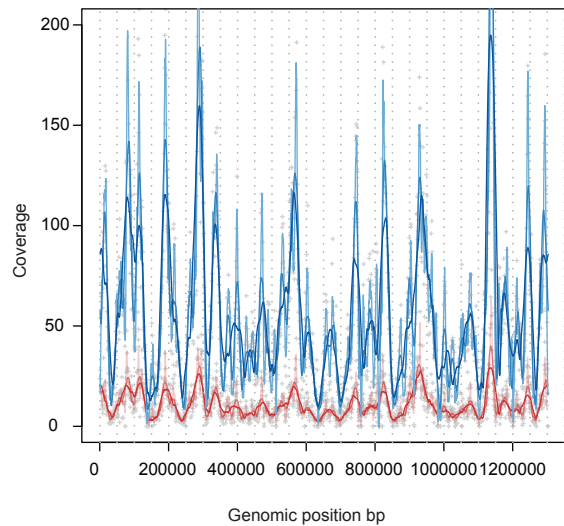

H

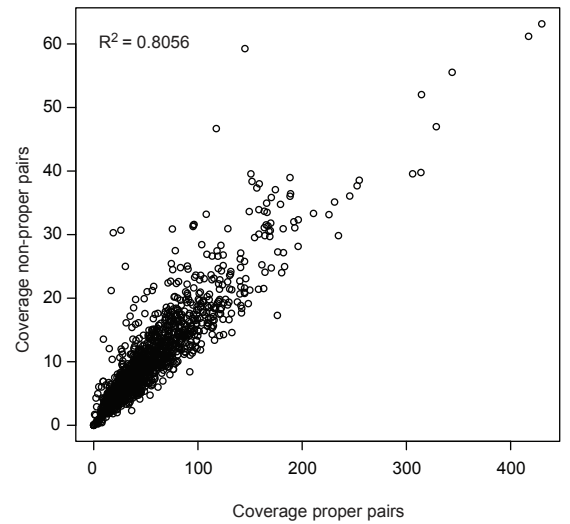

Supplement: Figure S3 — Coverage of chimeric and non-chimeric sequences reads. The coverage for each data-set of Illumina and 454-paired end, for proper and non-proper pairs in the wHa and wNo genomes (A, C, E, G) (for definition see materials and methods) and the correlation between the two (B, D, F, H). Proper pairs are plotted in blue and non-proper pairs are plotted in red. A and B) wHa Illumina paired-end reads, C and D) wHa 454 paired-end reads, E and F) wNo Illumina paired-end reads, G and H) wNo 454 paired-end reads. (PDF) [file pgen.1003381.s003.pdf]

A

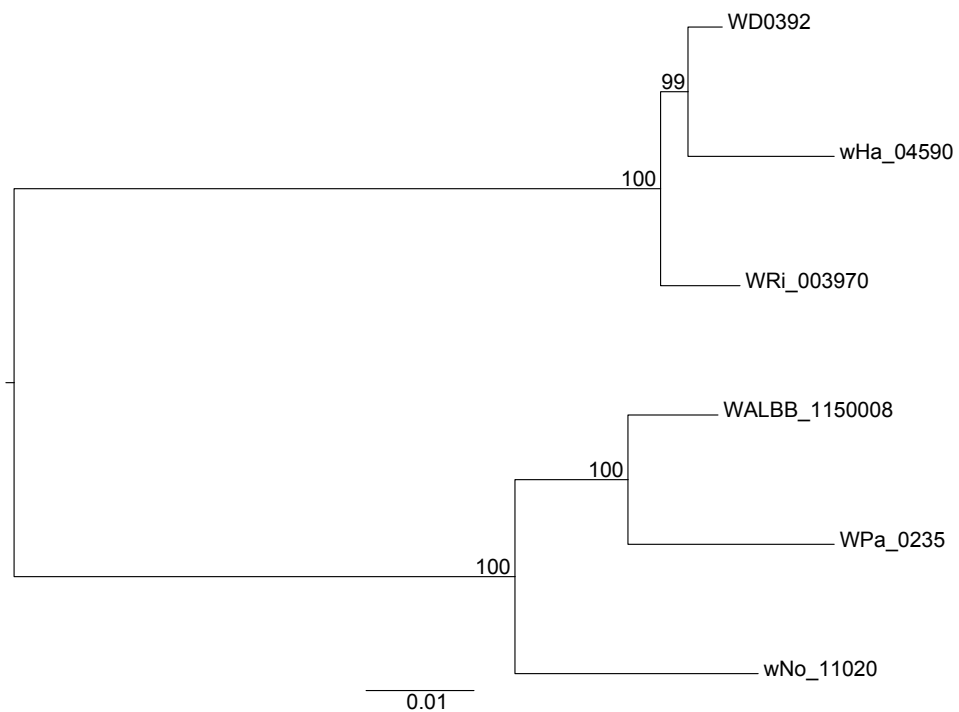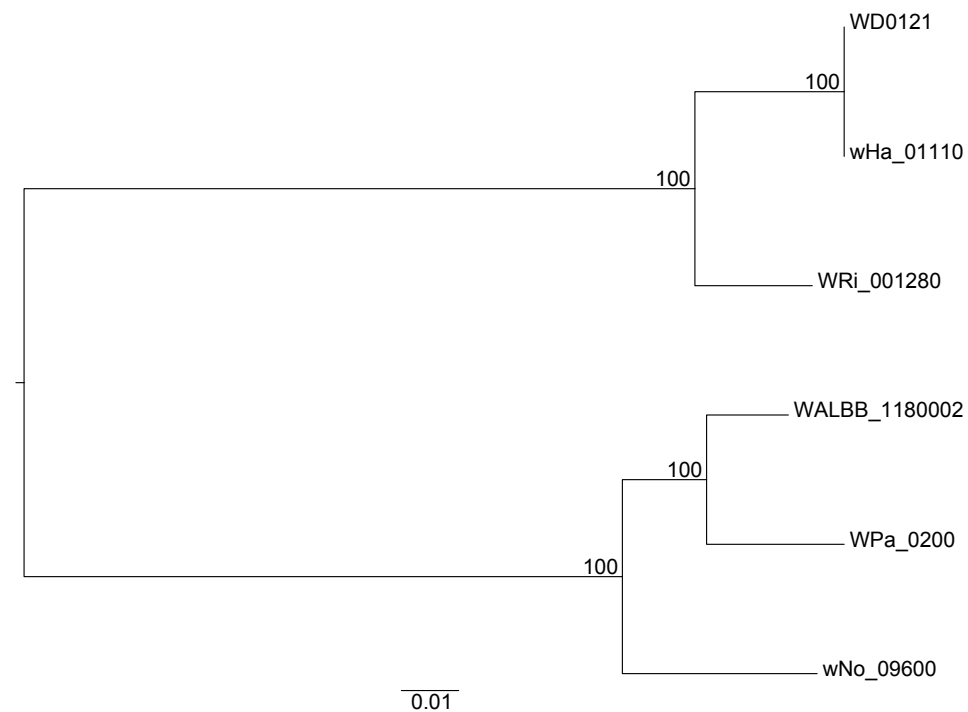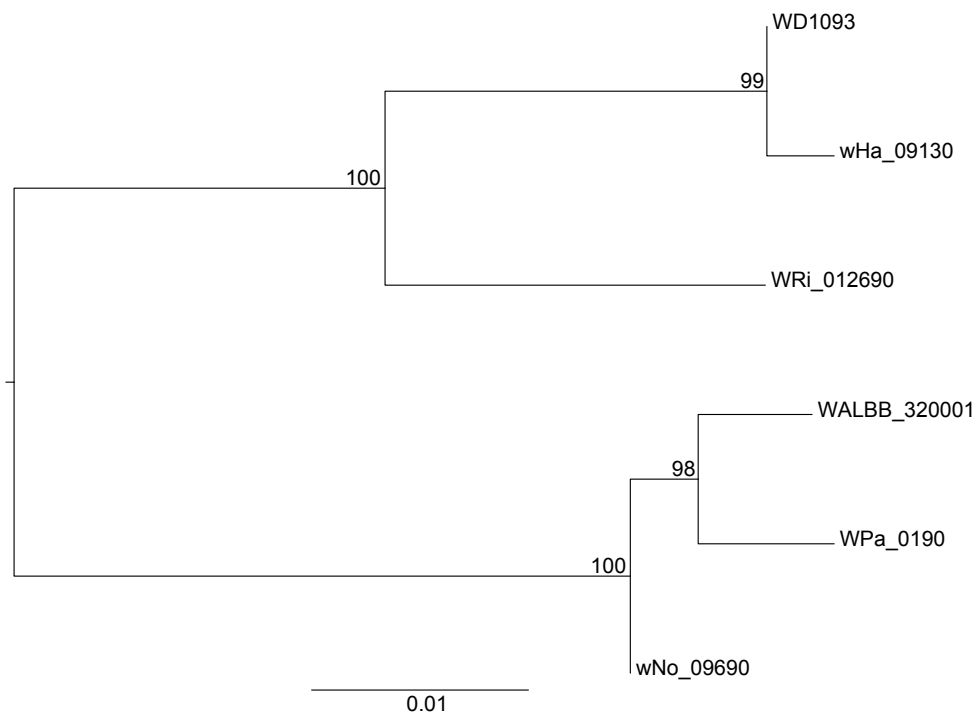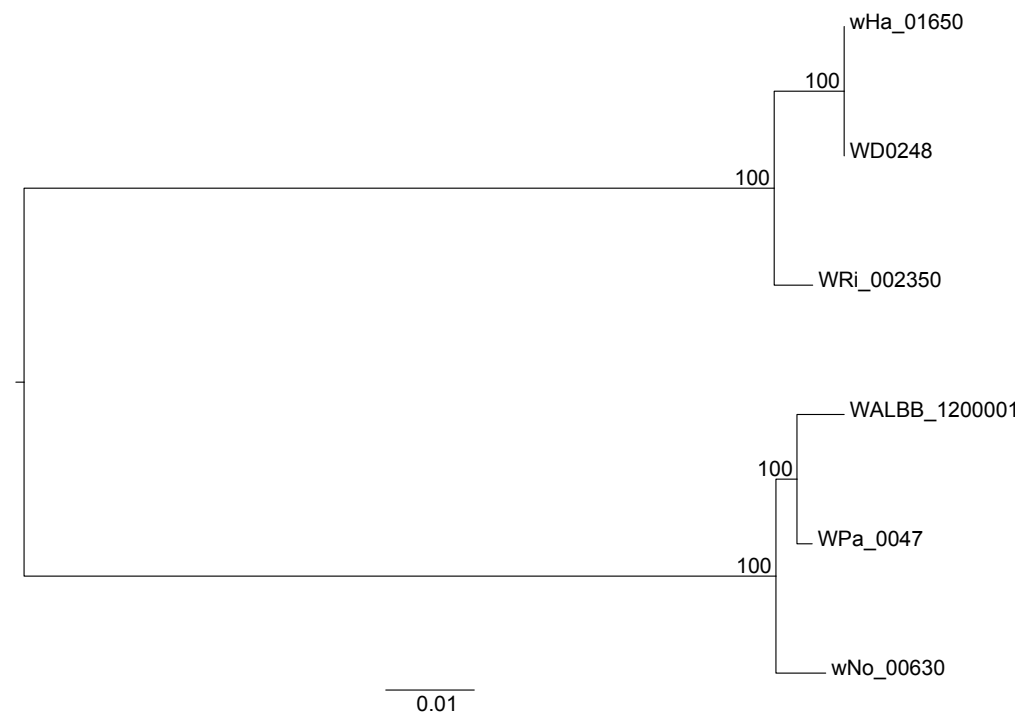

B

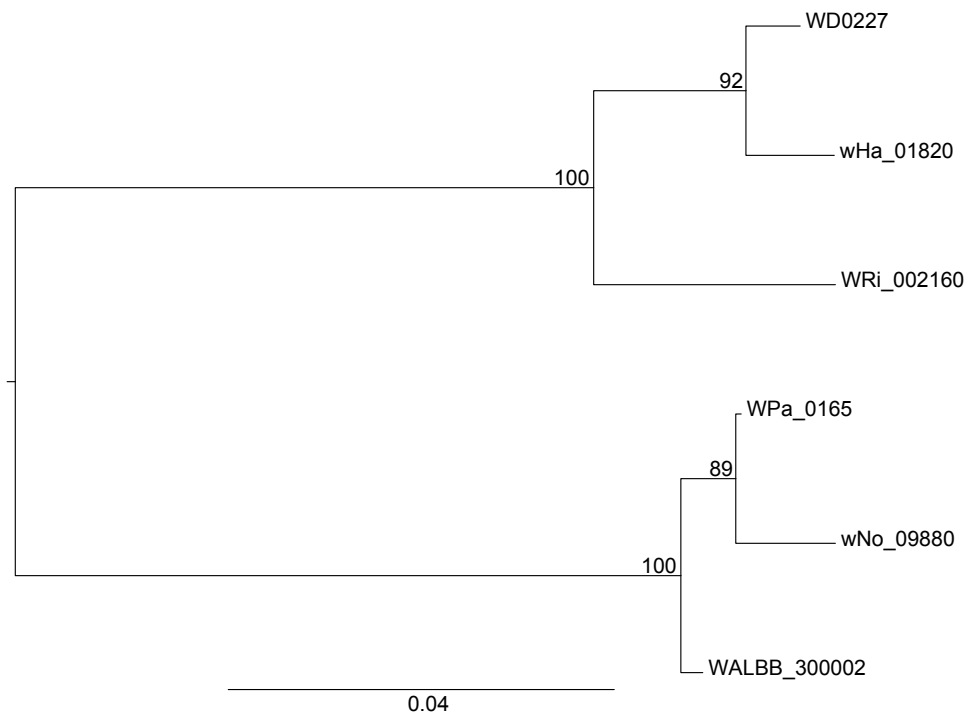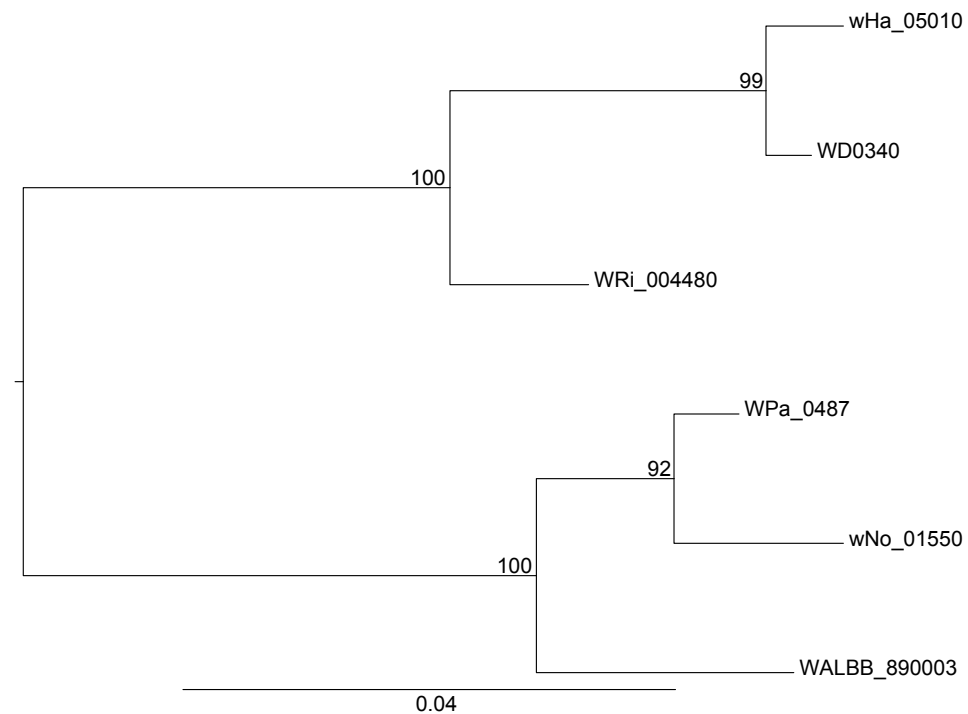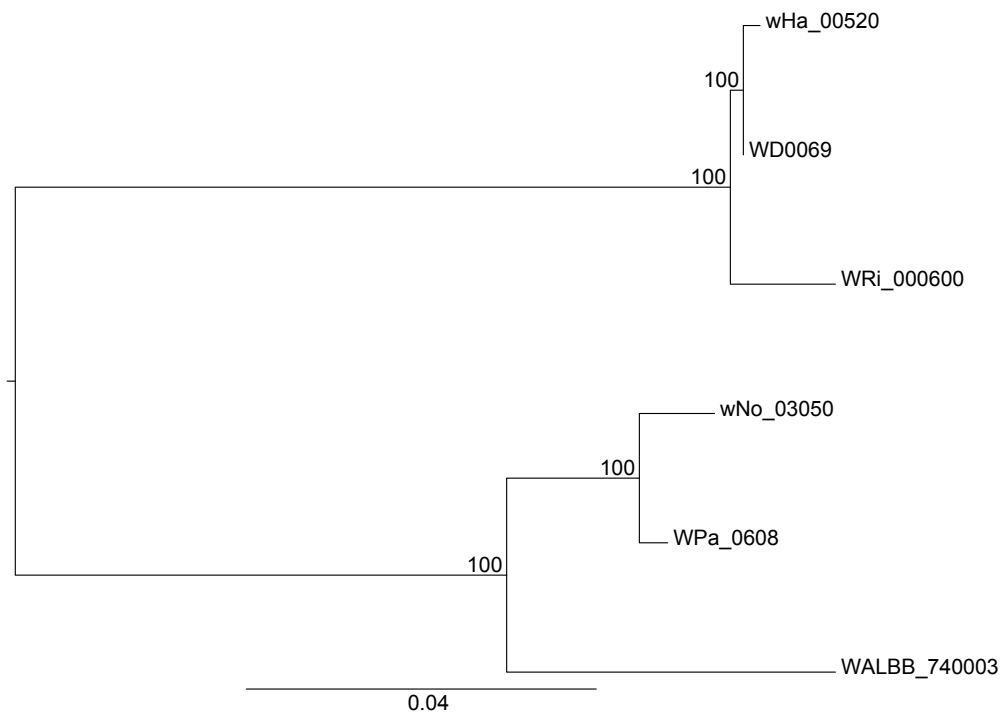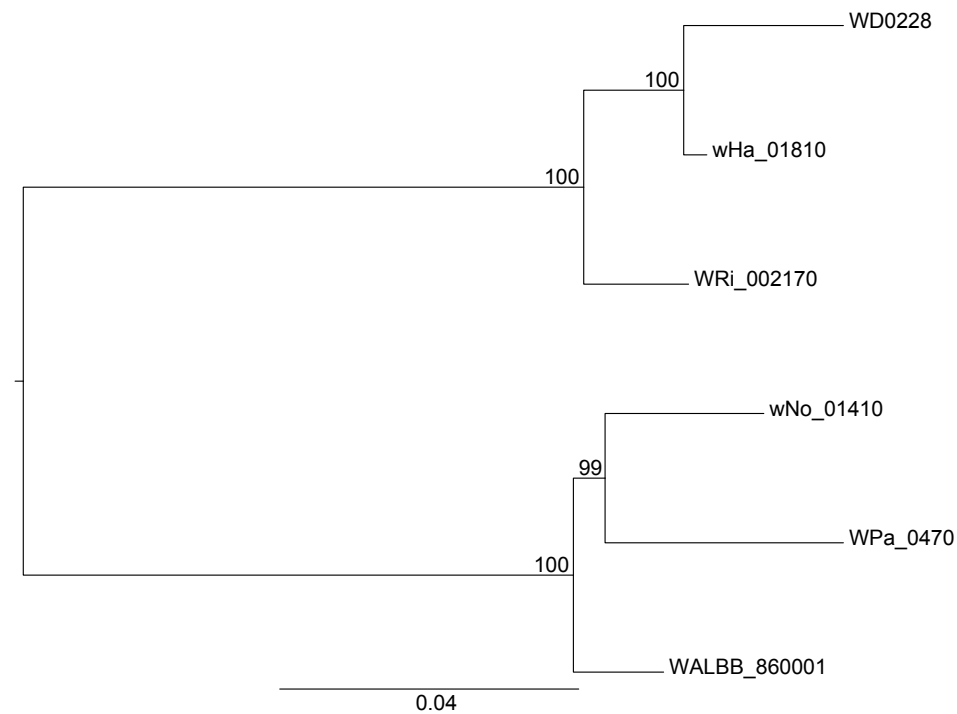

C

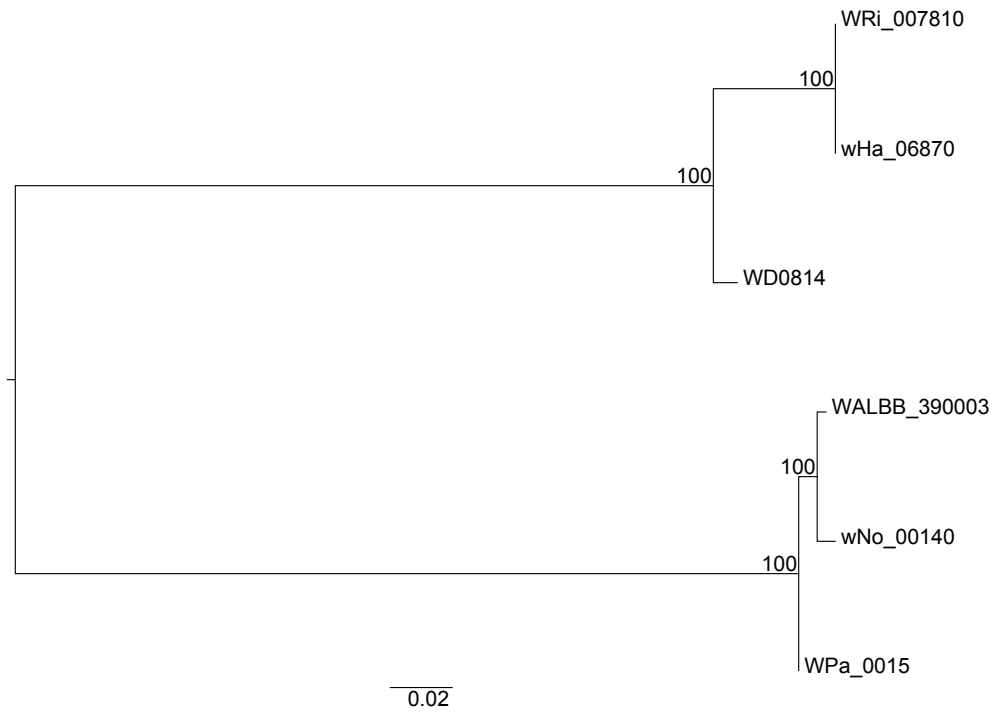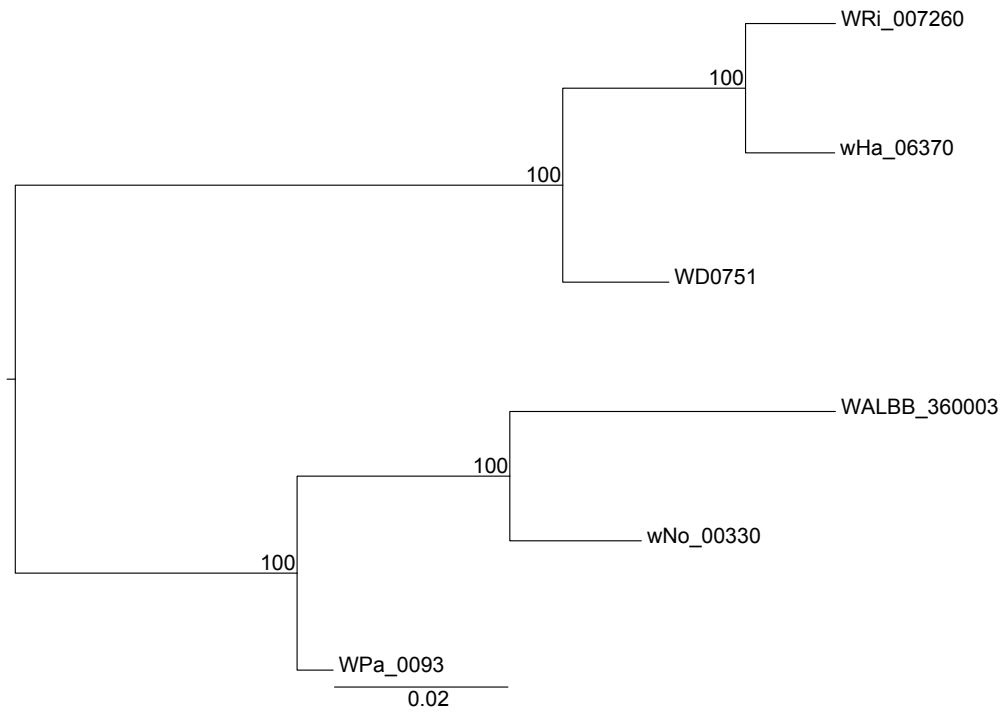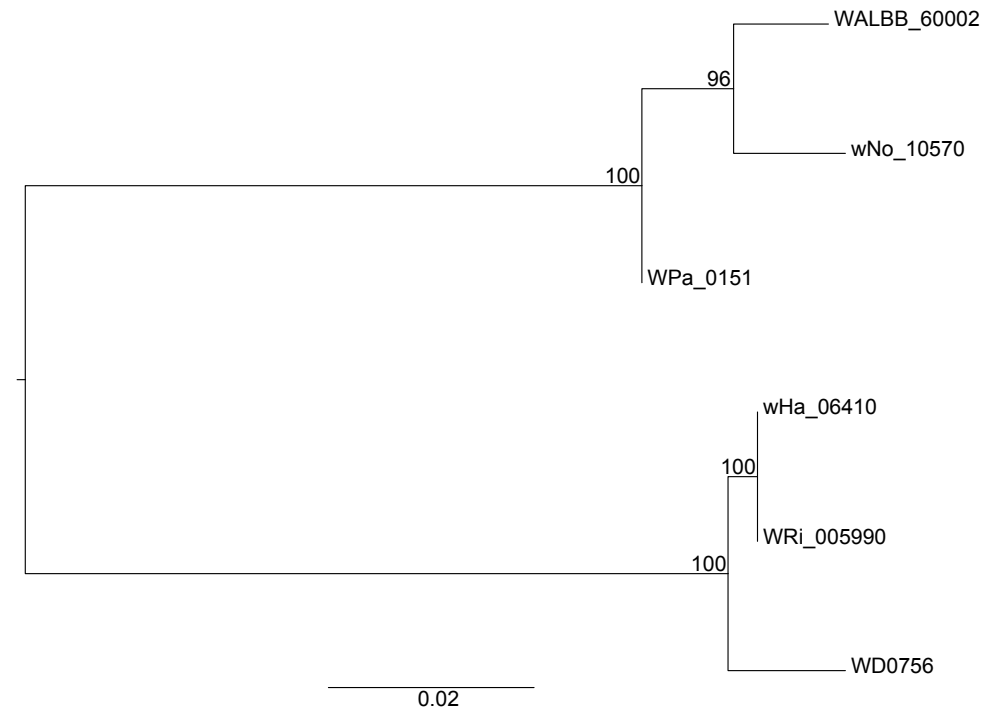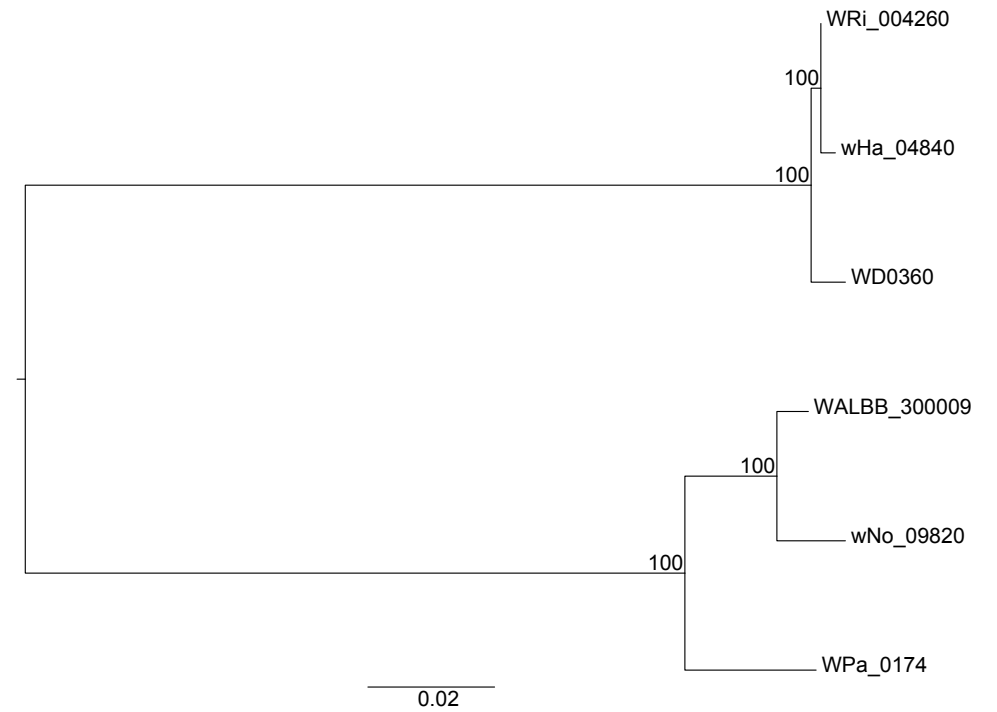

D

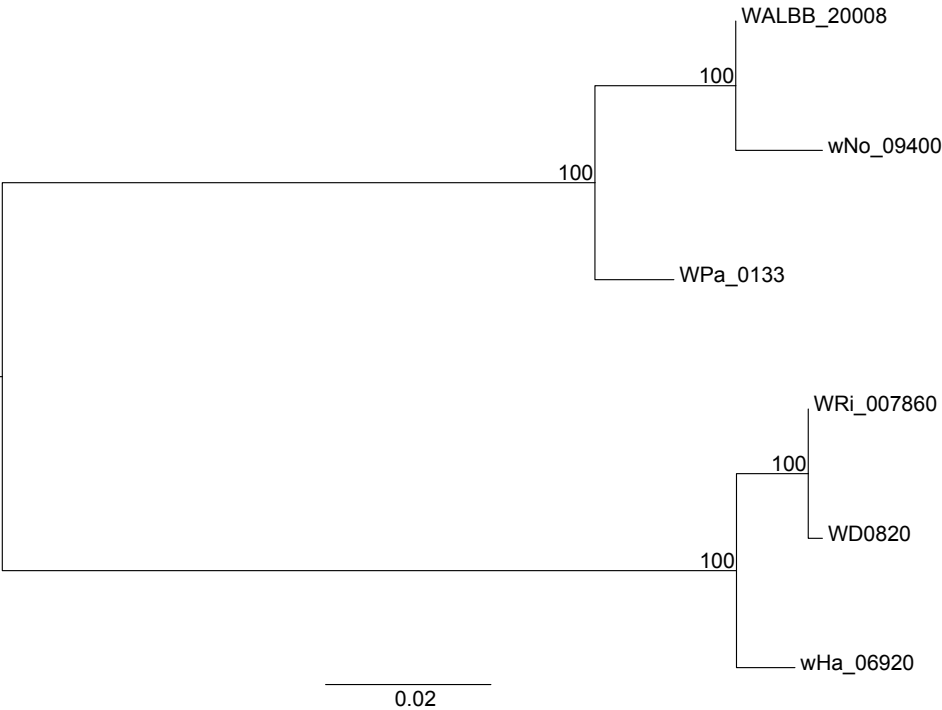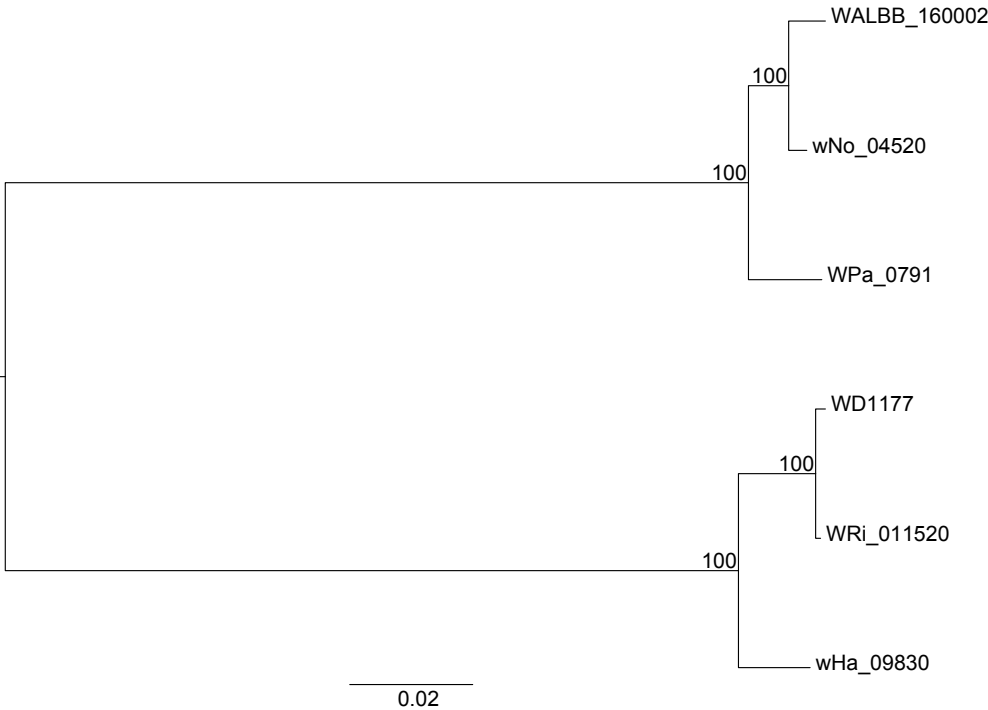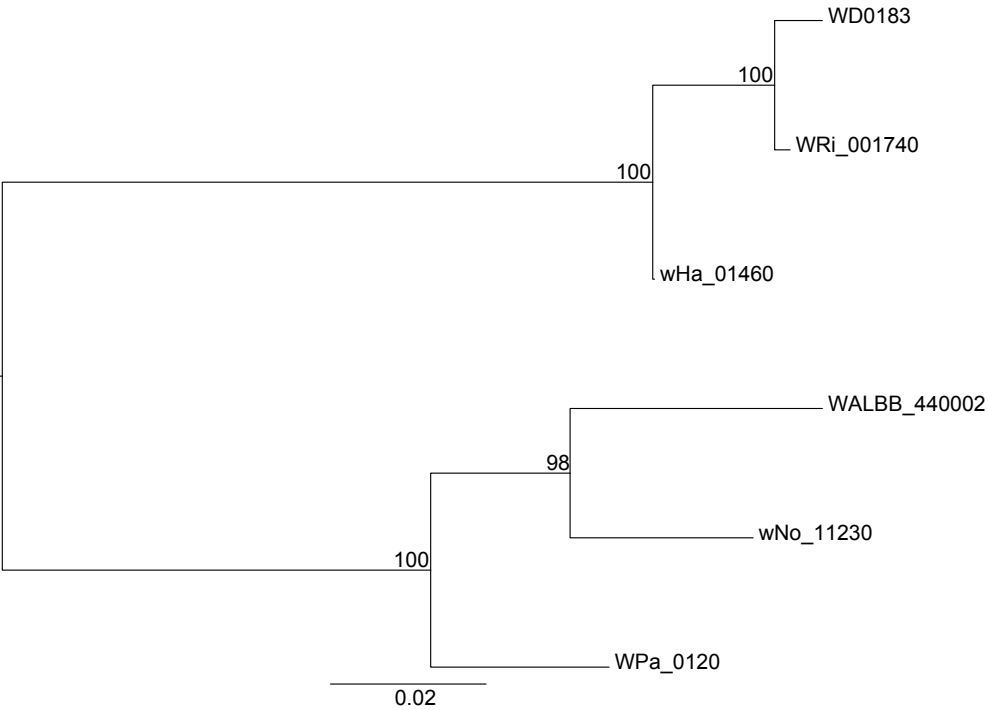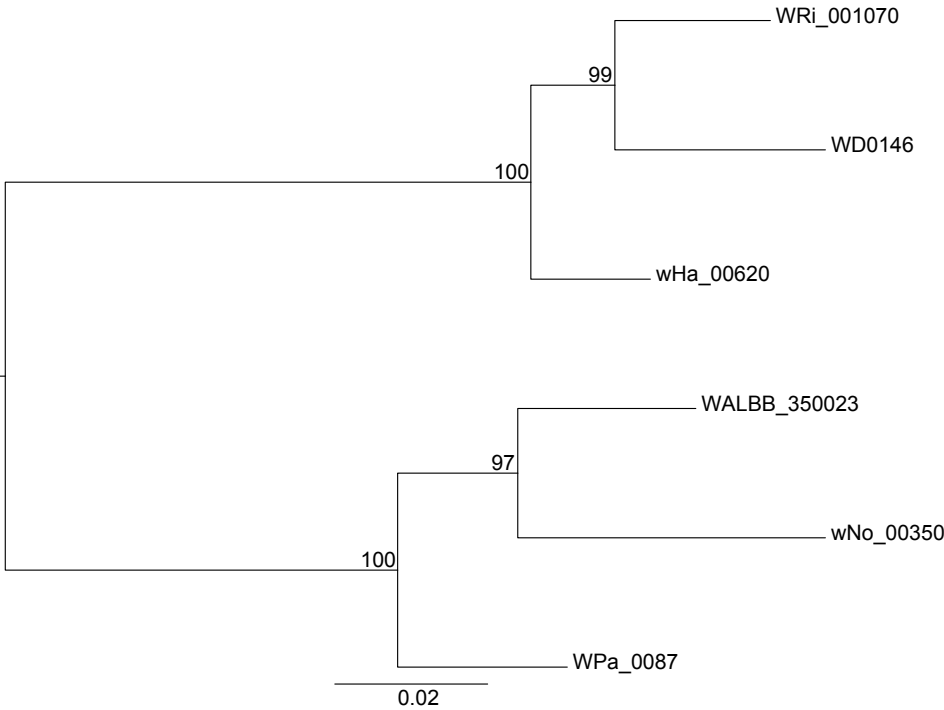

E

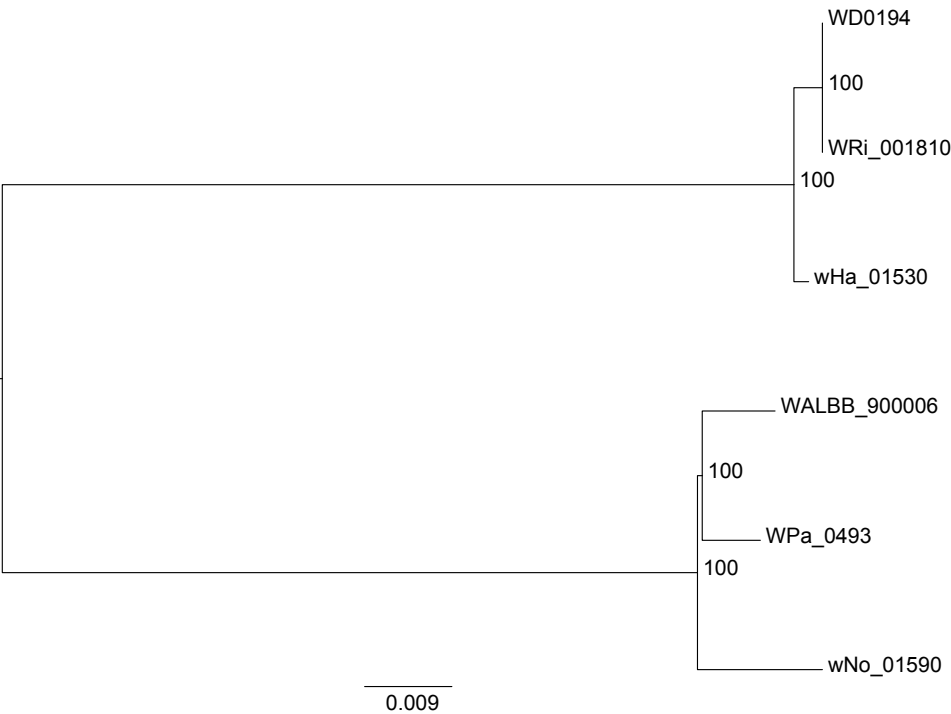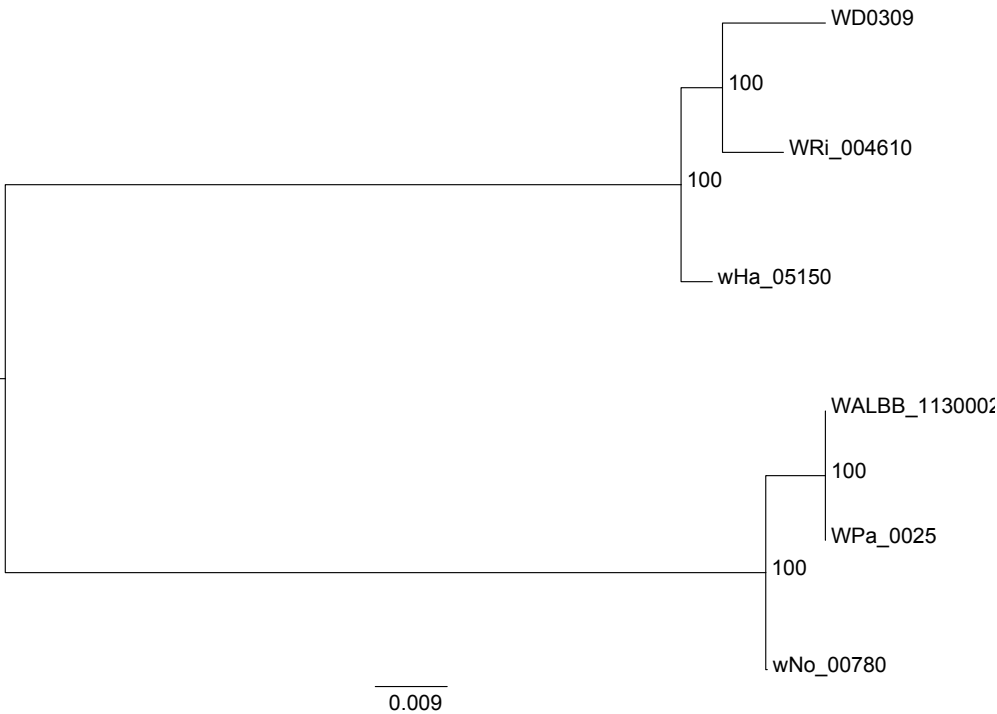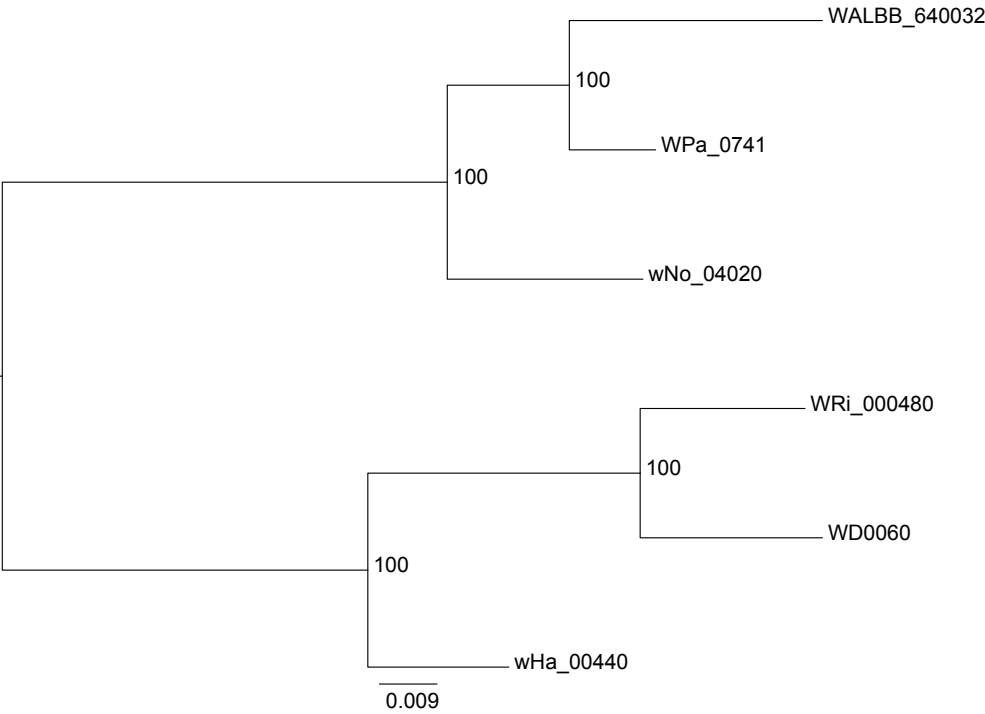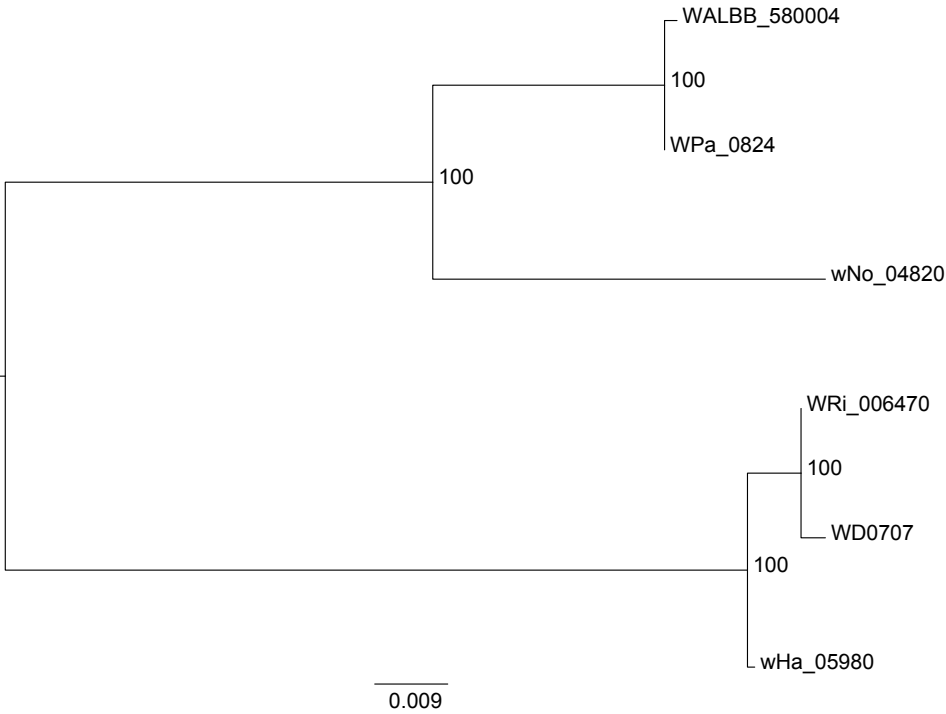

F

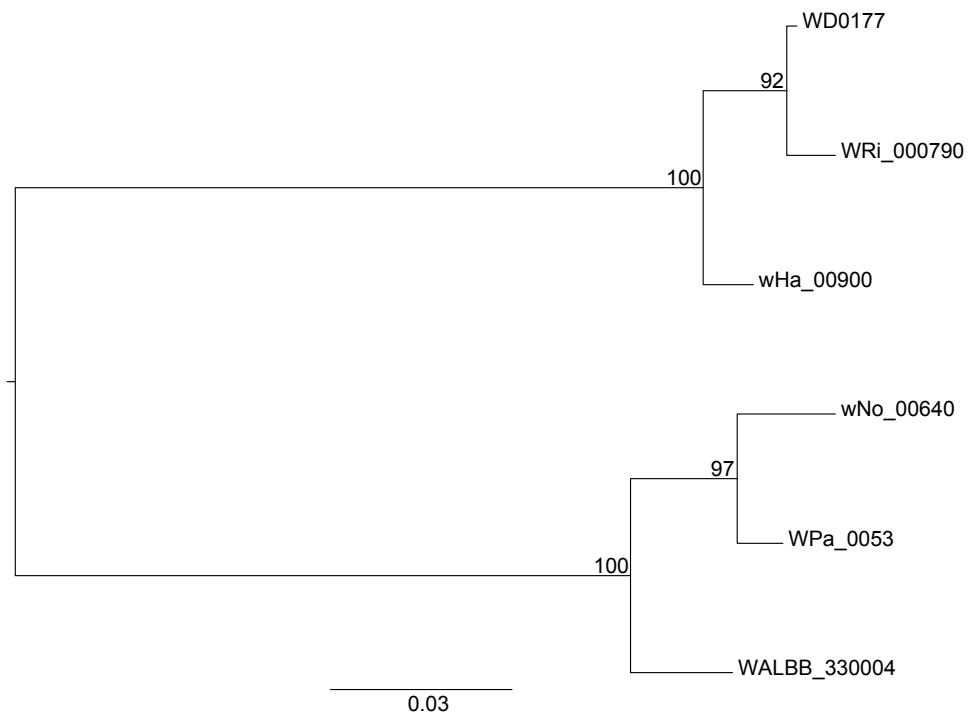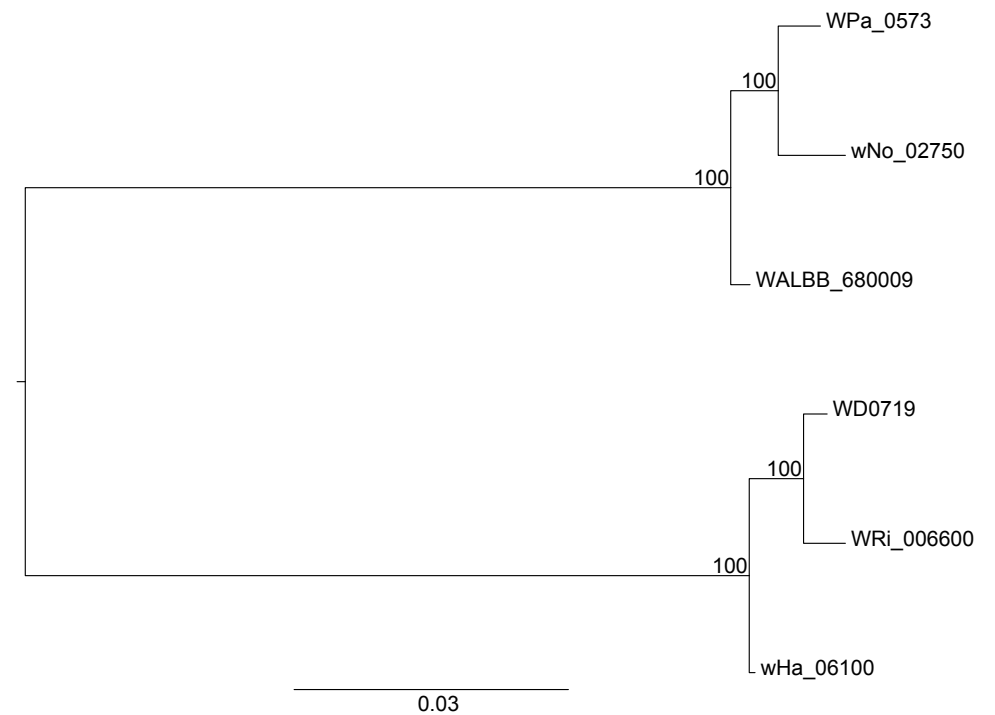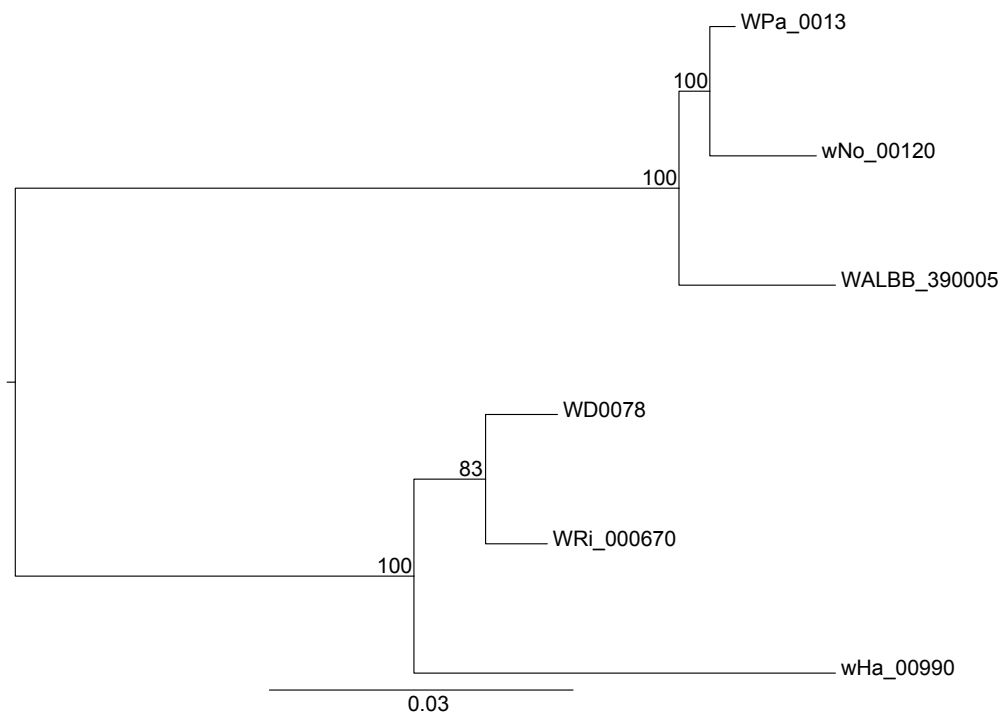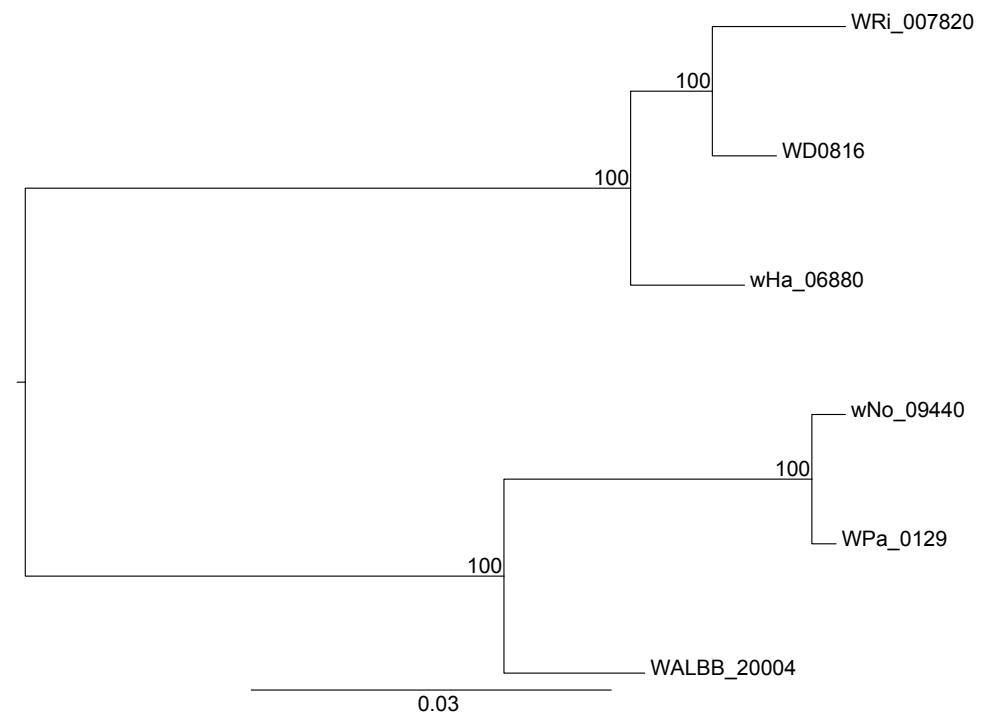

G

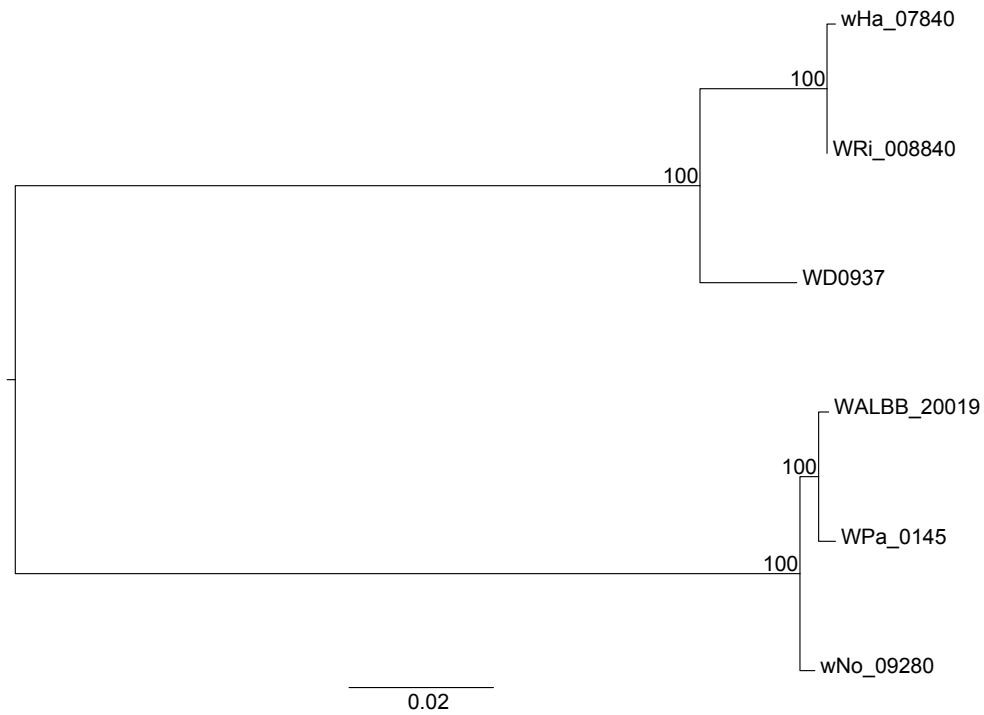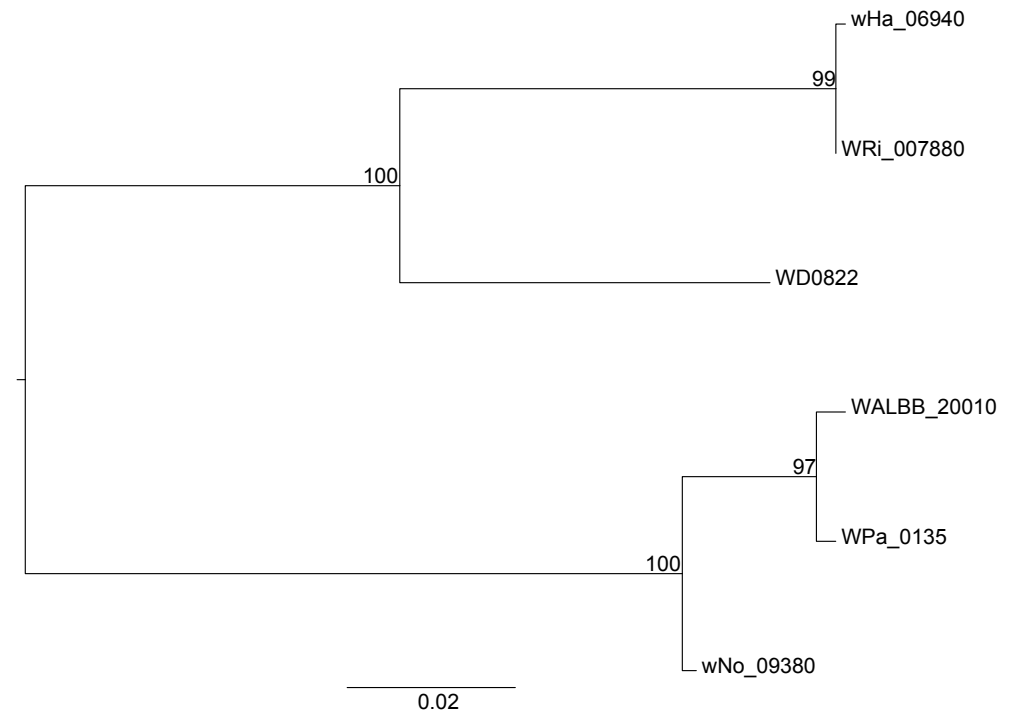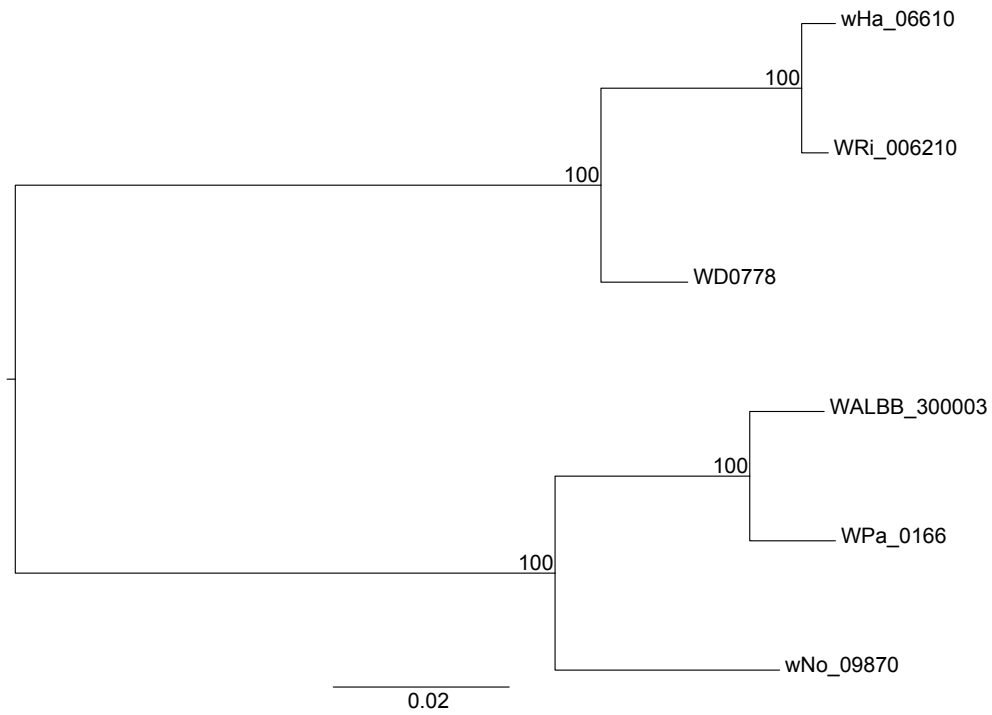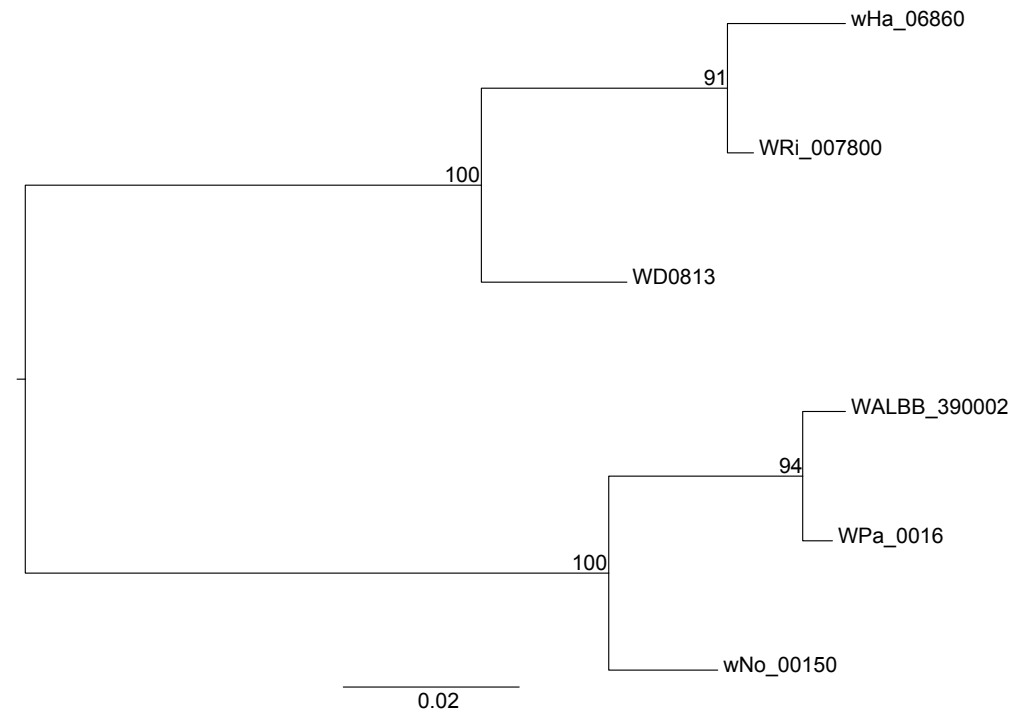

H

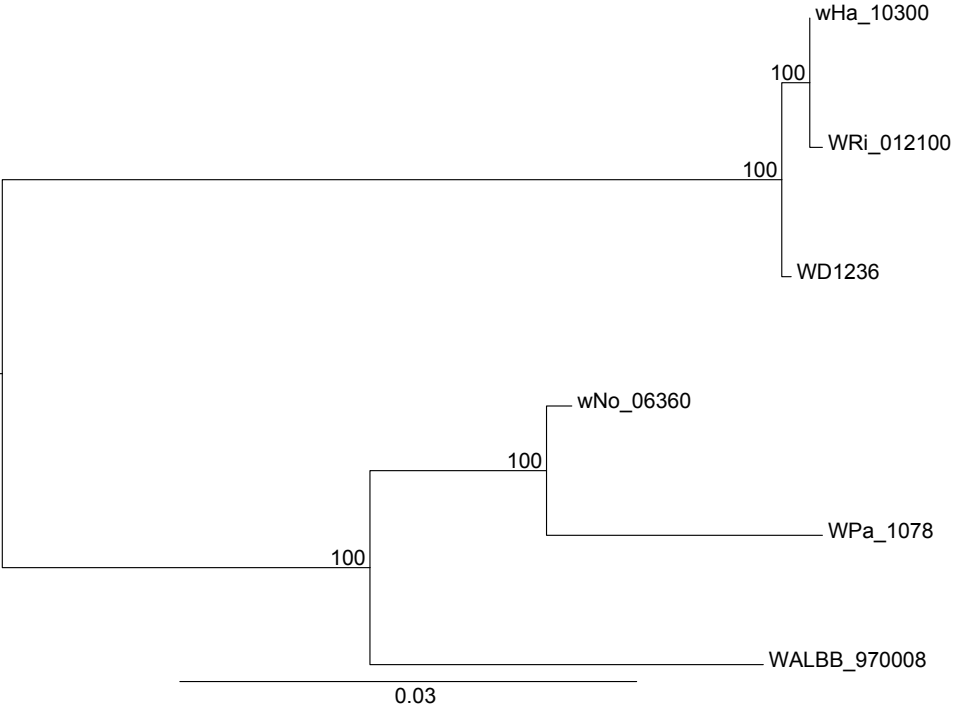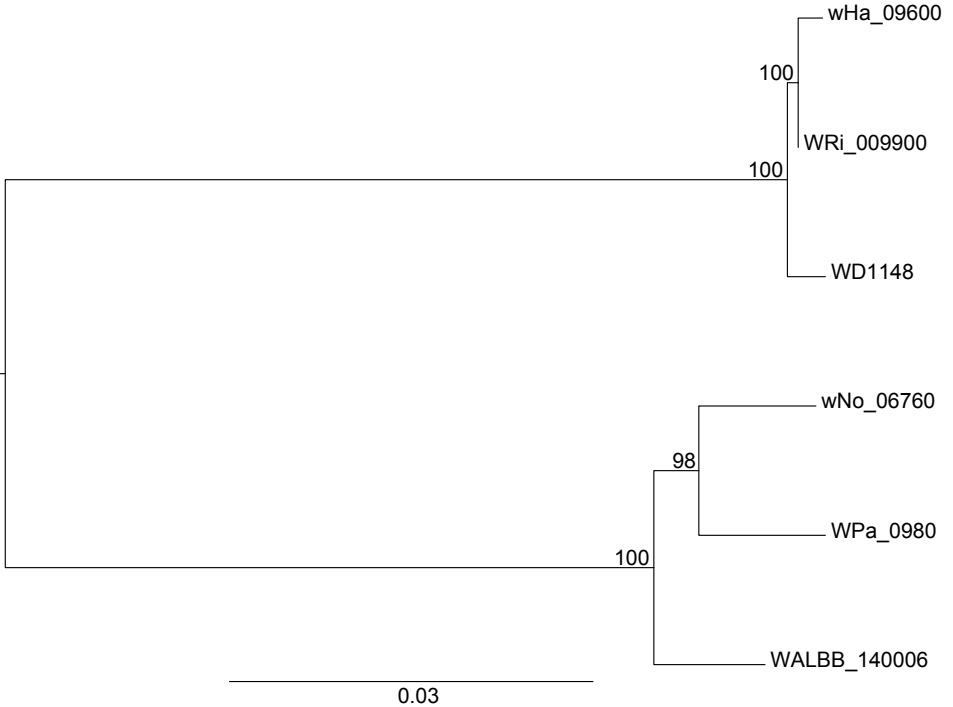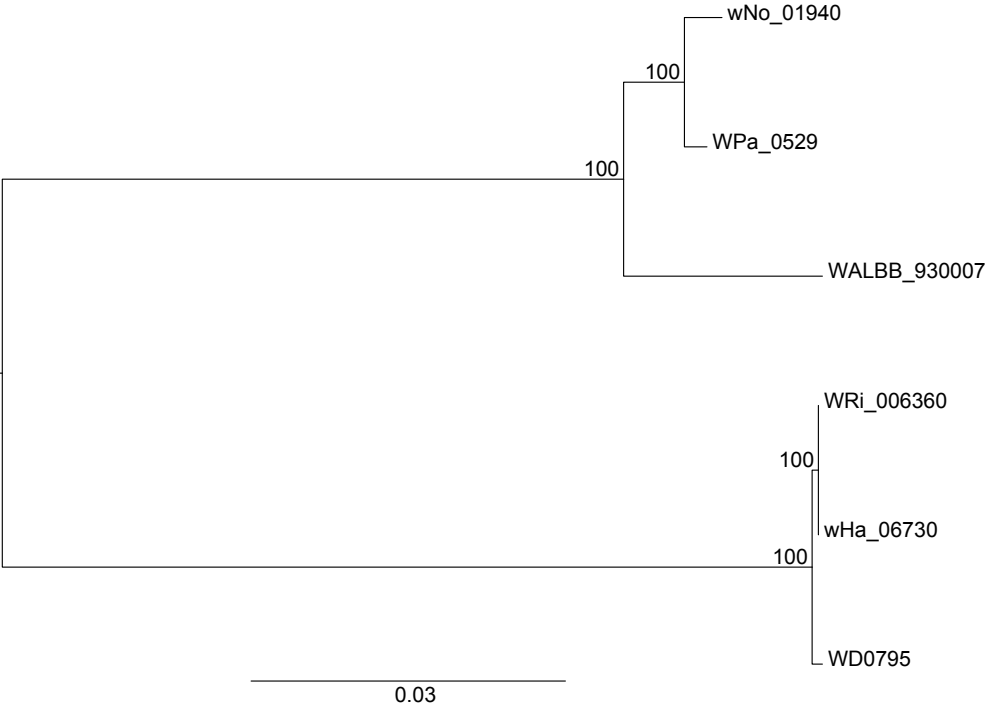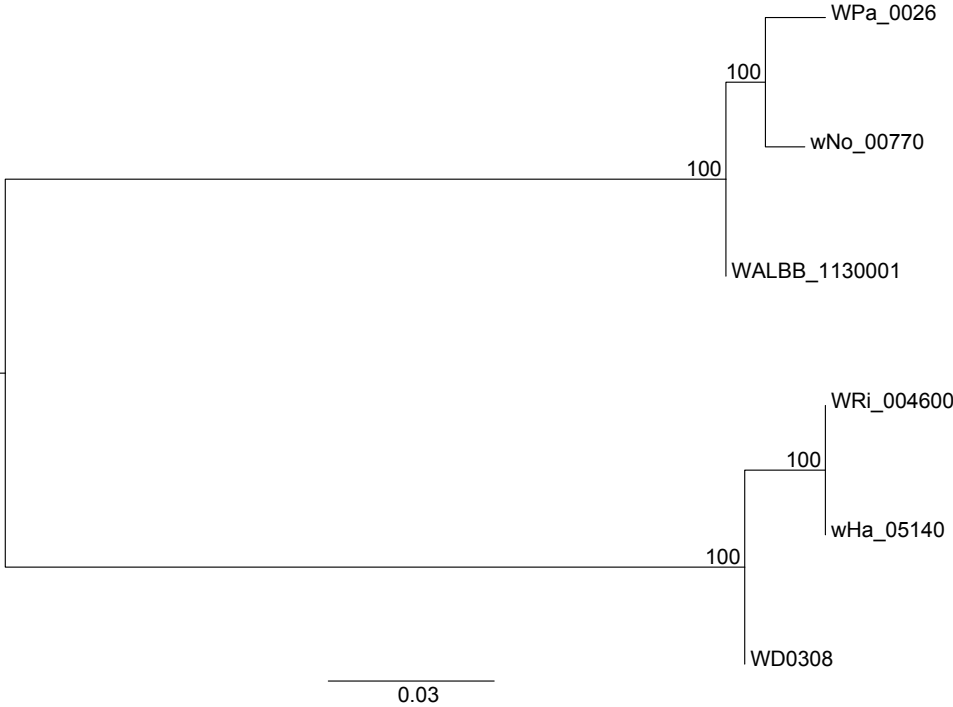

I

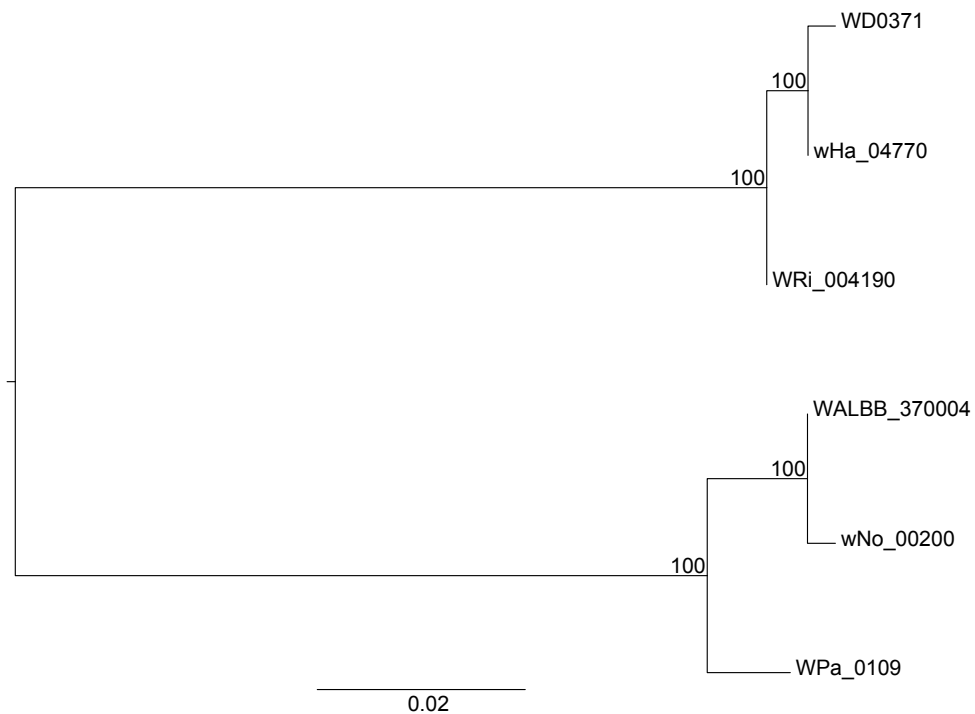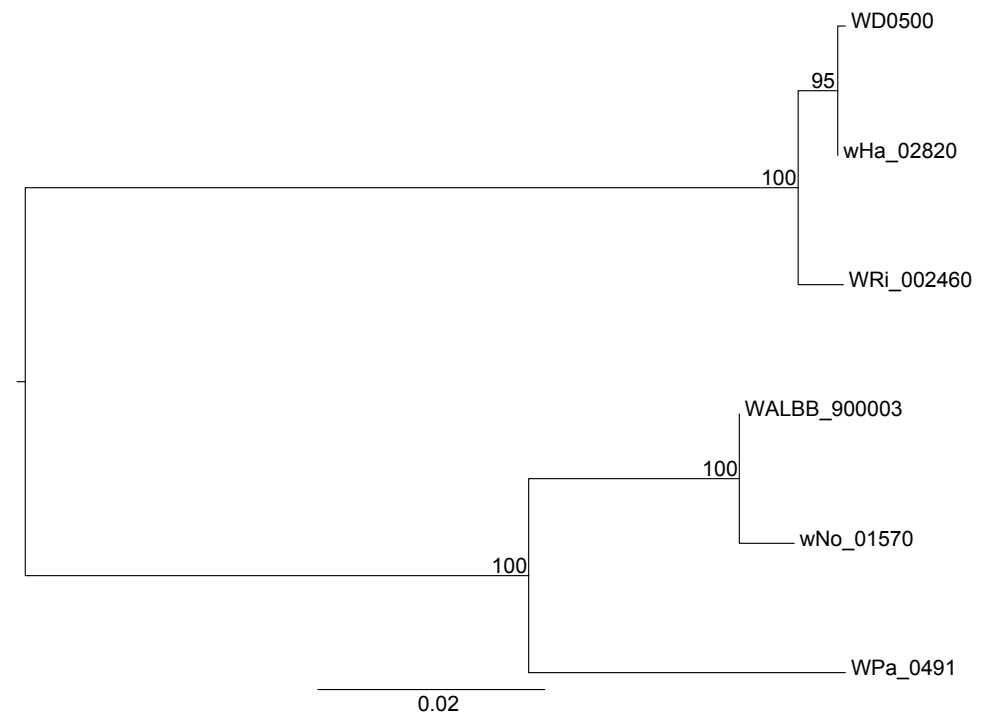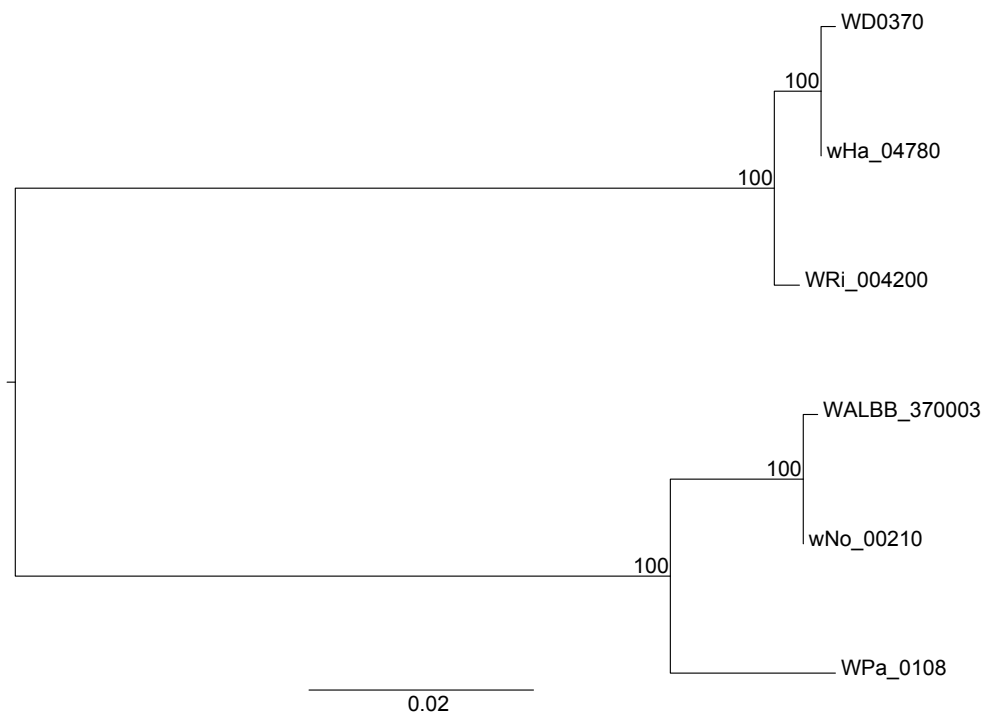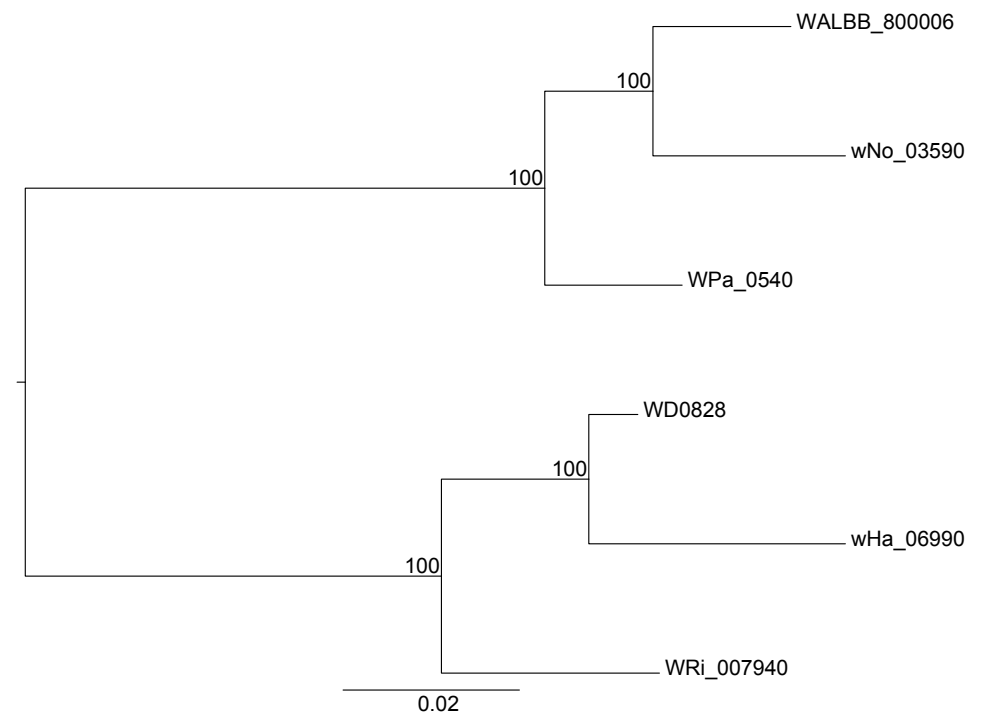

Supplement: Figure S4 — Example trees from the nine major clusters found for single-gene phylogenies. Four phylogenetic trees from each cluster are shown, where the different clusters are represented by the letters from A to I. Numbers on the nodes show the support from 100 bootstrap replicates. (PDF) [file pgen.1003381.s004.pdf]

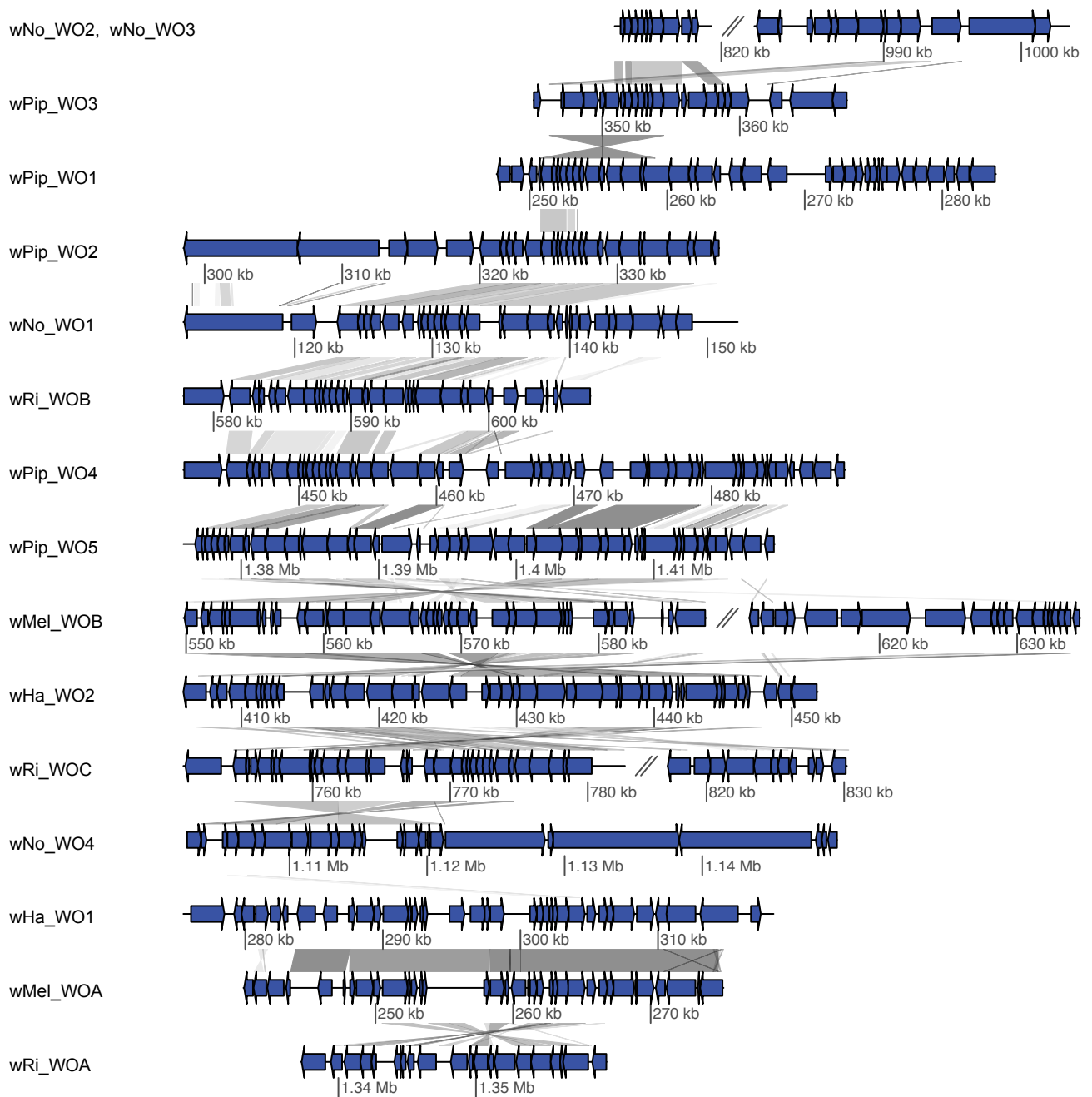

Supplement: Figure S5 — Overview of prophages from completed Wolbachia genomes. Blue arrows indicate annotated genes. Grey lines of different intensity indicate the similarity between sequences. (PDF) [file pgen.1003381.s005.pdf]

A

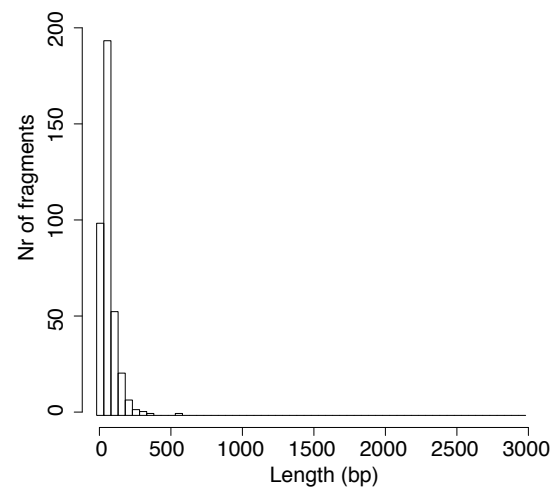

B

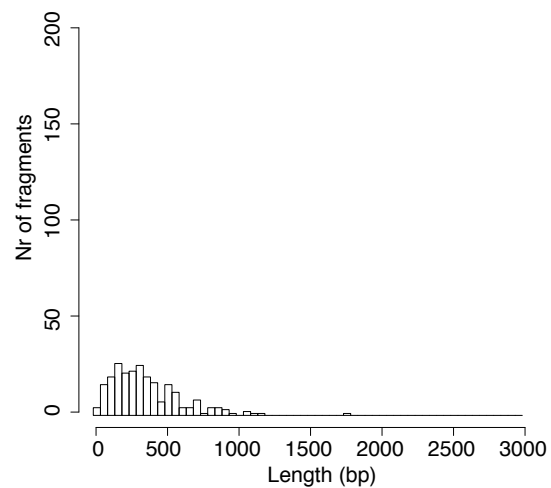

C

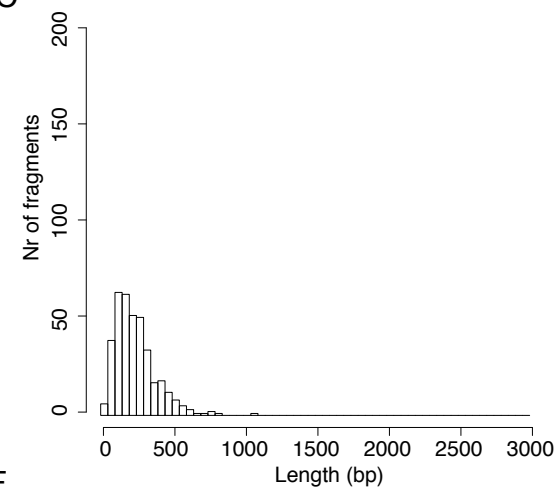

D

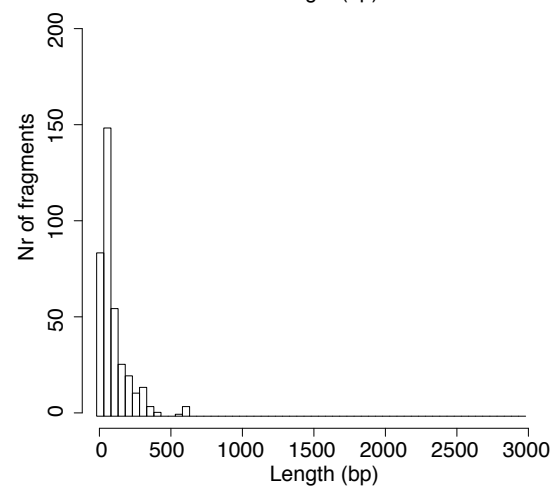

E

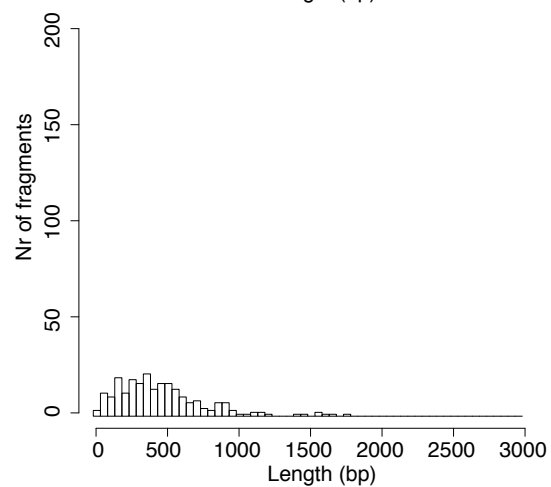

F

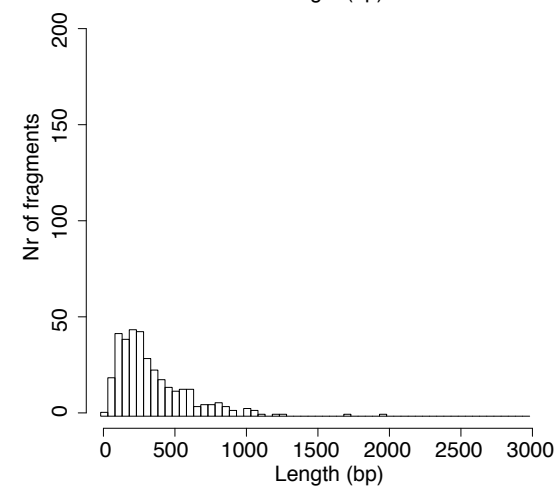

G

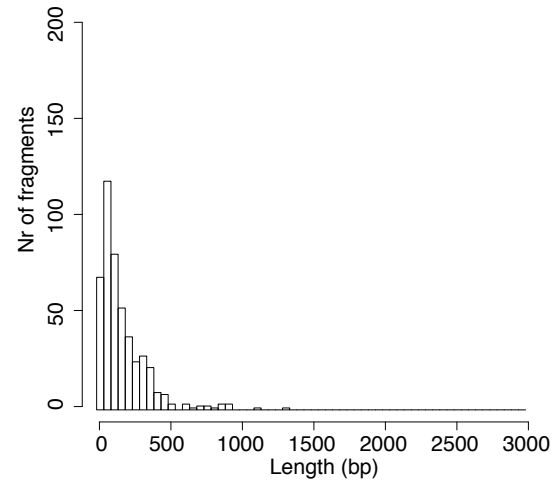

H

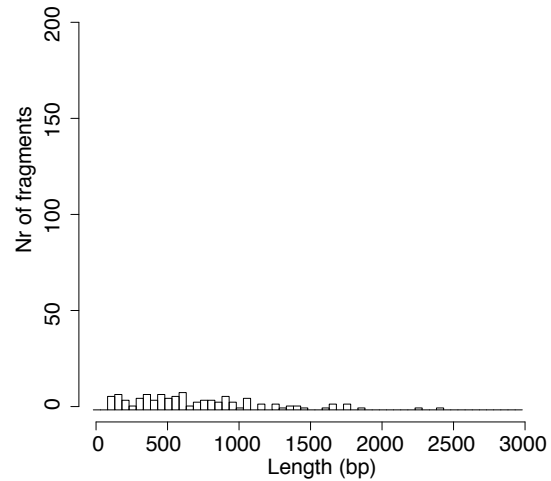

I

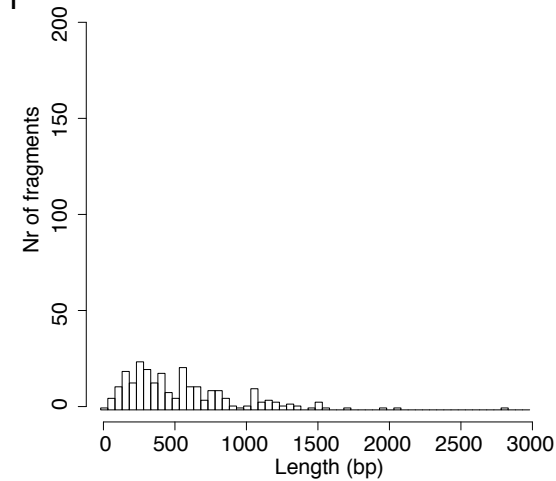

Supplement: Figure S6 — Histograms of recombining fragments predicted by GENECONV. The plots show the size distribution of recombining fragments, Top row; gscale = 0, Middle row; gscale = 3 and Bottom row; gscale = 1. A, D, G) between pairs of supergroup A and B strains; B, E, H) between pairs of supergroup A strains and C, F, I) between pairs of supergroup B strains. (PDF) [file pgen.1003381.s006.pdf]

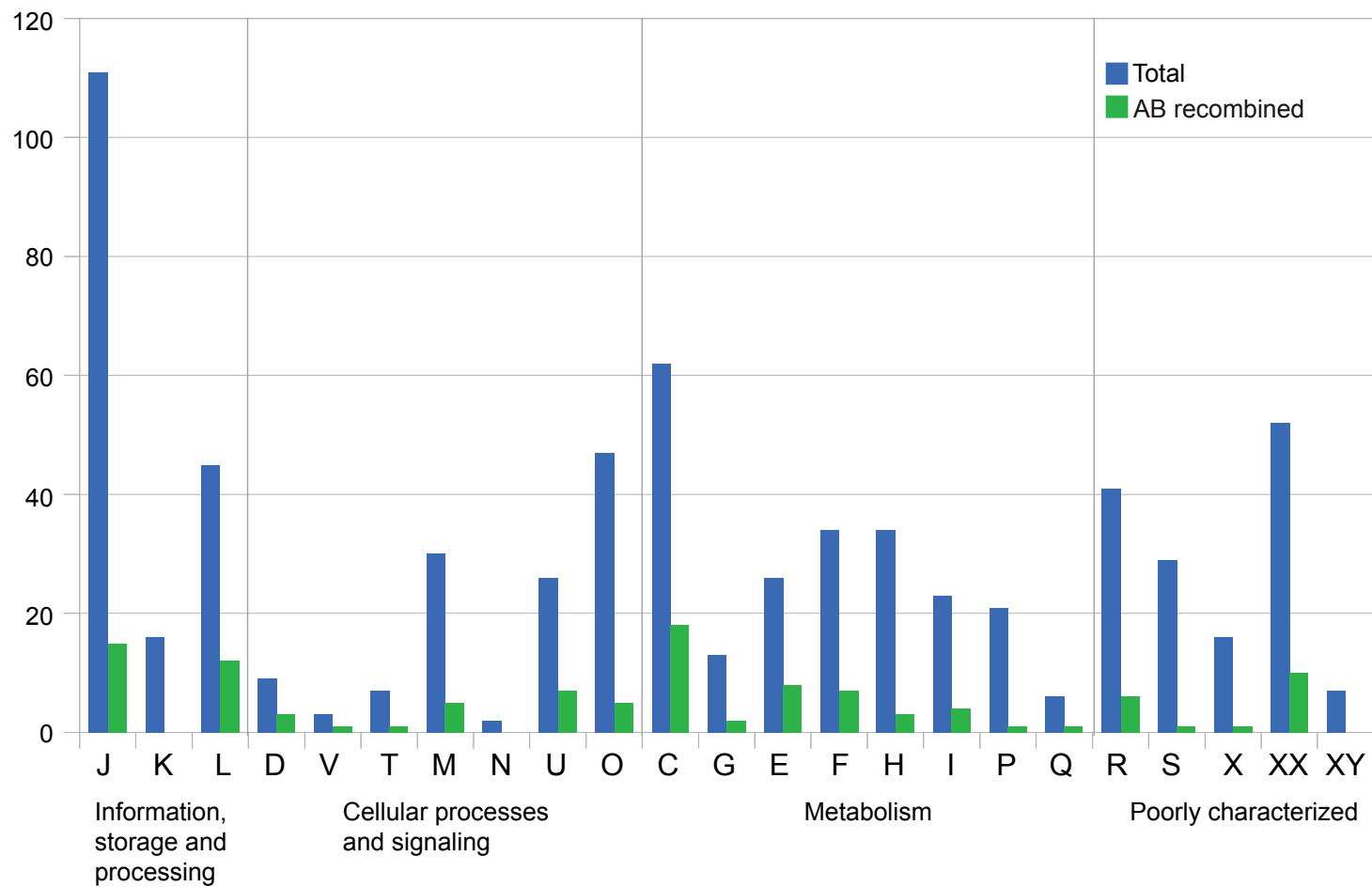

Supplement: Figure S7 — COG functional categories for genes found to have recombined between super-groups, as compared to non-recombined genes. The figure shows the number of genes in COG catergories, where blue bars represent the total core gene set, and green bars represent genes found to have been recombined between supergroup A and B strains. Non-standard COG categories are X = genes where hits has the designation NO_COG, XX = genes with less than 2 hits against the COG database and XY = genes where the two first hits fall into different COG categories. (PDF) [file pgen.1003381.s007.pdf]

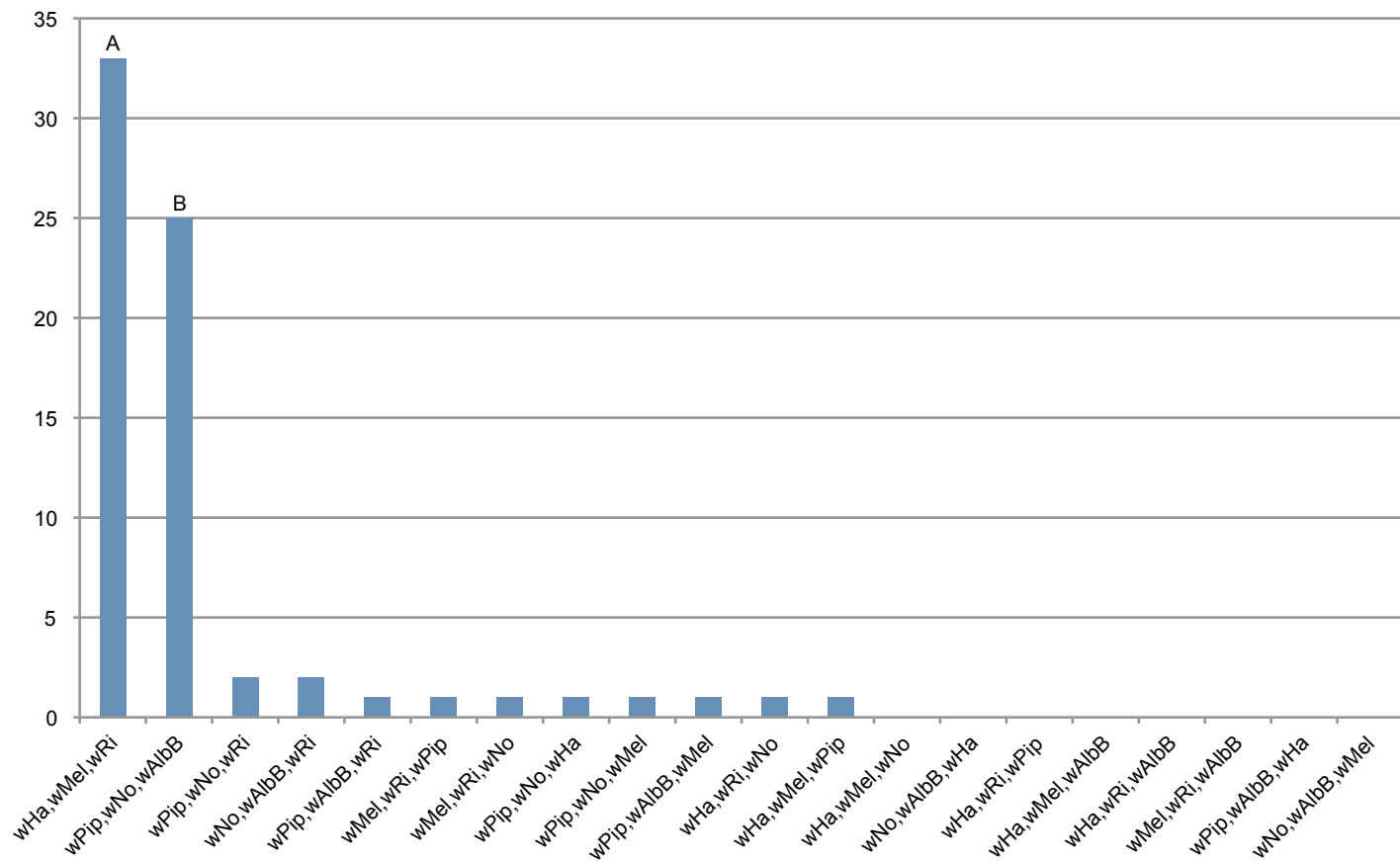

Supplement: Figure S8 — Protein clusters found in combinations of three Wolbachia strains. The bars show the number of protein cluster found to be shared for each three-strain combination; the A and B indicate the strain combinations of the A-supergroup and B-supergroup, respectively. (PDF) [file pgen.1003381.s008.pdf]

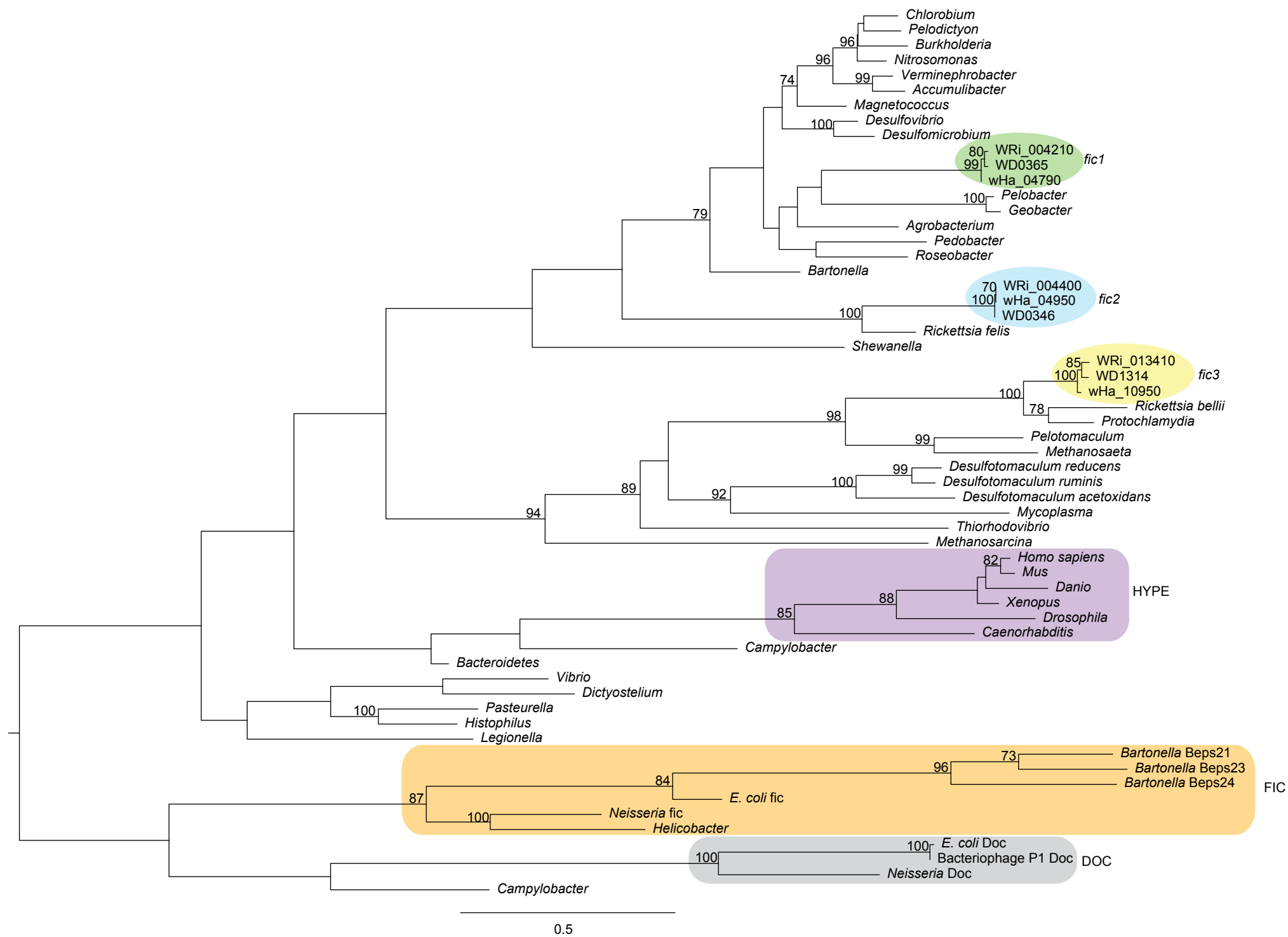

Supplement: Figure S9 — Phylogenetic analyses of the fic-domain proteins. The tree includes blast-identified homologues of the Wolbachia fic-domain protein genes, and members of the “FiDo” family (see methods). Highlighted genes: Green - Wolbachia fic1, Blue - Wolbachia fic2, Light yellow – Wolbachia fic3, Lilac – The HYPE subgroup, including human HYPE gene, Dark yellow – The Fic sub-group, including the Bartonella effector proteins, Grey- The Doc sub-group. Numbers on the nodes represent the support from 1000 bootstrap replicates. (PDF) [file pgen.1003381.s009.pdf]

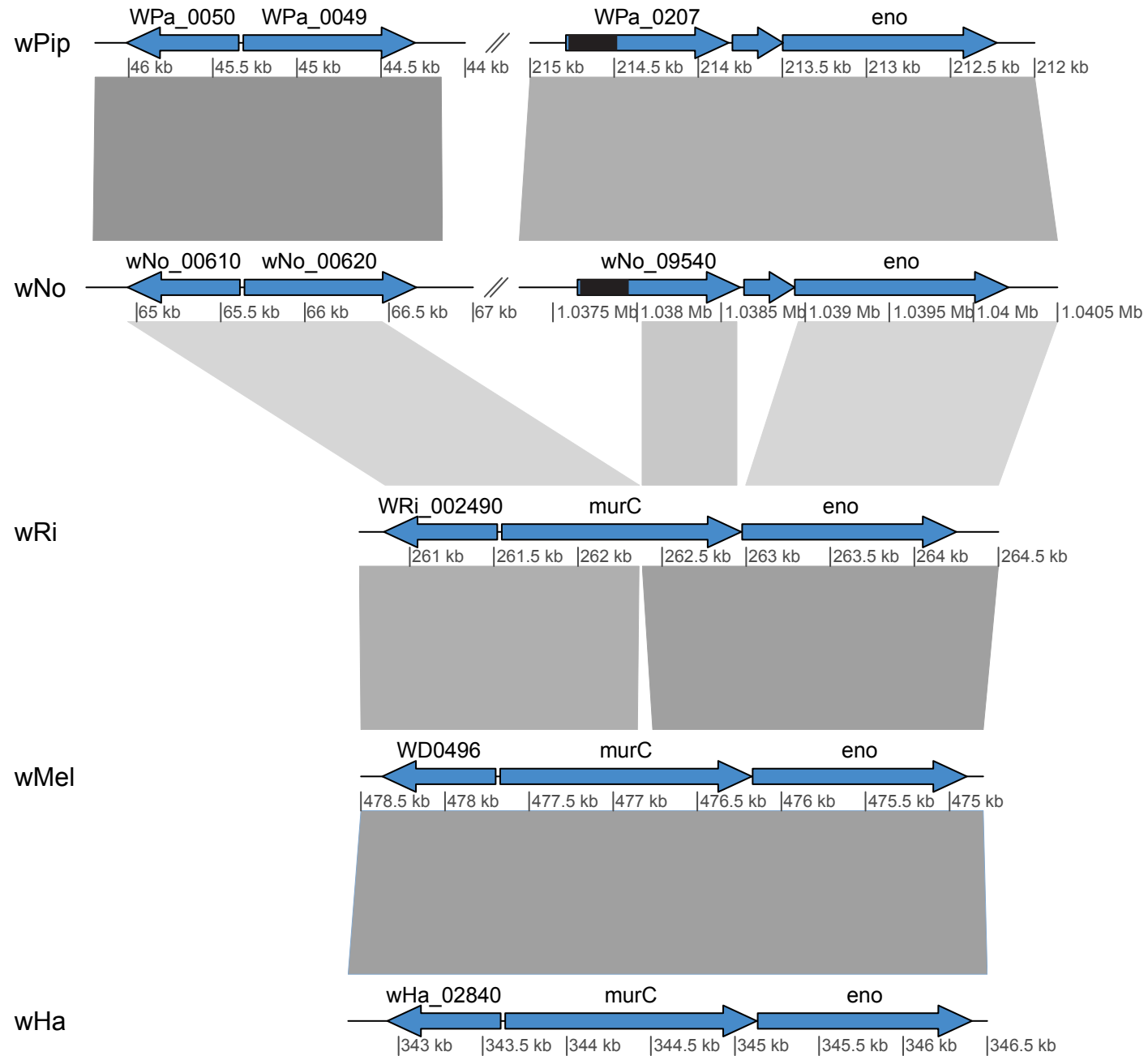

Supplement: Figure S10 — Gene map of the murC regions in supergroup A and B strains. Blue arrows indicate annotated genes and the black box of the genes in wNo and wPip mark the position of the recombinase beta zinc ribbon domain. Grey lines of different intensity indicate the similarity between sequences. (PDF) [file pgen.1003381.s010.pdf]

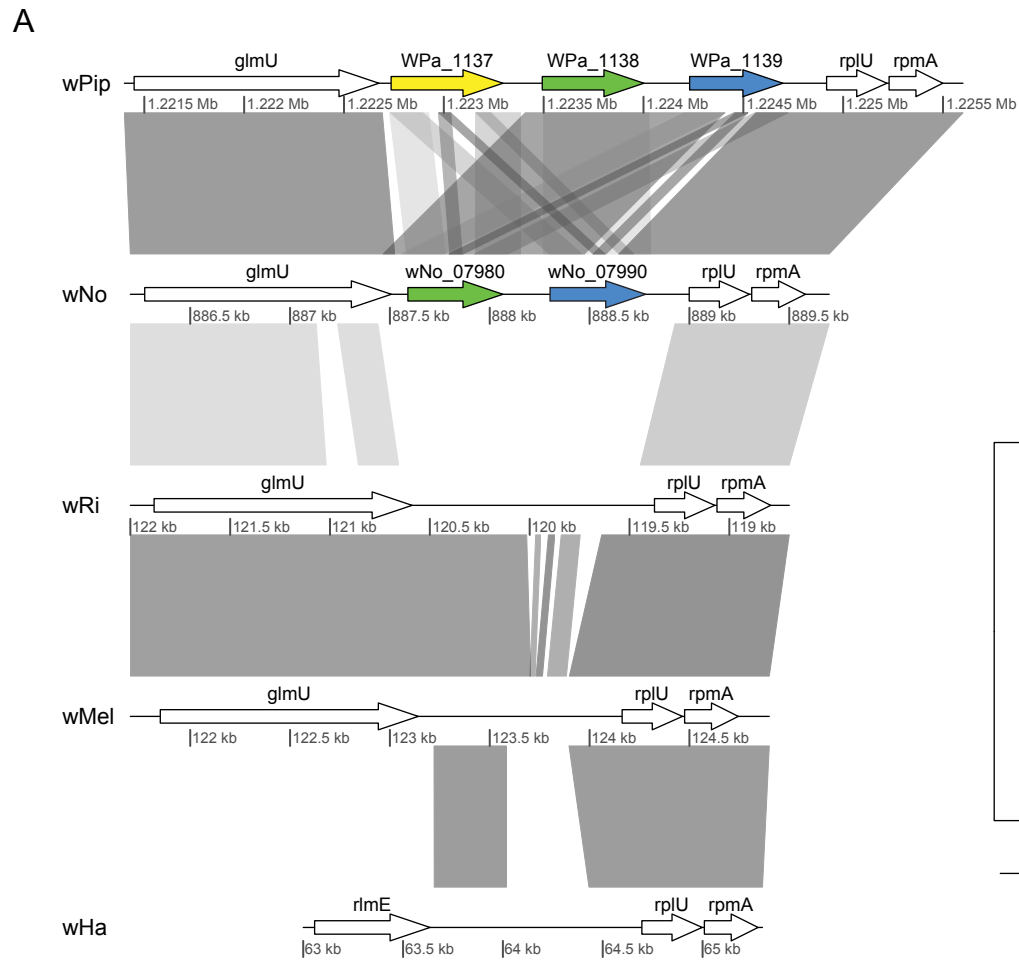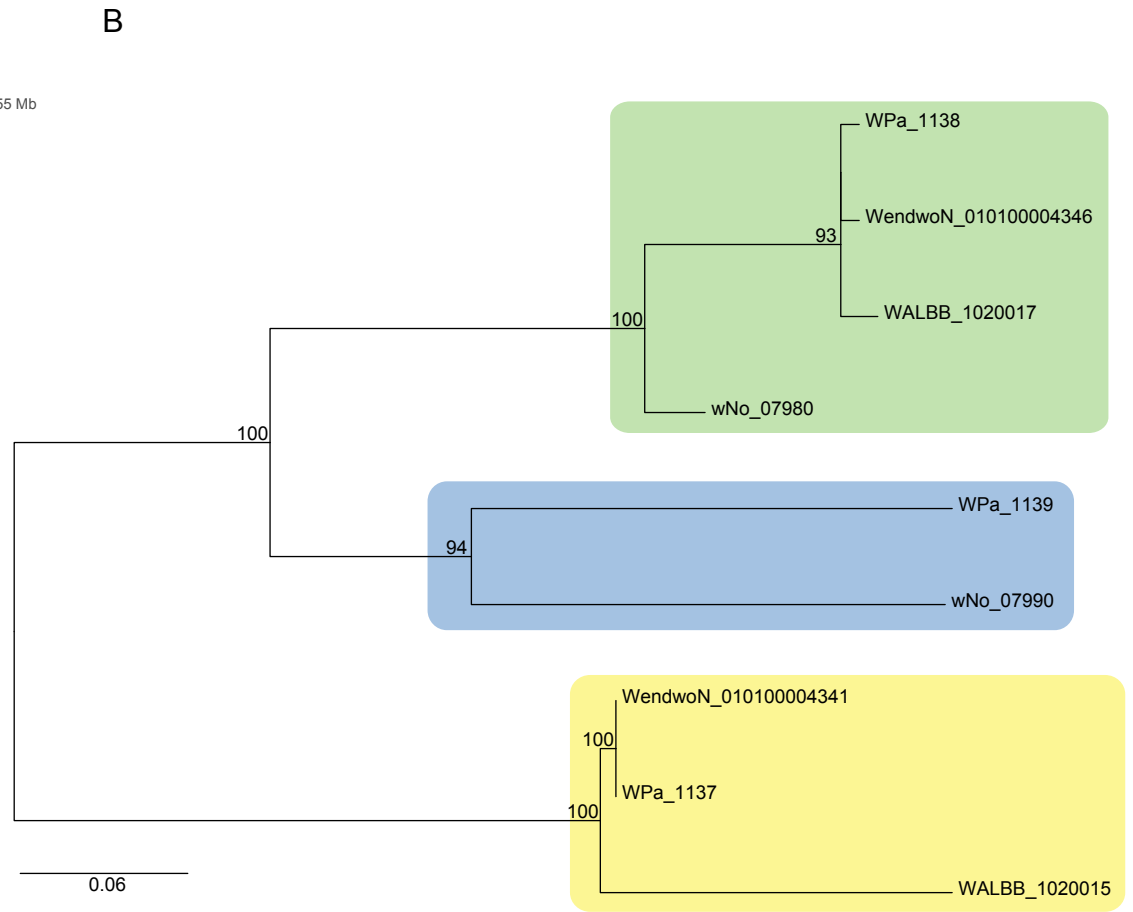

Supplement: Figure S11 — Outer membrane proteins specific for the B group strains. A) Gene map of the outer membrane protein region in B groups strains compared to A group strains. Colored arrows indicate the outer membrane proteins in the B-group genomes. Grey lines of different intensity indicate the similarity between sequences. B) Maximum likelihood trees of the B-group specific outer membrane protein. The colored blocks follow the coloring scheme in Figure 9A, showing the grouping of orthologs between genomes. Numbers on the nodes represent the support from 100 bootstrap replicates. (PDF) [file pgen.1003381.s011.pdf]
