# Supplementary material for: Comparative Genomics of Wolbachia and the Bacterial Species Concept
Source: PLoS Genet. 2013 Apr 4;9(4):e1003381. doi: 10.1371/journal.pgen.1003381 (PMC3616963; doi:10.1371/journal.pgen.1003381)
Supplement: Table S1 — Sequencing data and assembly. Number of reads and read length statistics for 454 sequencing data, shown separately for assembling and non-assembling reads (using the Mira assembler). (DOCX) [file pgen.1003381.s012.docx]

**Supplementary Table S1. Sequencing data and assembly.**

| Strain | Sequencing method | Data-set | Nb. of reads | mean read length | median read length |
| --- | --- | --- | --- | --- | --- |
| wHa | 454 single-end | In assembly | 77.397 | 371 | 398 |
|  |  | *Not assembled* | 3099 | 155 | 63 |
| wHa | 454 paired-end | In assembly | 143.688 | 346 | 315 |
|  |  | *Not assembled* | 19.999 | 172 | 86 |
| wNo | 454 single-end | In assembly | 138.026 | 346 | 374 |
|  |  | *Not assembled* | 4694 | 156 | 63 |
| wNo | 454 paired-end | In assembly | 239.068 | 311 | 280 |
|  |  | *Not assembled* | 38.497 | 161 | 84 |
|  |  |  |  |  |  |
|  |  |  |  |  |  |
|  |  |  |  |  |  |
|  |  |  |  |  |  |
