# Supplementary material for: Comparative Genomics of Wolbachia and the Bacterial Species Concept
Source: PLoS Genet. 2013 Apr 4;9(4):e1003381. doi: 10.1371/journal.pgen.1003381 (PMC3616963; doi:10.1371/journal.pgen.1003381)
Supplement: Table S2 — IS elements. Number of identified putative functional and non-functional IS-elements for each of the genomes wHa and wNo. Sequences were assigned to IS-families based by TBlastX searches on IS-finder (https://www-is.biotoul.fr). An IS-element was considered to be functional if the query sequence could be aligned to the complete annotated IS-element. IS-elements were considered non-functional if the alignment was partial or contained frame-shifts. (DOCX) [file pgen.1003381.s013.docx]

**Supplementary Table S2. IS elements.**

| IS family | *w*No, functional | *w*No, non-functional | *w*Ha, functional | *w*Ha, non-functional |
| --- | --- | --- | --- | --- |
| IS3 | 1 | 0 | 0 | 17 |
| IS4 | 0 | 1 | 4 | 8 |
| IS5 | 2 | 2 | 0 | 11 |
| IS6 | 0 | 6 | 0 | 0 |
| IS66 | 0 | 1 | 0 | 4 |
| IS110 | 11 | 1 | 2 | 9 |
| IS256 | 0 | 2 | 0 | 1 |
| IS630 | 0 | 2 | 0 | 7 |
| IS982 | 0 | 0 | 0 | 1 |
| ISNCY | 0 | 0 | 3 | 0 |
