# Supplementary material for: Comparative Genomics of Wolbachia and the Bacterial Species Concept
Source: PLoS Genet. 2013 Apr 4;9(4):e1003381. doi: 10.1371/journal.pgen.1003381 (PMC3616963; doi:10.1371/journal.pgen.1003381)
Supplement: Table S3 — Pairwise non-synonymous (dN) and synonymous (dS) substitution frequencies. The upper right section of the table shows the pairwise dN-values, whereas the bottom left section shows the pairwise dS-values between strains. (DOCX) [file pgen.1003381.s014.docx]

**Supplementary Table S3. Pairwise non-synonymous (dN) and synonymous (dS) substitution frequencies.**

|  | *w*Ha | *w*Ri | *w*Mel | *w*No | *w*Pip | *w*AlbB |
| --- | --- | --- | --- | --- | --- | --- |
| *w*Ha | - | 0,0053 | 0,0054 | 0,0683 | 0,0651 | 0,0659 |
| *w*Ri | 0,0296 | - | 0,0059 | 0,0673 | 0,0641 | 0,0650 |
| *w*Mel | 0,0326 | 0,0336 | - | 0,0682 | 0,0663 | 0,0662 |
| *w*No | 0,5837 | 0,5804 | 0,5833 | - | 0,0123 | 0,0142 |
| *w*Pip | 0,5660 | 0,5679 | 0,5706 | 0,0745 | - | 0,0104 |
| *w*AlbB | 0,5787 | 0,5774 | 0,5727 | 0,0783 | 0,0609 | - |
