# Supplementary material for: Comparative Genomics of Wolbachia and the Bacterial Species Concept
Source: PLoS Genet. 2013 Apr 4;9(4):e1003381. doi: 10.1371/journal.pgen.1003381 (PMC3616963; doi:10.1371/journal.pgen.1003381)
Supplement: Table S4 — Summary of GENECONV results for intragenic recombination events with different gscale settings. GENECONV was tested on all 660 gene single-gene orthologs present in all supergroup A (wHa, wRi, wMel) and B (wNo, wPip, wAlb) with three different gscale settings (see materials and methods). Only gene alignments with support for recombination with at least two additional methods were evaluated. (DOCX) [file pgen.1003381.s015.docx]

**Supplementary Table S4. Summary of GENECONV results for intragenic recombination events with different gscale settings.**

| gscale | Nr of genes | Nr of fragments | Median length of fragments (bp) | Mean length of fragments (bp) |
| --- | --- | --- | --- | --- |
| A-A recombinations | | | | |
| 0 | 105 | 244 | 320 | 370 |
| 3 | 110 | 240 | 408 | 481 |
| 1 | 69 | 131 | 623 | 728 |
| B-B recombinations | | | | |
| 0 | 147 | 378 | 221 | 246 |
| 3 | 154 | 367 | 292 | 365 |
| 1 | 120 | 270 | 470 | 574 |
| A-B recombinations | | | | |
| 0 | 96 | 386 | 73 | 86 |
| 3 | 101 | 379 | 84 | 121 |
| 1 | 111 | 472 | 124 | 184 |
