# Supplementary material for: Comparative Genomics of Wolbachia and the Bacterial Species Concept
Source: PLoS Genet. 2013 Apr 4;9(4):e1003381. doi: 10.1371/journal.pgen.1003381 (PMC3616963; doi:10.1371/journal.pgen.1003381)
Supplement: Table S5 — A-group specific genes. All genes found to be present in all supergroup A strains, while being absent from all supergroup B and D strains are listed with locus tag numbers. For those genes where a pseudogenized homolog could be identified in either supergroup B or D, “pos.” indicates that the homolog is present in a region of synteny between the genomes, whereas “Not pos.” indicates no synteny around the detected homolog. If a homolog could be identified in the Anaplasmataceae family, the members containing the gene are noted: Ac, Anaplasma centrale, Am, Anaplasma marginale, Eca, Ehrlichia canis, Ech., Ehrlichia chaggeensis. When applicable, the best significant blast hit outside the Wolbachia group is indicated. (DOCX) [file pgen.1003381.s016.docx]

**Supplementary Table S5**. **A-group specific genes**

| **Protein** | **wHa** | **wMel** | **wRi** | **B-group** | **wBm** | **Anaplasmataceae** | **Best hit (nr)** | **E-value** |
| --- | --- | --- | --- | --- | --- | --- | --- | --- |
| Arginine repressor | wHa_03250 | WD0453 | WRi_002710 | - | - | - | *Legionella pneumophila* str. Lens | 2e-47 |
| ABC transporter, ATP binding protein | wHa_03220 | WD0455 | WRi_002740 | - | - | - | *Fluoribacter dumoffii* Tex-KL | 4e-74 |
| ABC transporter, permease | wHa_03230 | WD0454b | WRi_002730 | - | - | - | *Legionella pneumophila* (several strains) | 7e-75 |
| ABC transporter, amino acid binding protein | wHa_03240 | WD0454a | WRi_002720 | - | - | - | *Legionella pneumophila* (several strains) | 3e-66 |
| Cytochrome d ubiquinol oxidase, subunit I | wHa_06280 | WD0740 | WRi_007360 | - | - | - | *Psychrobacter arcticus* 273-4 | 1e-168 |
| Cytochrome d ubiquinol oxidase, subunit II | wHa_06290 | WD0741 | WRi_007350 | - | - | - | *Desulfobacterium autotrophicum* HRM2 | 1e-115 |
| Fic family protein | wHa_10950 | WD1314 | WRi_013410 | - | - | - | Candidatus *Protochlamydia amoebophila UWE25* | 1e-161 |
| Fic family protein | wHa_04790 | WD0365 | WRi_004210 | - | pseudo (pos.) | - | *Desulfovibrio* sp. 3_1_syn3 | 1e-115 |
| Fic family protein | wHa_04950 | WD0346 | WRi_004400 | - | pseudo (not pos.) | - | *Rickettsia felis* URRWXCal2 | 1e-103 |
| Cell division protein FtsW | wHa_04570 | WD0394 | WRi_003950 | - | pseudo (pos.) | + (Ac, Am) | *Anaplasma centrale* str. Israel | 2e-75 |
| Penicillin-binding protein (ftsI) | wHa_10600 | WD1273 | WRi_012430 | - | pseudo (pos.) | + (Ac, Am) | *Anaplasma marginale* str. Florida | 1e-108 |
| Septum formation initiator family protein | wHa_04980 | WD0343 | WRi_004450 | - | pseudo (pos. side) | + (Ac, Am) | *Rhodospirillum photometricum* DSM 122 | 3e-09 |
| Putative F420-0:gamma-glutamyl ligase | wHa_07620 | WD0885 | WRi_008600 | - | pseudo (pos. side) | - | *Chlamydophila abortus* LLG | 1e-53 |
| DnaJ domain protein | wHa_08770 | WD1051 | WRi_010880 | - | - | - | - |  |
| Ankyrin repeat domain protein | wHa_06390 | WD0754 | WRi_007240 | pseudo (pos. side) | pseudo (pos.) | - | B-group *Wolbachia* strains | 1e-160 |
| Lipolytic enzyme, GDSL family | wHa_10790 | WD1297 | WRi_013240 | pseudo (pos. side) | pseudo (not pos.) | - | *Spiroplasma citri* | 2e-37 |
| Putative siRNA binding protein | wHa_00260 | WD0033 | WRi_000280 | - | - | - | - |  |
| Transposase | wHa_00890 | WD0176 | WRi_000800 | - | - | - | *Nitrosomonas europae* ATCC 19718 | 1e-129 |
| Hypothetical protein | wHa_01340 | WD0094 | WRi_001560 | - | - | - | - |  |
| Hypothetical protein | wHa_01620 | WD0206 | WRi_001960 | - | - | - | - |  |
| Hypothetical protein | wHa_02290 | WD0630 | WRi_010020, WRi_005360 | - | - | - | - |  |
| Hypothetical protein | wHa_03200 | WD0460 | WRi_002760 | - | - | - | - |  |
| Hypothetical protein | wHa_04720 | WD0377 | WRi_004120 | - | - | - | - |  |
| Hypothetical protein | wHa_06130 | WD0722 | WRi_007530 | - | - | - | - |  |
| Hypothetical protein | wHa_06300 | WD0742 | WRi_007340 | - | - | - | - |  |
| Hypothetical protein | wHa_06820 | WD0804 | WRi_007730 | - | - | - | *Flavobacterium columnare* ATCC 49512 | 1e-24 |
| Hypothetical protein | wHa_06830 | WD0807 | WRi_007760 | - | - | - | planctomycete KSU-1 | 1e-15 |
| Hypothetical protein | wHa_09840 | WD1178 | WRi_011530 | - | - | - | - |  |
| Hypothetical protein | wHa_01520 | WD0192 | WRi_001800 | - | pseudo (pos. side) | - | Candidatus *Regiella insecticola* R5.15 | 2e-12 |
| Hypothetical protein | wHa_03320 | WD0443 | WRi_003020 | - | pseudo (pos.) | - | B-group *Wolbachia* strains | 7e-50 |
| Hypothetical protein | wHa_09820 | WD1176 | WRi_011510 | - | pseudo (pos.) | - | SAR116 cluster HIMB100 | 6e-23 |
| Hypothetical protein | wHa_00850 | WD0171 | WRi_000840 | pseudo (pos.) | pseudo (pos. side) | + (Ac, Am) | *Anaplasma marginale* (all strains) | 9e-11 |
| Hypothetical protein | wHa_04680 | WD0382 | WRi_004090 | pseudo (pos. side) | pseudo (not pos.) | + | *Ehrlichia canis* str. Jake | 0 |
