# Supplementary material for: Comparative Genomics of Wolbachia and the Bacterial Species Concept
Source: PLoS Genet. 2013 Apr 4;9(4):e1003381. doi: 10.1371/journal.pgen.1003381 (PMC3616963; doi:10.1371/journal.pgen.1003381)
Supplement: Table S6 — B-group specific genes. All genes found to be present in all supergroup B strains, while being absent from all supergroup A and D strains are listed with locus tag numbers. For those genes where a pseudogenized homolog could be identified in either supergroup B or D, “pos.” indicates that the homolog is present in a region of synteny between the genomes, whereas “Not pos.” indicates no synteny around the detected homolog. If a homolog could be identified in the Anaplasmataceae family, the members containing the gene are noted: Ac, Anaplasma centrale, Am, Anaplasma marginale, Eca, Ehrlichia canis, Ech., Ehrlichia chaggeensis. When applicable, the best significant blast hit outside the Wolbachia group is indicated. Note that the murC gene in supergroup A and D is a complete version. (DOCX) [file pgen.1003381.s017.docx]

**Supplementary Table S6**. **B-group specific genes.**

| **Protein** | **wNo** | **wPip** | **wAlbB** | **A-group** | **wBm** | **Anaplasmataceae** | **Best hit (nr)** | **E-value** |
| --- | --- | --- | --- | --- | --- | --- | --- | --- |
| Ankyrin repeat domain protein | wNo_03920 | WPa_0729 | WALBB_770017 | - | - | - | *Oreochromis niloticus* | 4e-10 |
| Ankyrin repeat domain protein | wNo_03930 | WPa_0730, WPa_0731, WPa_1379 | WALBB_770019, WALBB_780004 | - | - | - | Candidatus *Amoebophilus asiaticus* 5a2 | 4e-19 |
| Ankyrin repeat domain protein | wNo_10020 | WPa_0383 | WALBB_1250006 | - | - | - | *Trichomonas vaginalis* G3 | 3e-46 |
| MurC-like protein | wNo_09540 | WPa_0207 | WALBB_1170007 | + | + | + (Ac, Am) | A-group *Wolbachia* strains | 5e-97 |
| Putative outer membrane protein | wNo_07980, wNo_07990 | WPa_1137, WPa_1138, WPa_1139 | WALBB_1020015, WALBB_1020017 | - | pseudo (not pos.) | - | *Wolbachia* strain wBm | 2e-11 |
| Putative membrane protein | wNo_04540 | WPa_0794 | WALBB_170002 | - | - | - | - |  |
| Putative membrane protein | wNo_02780 | WPa_0576 | WALBB_690002 | - | + (pos.) | - | *Wolbachia* strain wBm | 3e-24 |
| Putative triglyceride lipase | wNo_03710 | WPa_0707 | WALBB_790003 | - | pseudo (pos.) | - | *Paramecium tetraurelia* strain d4-2 | 9e-13 |
| Putative lipoprotein | wNo_06400 | WPa_1082 | WALBB_970012 | - | - | - | *Sideroxydans lithotrophicus* ES-1 | 3e-13 |
| Putative acetyltransferase | wNo_09250 | WPa_0046 | WALBB_1210001 | - | - | - | *Rickettsia bellii* | 1e-33 |
| Hypothetical protein | wNo_00300 | WPa_0096 | WALBB_360008 | - | - | - | A-group *Wolbachia* strains | 2e-10 |
| tRNA pseudouridine synthase B | wNo_04870 | WPa_0829 | WALBB_570005 | - | pseudo (pos.) | + | *Ehrlichia canis* str. Jake | 3e-71 |
| Hypothetical protein | wNo_00940 | WPa_0061 | WALBB_330011 | - | - | - | - |  |
| Hypothetical protein | wNo_01430 | WPa_0472, WPa_0704 | WALBB_790006 | - | - | - | - |  |
| Hypothetical protein | wNo_01610 | WPa_0495 | WALBB_900008 | - | - | - | wBm, A-group *Wolbachia* strains | 4e-12 |
| Hypothetical protein | wNo_01790 | WPa_0512 | WALBB_910004 | - | - | - | *Orientia tsutsugamushi* str. Ikeda | 1e-141 |
| Hypothetical protein | wNo_02430 | WPa_0666 | WALBB_250008 | - | - | - | - |  |
| Hypothetical protein | wNo_02440 | WPa_0665 | WALBB_250007 | - | - | - | - |  |
| Hypothetical protein | wNo_03400 | WPa_0644 | WALBB_720007 | - | - | - | - |  |
| Hypothetical protein | wNo_08110 | WPa_1153 | WALBB_1030007 | - | - | - | - |  |
| Hypothetical protein | wNo_10420 | WPa_1367 | WALBB_840006 | - | - | - | - |  |
| Hypothetical protein | wNo_10950, wNo_10970 | WPa_1283, WPa_1286 | WALBB_1140018 | - | - | - | *Orientia tsutsugamushi* str. Boryng | 2e-08 |
| Hypothetical protein | wNo_08580 | WPa_1202 | WALBB_1080005 | - | pseudo (not pos.) | + (Eca, Ech) | *Ehrlichia canis* str. Jake | 3e-28 |
| Hypothetical protein | wNo_08870 | WPa_1238 | WALBB_1100014 | - | pseudo(not pos.) | - | - |  |
